# Statistical analysis of comparative tumor growth repeated measures experiments in the ovarian cancer patient derived xenograft (PDX) setting

**DOI:** 10.1038/s41598-021-87470-x

**Published:** 2021-04-13

**Authors:** Ann L. Oberg, Ethan P. Heinzen, Xiaonan Hou, Mariam M. Al Hilli, Rachel M. Hurley, Andrea E. Wahner Hendrickson, Krista M. Goergen, Melissa C. Larson, Marc A. Becker, Jeanette E. Eckel-Passow, Matthew J. Maurer, Scott H. Kaufmann, Paul Haluska, S. John Weroha

**Affiliations:** 1 Division of Computational Biology, Department of Quantitative Health Sciences, Mayo Clinic, 200 First Street SW, Rochester, MN 55905 USA; 2 Division of Medical Oncology, Department of Oncology, Mayo Clinic, 200 First Street SW, Rochester, MN 55905 USA; 3 Division of Gynecologic Oncology, Department of Obstetrics and Gynecology, Mayo Clinic, 200 First Street SW, Rochester, MN 55905 USA; 4 Department of Molecular Pharmacology and Experimental Therapeutics, Mayo Clinic, 200 First Street SW, Rochester, MN 55905 USA; 5 Division of Oncology Research, Department of Oncology, Mayo Clinic, Rochester, 55905 MN USA; 6 Present Address: Division of Subspecialty Care for Women’s Health, Gynecologic Oncology, Women’s Health Institute, Cleveland Clinic, Cleveland, OH 44126 USA; 7 Present Address: Department of Pediatrics, The Children’s Hospital of Philadelphia, Philadelphia, PA 19104 USA; 8 Present Address: Blueprint Medicines, 45 Sidney St., Cambridge, MA 02139 USA; 9 Present Address: Bristol-Meyers Squibb, 3401 Princeton Pike, Lawrenceville, NJ 08648 USA

**Keywords:** Cancer, Oncology

## Abstract

Repeated measures studies are frequently performed in patient-derived xenograft (PDX) models to evaluate drug activity or compare effectiveness of cancer treatment regimens. Linear mixed effects regression models were used to perform statistical modeling of tumor growth data. Biologically plausible structures for the covariation between repeated tumor burden measurements are explained. Graphical, tabular, and information criteria tools useful for choosing the mean model functional form and covariation structure are demonstrated in a Case Study of five PDX models comparing cancer treatments. Power calculations were performed via simulation. Linear mixed effects regression models applied to the natural log scale were shown to describe the observed data well. A straight growth function fit well for two PDX models. Three PDX models required quadratic or cubic polynomial (time squared or cubed) terms to describe delayed tumor regression or initial tumor growth followed by regression. Spatial(power), spatial(power) + RE, and RE covariance structures were found to be reasonable. Statistical power is shown as a function of sample size for different levels of variation. Linear mixed effects regression models provide a unified and flexible framework for analysis of PDX repeated measures data, use all available data, and allow estimation of tumor doubling time.

## Introduction

Mouse models of human cancer are a useful tool to compare effectiveness of novel therapeutics on tumor regression or growth delay. A common study design is a repeated measures experiment in which tumor burden is measured repeatedly in the same mice at regular intervals at two or more time-points, e.g., once or twice weekly, until the planned study endpoint or until mice must be sacrificed due to disease burden. Cancer cell lines have historically been implanted into mice to generate a tumor. However, the passage of immortalized cell lines in tissue culture in vitro generates a population that may be far removed, genetically and biologically, from the originating human tumor. In recent years, patient-derived xenograft (PDX) models have been established with the goal of more closely representing the genetics and biological behavior of patient tumors^1–6^. This has enabled a more diverse array of tumors to be studied. However, this comes with analytical challenges of added variability in a variety of tumor parameters as well as in the shapes of tumor growth curves.

Statistical analysis of the data from these types of xenograft studies is commonly carried out by computing average percent change from baseline for each study arm at each timepoint. These values are then plotted as a function of time for each arm, and analysis of variance (ANOVA) or t-tests are performed at each measurement time point so that the assumption of independent observations is met. However, this statistical testing strategy has several shortcomings^7^. Statistical power is reduced because tests use data at only one time point, and power changes at each time point because of varying sample sizes caused by mouse attrition. Multiple tests used to assess the same hypothesis lead to risk of false discoveries due to multiple comparisons, with type I error rate more than double the commonly specified 5%^8^. In addition, this approach can result in discordant results at different experimental time points, leading to confusion regarding appropriate conclusions. Finally, the percent change response variable mixes the multiplicative and additive scales, obscures possible imbalances between treatment arms at baseline, and alters the form of the correlation between observations. An alternative approach is the repeated measures ANOVA, which uses data over the entire study period in a single hypothesis test while accounting for the correlation between multiple observations per mouse. However, the classic implementation of this analysis found in common laboratory software packages is severely limited by the requirement of equally spaced and complete data for each mouse over the entire study period, as well as the assumptions of equal variance at all time points and equal correlation between repeated measurements regardless of the length of time (lag) between the two observations.

The linear mixed effects regression model addresses these concerns and provides a unified repeated measures analytical framework that (i) provides flexibility in the length of follow up per mouse, and (ii) allows both the variance and the correlation between repeated measurements from the same mouse to change over time. It utilizes data from all time points in a single test per hypothesis, has greater statistical power than the methods in the previous paragraph, and controls the type I error rate at the specified level. This modeling framework has demonstrated utility in analyzing repeated measures comparative drug experiments in mouse models^7–10^. Other common names for this analytical framework include multilevel regression, hierarchical regression, and growth curve analysis. The following steps can be used to implement this strategy^11,12^: (1) fit the mean model, (2) determine an appropriate structure for the variation between mice and correlation within mice, (3) re-fit the model with the chosen variance structure, (4) perform hypothesis testing and inference. Decision making in each of these steps is not always straightforward because multiple metrics of model fit exist, and at times point to differing decisions. Our application of these steps has coalesced through our experience analyzing data from > 45 PDX models through collaborations within the Mayo Clinic Ovarian SPORE grant to evaluate new treatment therapies, and through a clinical trial in which a patient’s PDX is used to choose between four different standard-of-care therapies for platinum resistant ovarian cancer (clinicaltrials.gov ID: NCT02312245).

Our objective in this manuscript is to provide, via a Case Study, graphical and numerical summaries and their interpretation that are useful for guiding statistical modeling decisions in a manner that enables others to apply these modeling steps to their own data. We aim to present an analytical strategy in a manner that makes the concepts accessible to a general research audience, while providing sufficient information for a statistician to apply the strategy. A concise overview of the modeling steps and considerations is provided in Supplemental Table 1. Throughout this work, PDX model is used to refer to all mice with tumor originating from a single patient. Alternatively, the statistical model refers to the analytical method used to perform hypothesis testing and inference.

## Case study

**Figure 1:**
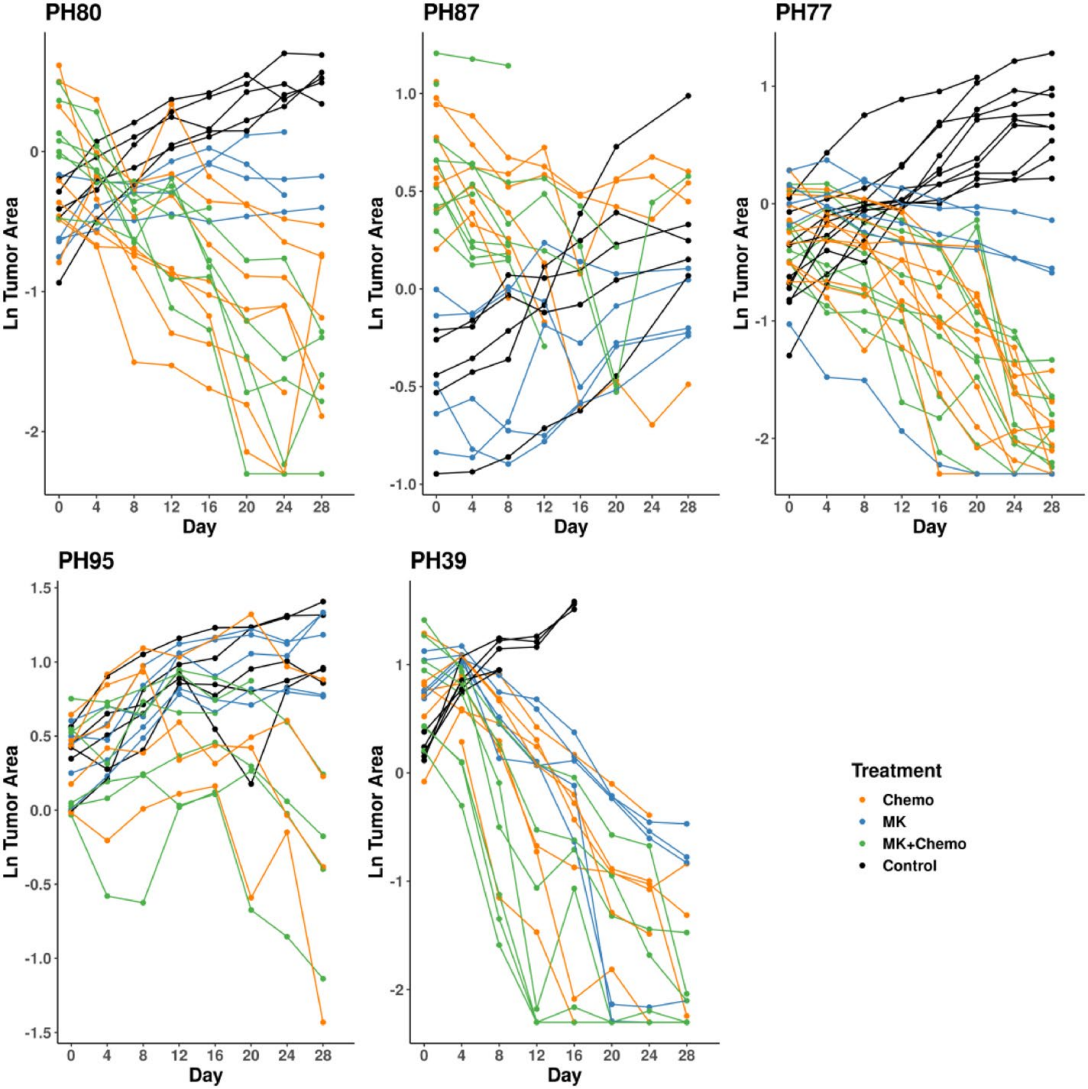
Profile plots on the natural log scale. Profile plot (per-mouse intraperitoneal tumor area trajectories measured via ultrasound) for all five Case Study PDX models on the natural log scale used for modeling. Coloring indicates drug arm: control (diluent), chemo (carboplatin + paclitaxel), the PARPi niraparib (MK), or chemo + MK (carboplatin + paclitaxel + MK). Lines connect observations from the same mouse provide a visual check of balance at baseline (day = 0), a sense of whether growth trajectories are straight or curved (functional form), and the magnitude of between mouse variation and attrition. Per arm sample sizes at day 0 for each PDX model are as follows: PH80: control n = 5, chemo n = 8, MK n = 5, MK + chemo n = 7; PH87: control n = 5, chemo n = 9, MK n = 5, MK + chemo n = 7; PH77: control n = 10, chemo n = 10, MK n = 5, MK + chemo n = 9; PH95: control n = 5, chemo n = 6, MK n = 5, MK + chemo n = 6; PH39: control n = 5, chemo n = 8, MK n = 5, MK + chemo n = 7.

Our previously published comparative tumor growth repeated measures study evaluating a poly (ADP-ribose) polymerase inhibitor (PARPi)^13^ utilized five treatment-naïve high-grade serous ovarian PDX models. For each PDX model, 5–10 mice per treatment arm as specified in Fig. 1 were randomized to treatment with control (diluent), chemotherapy (carboplatin + paclitaxel), the PARPi niraparib (MK-4827; abbreviated MK), or chemotherapy + MK (carboplatin + paclitaxel + MK) in a factorial treatment structure, where dosing and schedule are described previously^13^. Within each PDX model, mice were randomized to a treatment arm once the tumor reached 0.5–1 cm^2^. Tumor burden was assessed via ultrasound; the largest intraperitoneal tumor area was measured twice weekly for 28 days, generating up to m = 8 data points per mouse, as described elsewhere^13,14^. The primary hypothesis was to determine whether the chemotherapy + MK was more effective than chemotherapy alone. Secondary hypotheses included comparisons of each arm to diluent to determine activity. We refer the reader to our previous publication for further experimental details and biological interpretation of the data^13^. All experiments were reviewed and approved by the Mayo Clinic Institutional Animal Care and Use Committee (IACUC) and conducted in accordance with their guidelines, and was carried out and reported^13,14^ in accordance with the ARRIVE guidelines. Generation of the PDX models was reviewed and approved by the Mayo Clinic Institutional Review Board, all methods were carried out in accordance with their guidelines, and informed consent was obtained from patients.

## Results

Prior to beginning the modeling steps, we examine the data. Jitter plots of baseline tumor area by treatment arm for each PDX model are reviewed. These demonstrate approximate balance in most cases (Supplemental Fig. 1). Some notable deviations include smaller tumors in the PH39 and PH77 diluent arms as well as smaller tumors in the PH80 and PH87 diluent and MK arms. Considering the small sample sizes, we generally observe some variation between arms in the median baseline tumor area, though the range is generally similar. Imbalance between arms must be considered when interpreting results.

Growth trajectories generally follow the same overall shape within a PDX model on the raw (Supplemental Fig. 2) and natural log analysis scale (Fig. 1), both within and between treatment arms. There is both *between mouse variation* resulting in a distribution of trajectories for each treatment arm, and *within mouse variation*, i.e., observations vary about that mouse’s overall trajectory due to growth spurts and measurement error. As a result, measurements from the same mouse are more alike than those taken on different mice, and within a mouse, observations that are closer together in time are more alike than those that are further apart in time. In other words, they are correlated, or have covariation; correlation is simply covariance scaled by variation to be on the (− 1, 1) scale.

*Step 1: Fit the mean model* Trajectories on the natural log scale for PH80, PH77, and PH87 appear mostly straight, with possible evidence of a “floor” in PH80 and PH77 due to complete regression in some mice on the chemotherapy and chemotherapy + MK arms, and possible curvature effect of treatment in PH87 driven by a few of the mice (Fig. 1). Both PH95 and PH39 have curvature characteristics. PH95 exhibits slow growth initially followed by slowed growth (diluent, MK), or slow growth followed by gradual tumor regression midway through the experiment (chemotherapy, chemotherapy + MK). PH39 exhibits immediate and continued response resulting in disappearance of the tumor for some mice in the chemotherapy + MK arm, immediate growth followed by tumor regression in the chemotherapy and MK arms, and growth throughout the experiment in the diluent arm; there is evidence of a floor in all three active arms. The following were used to choose the mean model functional form for each PDX model: (i) plots of overlaid observed and predicted mean model plots, (ii) residual plots, and (iii) numerical measures of relative model fit quality [Akaike information criterion (AIC), and Bayesian information criterion (BIC)], all from models fit with Maximum Likelihood (ML) assuming independence^15^. For a model that describes the data well, the model predicted trajectory should be similar to the observed trajectories, and the ideal residual plot should have no pattern over time. Smaller AIC and BIC statistics indicate better fits to the data; while AIC favors more complex models, BIC includes a penalty for the number of parameters estimated so tends to favor simpler models.

In PH80, straight predicted lines (Supplemental Fig. 3) describe the observed trajectories well for all arms with fitted lines overlaying the observed datapoints. While the MK and diluent arm residual plots have curved trends across time, the AIC and BIC (Table 1) criteria both suggest that higher order polynomial terms (i.e. day squared, day cubed) do not add substantially to the model fit relative to the “cost” of estimating these extra parameters. In PH87, straight predicted lines describe the observed trajectories well for all arms, and while the chemotherapy arm residual plot shows slight curvature and the AIC criteria prefers a quadratic model (i.e. inclusion of day squared), the BIC criteria suggests a straight fit is preferred. In contrast, for PH77, curved models follow observed trajectories better for chemotherapy and chemotherapy + MK arms, residual plots show no trend for a quadratic or more complex models for these arms, and AIC and BIC both indicate a quadratic model is preferred. In PH95 similar findings hold for all arms. In both PH77 and PH95, AIC and BIC criteria indicate that a shared quadratic term between all arms is sufficient, and that allowing the quadratic trend to vary by arm is not worth the increase in model parameters. In PH39, the cubic (i.e. inclusion of day cubed) with interaction (i.e., a different coefficient for day cubed for each treatment arm) best describes the pattern of growth followed by regression in the chemotherapy and MK arms, and residual plots also demonstrate that at least a cubic model is needed to remove trends in the residuals for those arms. While the AIC indicates a cubic model is best, the BIC indicates a straight model is sufficient. While our general philosophy is to give preference to more parsimonious models, in this case, we chose the cubic model since the growth followed by regression was consistent across all mice in the chemotherapy and MK arms. Thus, our final mean model selections were straight for PH80 and PH87, quadratic for PH77 and PH95, and cubic for PH39 as indicated in bold font in Table 1.

**Table 1:** ML information criteria for choosing mean model.

ML IC	PDX	Form of mean model				
		Cubic + interaction	Cubic	Quadratic + interaction	Quadratic	Straight + interaction
AIC	PH80	199.3	194.1	193	191	189.2*
	PH87	124.5	121	119.1	115.8*	**117.5**
	PH77	388.7	383.2	381.6	377*	383.1
	PH95	164.8	160.8	159	157*	173.4
	PH39	336.5	333.9*	334.2	339	339.6
BIC	PH80	253.8	239	234.8	223.1	218.1*
	PH87	176	163.4	158.5	146.1	144.8*
	PH77	449.7	433.4	428.3	412.9*	415.4
	PH95	217	203.8	198.9	187.6*	201
	PH39	389.7	**377.7**	374.8	370.3	367.7*

Lower values are better for both AIC and BIC, and the lowest per PDX model is indicated by *. AIC favors more complex models, while BIC includes a penalty for the number of parameters estimated so tends to favor more simple models with fewer parameters. Bold font indicates the chosen mean model.

*ML* Maximum likelihood, *AIC* Akaike information criterion, *BIC* Bayesian information criterion, *PDX* Patient derived xenograft.

**Figure 2:**
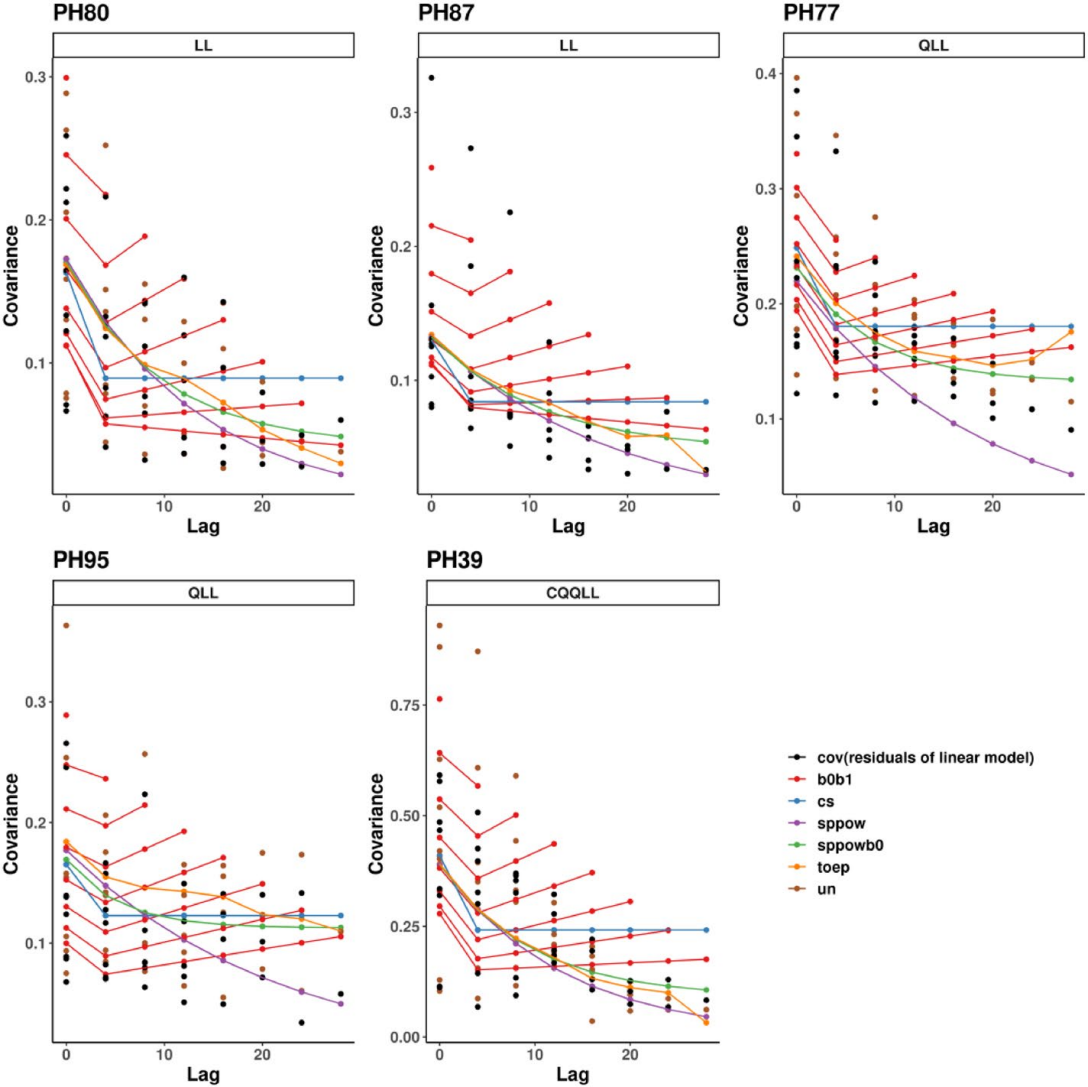
Covariance structures. Overlaid observed and estimated covariance structures for each PDX model with the chosen mean model. The x-axis is the lag (or distance in time) between two observations. The y-axis is variance (at lag 0) and covariance (at lags greater than 0). Covariance estimates based on ML independence model residuals are indicated with black dots. The remaining structures are estimated via REML. Unstructured estimates are indicated with brown dots, except for PH87 where this model did not converge. An unstructured (UN) covariance matrix imposes no restrictions or assumptions, allowing estimation of separate variance and covariance parameters between all possible pairs of time points, thus requiring many parameters, and making inefficient use of available data. RE (labeled b0b1) estimates are indicated by multiple red lines due to the dependence of the variance and covariance on both lag and the precise point in time. Sp(pow) assumes the covariance declines smoothly to 0, demonstrated by the purple lines tapering to near 0 by lag 28. Similarly, the sp(pow) + RE (labeled sppowb0) assumes the covariance tapers smoothly to the between mouse variance (the random effect). CS assumes the same covariance regardless of lag as seen by the flat line for lags 1 and greater; the taper in the unstructured points do not support this assumption. The Toep estimates generally decrease as lag increases, but not as quickly or smoothly as the sp(pow) or sp(pow) + RE, and for PH77 increases toward the end of the study. The fact that sp(pow), sp(pow) + RE and Toep depend only on lag and not actual study time is evident from one point at each lag. All of these traits can be observed in the printed variance estimates as well (Supplemental Table 4).

*Step 2: Determine variance and covariance structure* Once the general form of the mean model was fixed, we evaluated the covariance structure between the repeated measurements taken on the same mouse. The following tools were used: i) semivariogram plots of observed variance and covariance as a function of distance between two observations (lag) overlaid with estimates from the candidate structures, ii) Restricted (or Residual) Maximum Likelihood (REML) AIC and BIC criteria, and iii) estimated covariance matrices with variances on the diagonal and covariances on the off-diagonal^11,16,17^. Figure 2 shows the relative magnitude of variance and covariance estimates of *ln(A*_*t*_*)* values as a function of lag using unstructured (UN), spatial(power) [sp(pow)], spatial(power) plus random effect [sp(pow) + RE], Toeplitz (Toep), compound symmetric (CS), and random effects (RE) structures in the Case Study PDX models. These differ according to the nature of assumptions imposed and number of parameters required (Supplemental Tables 2 and 3)^11,17^. A reasonable structure that describes the covariance well should have estimates similar to the UN estimates and represent the trends in variance and covariance well. AIC and BIC criteria are as described for the mean model.

Figure 2 shows that the UN variance (at lag = 0) and covariance (at lags > 0) estimates (available for all but PH87 in which the UN model did not converge) are similar to estimates based on the ML independence model residuals; either can be used for evaluating the covariance structure. Variance estimates range from approximately 0.05 or 0.1 in all PDX models to around 0.3 or 0.4 in most PDX models and as high as 0.8 in PDX PH39, but the increasing trend over time is not consistent in any PDX model (Supplemental Table 4). The covariance estimates taper off as lag increases for all PDX models, indicating that the further apart the observations are in time, the less correlated they are. For example, in PH80, the UN estimates range up to 0.25 for observations 4 days apart and up to only 0.05 for observations 24 days apart.

To decide the optimal structure to use for hypothesis testing, we balanced the goal of parsimony with how well the structure follows the UN estimates, focusing on major trends due to the small sample size and number of parameters being estimated. REML AIC values indicate the UN structure is best for all PDX models where this converges, while BIC values indicate that fewer parameters can achieve good fits (Table 2). BIC indicates sp(pow) is best for PH87, PH80, PH39, with similar BIC values indicating sp(pow) + RE is a reasonable second choice; sp(pow) + RE is best for PH77, and a RE model best for PH95, with sp(pow) being a reasonable second choice for each. In these data, we would choose the BIC preferred structure if making inferences for a single PDX model, and sp(pow) if making inferences for all PDX models in the same manuscript for ease of reporting.

**Table 2:** REML information criteria for choosing variance/covariance structure, comparable since the mean model is fixed for each PDX model across the variance/covariance structures.

# Covariance parameters		sp(pow)	UN	Toep	CS	sp(pow) + RE	RE
		2	36	8	2	3	4
AIC	PH80 (straight)	***106***	82.6*	114.5	139.3	107.3	112.4
	PH87 (straight)	36.4*		45	70.3	37.5	47.6
	PH77 (quadratic)	*151.6*	121.3*	154.1	201.2	**147.6**	190.7
	PH95 (quadratic)	*61.6*	36*	58.8	69.9	57.4	**45.7**
	PH39 (cubic)	***292.6***	265.6*	303	326	294.2	311.9
BIC	PH80 (straight)	108.4*	126.4	124.2	141.7	111	117.3
	PH87 (straight)	39.1*		56	73	41.6	53.1
	PH77 (quadratic)	*154.7*	176.2	166.3	204.3	152.2*	196.8
	PH95 (quadratic)	*63.8*	75.3	67.5	72.1	60.6	50.1*
	PH39 (cubic)	295*	3094	312.8	328.4	297.8	316.7

Lower values are better for both AIC and BIC, and the lowest is indicated by *. As with the mean models, AIC favors more complex models, while BIC includes a penalty for the number of parameters estimated so tends to favor more simple models with fewer parameters. Bold font indicates our choice if that was the only PDX model being considered; italic font indicates the common choice for simplified reporting.

*REML* Restricted (or residual) maximum likelihood, *PDX* Patient derived xenograft, *AIC* Akaike information criterion, *BIC* Bayesian information criterion, *Sp(pow)* Spatial(power), *UN* Using unstructured, *Toep* Toeplitz, *CS* Compound symmetric, *RE* Random effects.

**Figure 3:**
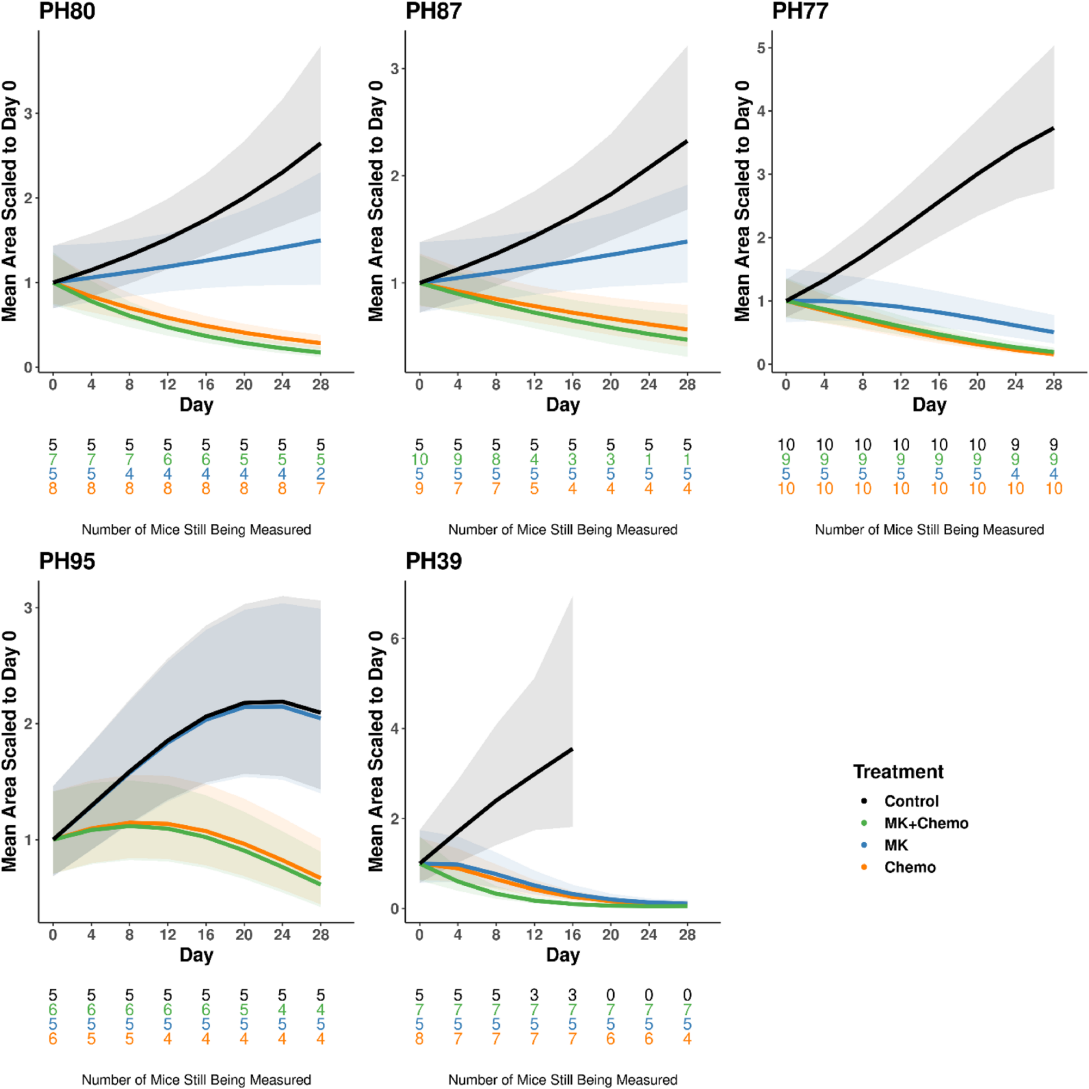
Model estimates of growth trajectories for each PDX model. Coloring indicates drug arm. Predicted lines are the average estimates computed from the statistical model fixed effects, relative to the arm specific baseline estimate. The forms of the statistical mean models are the following: PH80, PH87: straight; PH77, PH95: quadratic; PH39: cubic. Shading indicates 95% confidence intervals. The number of mice under observation at each time point for each arm is indicated below the x-axis as a function of time, where text color indicates drug arm.

*Step 3: Re-fit the statistical model* Once the correlation structure is set, the statistical models are re-fit with the chosen mean and covariance structures using REML. The final figures reported in a manuscript include the predicted mean values from the statistical model together with shading to indicate 95% confidence intervals (Fig. 3). Predicted mean values are scaled relative to the predicted value at day 0 within each arm to align with the customary presentation of such data in the literature. Since mouse dropout can reflect moribund endpoints, e.g., due to drug toxicity, or excessive tumor burden from a lack of efficacy, the sample size remaining at each time point is indicated along the x-axis. Mice had to be removed in all PDX models, though losses were most severe in PH87.

*Step 4: Hypothesis testing* The primary hypothesis tests whether adding MK to chemotherapy improves tumor regression. The chemotherapy + MK trajectory was statistically significantly different from chemotherapy alone for PH39 only via a test of coincident curves (Table 3). This difference was driven by the intercept and quadratic terms (*p* = 0.0090, 0.0255 respectively) rather than the linear slope (*p* = 0.2309). The similarity in tumor regression rates and the shift in mean due to delayed response in the chemotherapy and MK alone arms is evident visually (Fig. 3).

**Table 3:** Hypothesis testing results using the chosen mean functional form for each PDX model and sp(pow) covariance structure for all PDX models.

PDX	Comparison		*P* values				Conclusion
			Coincident curves (2df)	Intercept (AUC)	Slope	Quad	
PH80	Diluent	MK	0.0590	0.0491	0.1299		Marginal activity
		Chemo	< 0.0001	< 0.0001	< 0.0001		Shows activity
		Chemo + MK	< 0.0001	< 0.0001	< 0.0001		Shows activity
	Chemo	Chemo + MK	0.2715	0.9441	0.1038		Not better than chemo
PH87	Diluent	MK	0.0989	0.2042	0.0757		Marginal activity
		Chemo	< 0.0001	0.0164	< 0.0001		Shows activity
		Chemo + MK	< 0.0001	0.0747	< 0.0001		Shows activity
	Chemo	Chemo + MK	0.7616	0.5204	0.5437		Not better than chemo
PH77	Diluent	MK	< 0.0001	0.0023	< 0.0001		Shows activity
		Chemo	< 0.0001	< 0.0001	< 0.0001		Shows activity
		Chemo + MK	< 0.0001	< 0.0001	< 0.0001		Shows activity
	Chemo	Chemo + MK	0.7587	0.9648	0.4575		Not better than chemo
PH95	Diluent	MK	0.9972	0.9865	0.9420		No activity
		Chemo	0.0007	0.0139	0.0008		Shows activity
		Chemo + MK	0.0001	0.0068	0.0002		Shows activity
	Chemo	Chemo + MK	0.9377	0.7845	0.7914		Not better than chemo
PH39	Diluent	MK	< 0.0001	0.0005	0.0030	0.8080	Shows activity
		Chemo	< 0.0001	< 0.0001	0.0021	0.9792	Shows activity
		Chemo + MK	< 0.0001	< 0.0001	0.0006	0.3725	Shows activity
	Chemo	Chemo + MK	0.0290	0.0090	0.2309	0.0255	Improvement above chemo in mean, not growth rate

Comparisons of Chemo versus Chemo + MK assess the primary question of the study, i.e., whether addition of the PARPi MK to chemo improves the performance. Comparisons with diluent assess the secondary question of whether each arm shows activity. The test of coincident curves used 2 degrees of freedom for PH80, PH87, PH77, PH95, and 3 degrees of freedom for PH39.

*PDX* Patient derived xenograft, *Sp(pow)* Spatial(power), *Chemo* Chemotherapy, *MK* MK-4827, *PARPi* Polymerase inhibitor, *AUC* Area under the curve.

The secondary hypothesis tests assess activity of each arm and are performed as part of the evaluation of experiment conduct, i.e., comparison of each arm with diluent. The tests of coincident curves comparing chemotherapy or chemotherapy + MK trajectories to diluent trajectories were statistically significant for all PDX models, indicating that these arms had activity in all PDX models (*p* < 0.05) (Table 3), i.e., that chemotherapy had the expected result. In all these comparisons, *p* values indicate that trajectories differed in both the intercept (with marginal significance in PH87) and slope. This can be seen visually by diverging lines and lack of overlap in confidence bands (Fig. 3). The MK versus diluent comparison was statistically significant for PH77 and PH39, marginally statistically significant for PH80 and PH87, and not statistically significant for PH95, indicating MK had activity in some but not all PDX models. In PH80, the marginal significance of MK versus diluent is driven by the intercept (*p* = 0.0491) rather than the slope (*p* = 0.1299). In contrast, the marginal significance of this comparison in PH87 is driven primarily by the slope (*p* = 0.0757) rather than the intercept (*p* = 0.2042). The significant differences in PH77 and PH39 were driven by both the intercept and slope (*p* < 0.05). Marginal and nonsignificant comparisons are visualized by overlapping confidence bands (Fig. 3).

### Sample size considerations

**Figure 4:**
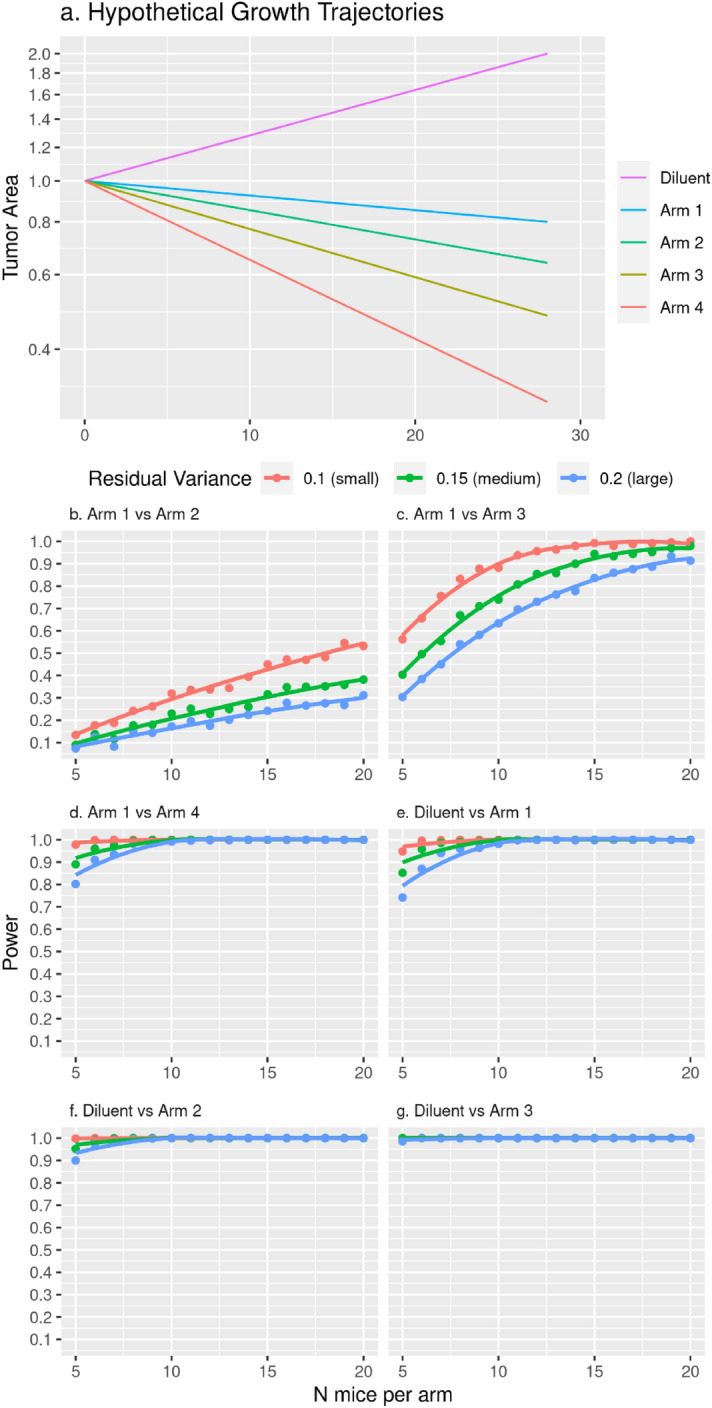
Statistical power as a function of residual variance and sample size. Straight growth trajectories were simulated with sp(pow) covariance structure at small (0.1), medium (0.2) and large (0.3) residual variance values representing approximate Q25, Q50 and Q75 percentiles of the NCT02312245 clinical trial PDX model *ln*(*A*_*t*_) residual variance distribution, sp(pow) structure with parameter 0.98, and two-sided type I error rate of α = 0.05. Hypothetical growth trajectories are shown in panel (**a**) for a diluent arm with tumor growing to 2cm^2^ day 28 area and active arms shrinking to 0.8cm^2^, 0.64cm^2^, 0.48cm^2^, and 0.3cm^2^ day 28 area. Tumor area is plotted on the natural log scale and labeled on the raw cm^2^ scale. We calculated statistical power for the specified two degree of freedom coincident curve hypothesis tests in panels (**b**–**g**) as the proportion of times out of 500 simulated datasets that the p-value was less than 0.05. Panels (**b**–**d**) represent hypothetical primary hypothesis tests versus arm 1, and panels (**e**–**f**) represent hypothetical secondary hypothesis tests versus diluent. Specifically, (**b**) arm 1 vs. arm 2 (day 28 area represents a 20% decrease from arm 1), (**c**) arm 1 vs. arm 3 (day 28 area represents a 40% decrease from arm 1), (**d**) arm 1 vs. arm 4 (day 28 area represents a 63% decrease from arm 1), (**e**) diluent vs. arm 1, (**f**) diluent vs. arm 2, (**g**) diluent vs. arm 3. Diluent vs. arm 4 has the same power as diluent vs. arm 3 so is not shown.

Because variability in size and response is greater with PDX models than with cell line xenograft models, we assessed statistical power for two straight coincident curve hypothesis test scenarios: (i) the primary hypothesis test comparing two active drug arms, and (ii) the secondary hypothesis test comparing an active drug arm to diluent (Fig. 4a). Five-hundred datasets, each containing hypothetical growth trajectories for one diluent and four active arms, were simulated for each of three levels of variability representing Q25, Q50 and Q75 variance estimates from the clinical trial PDX models as described in "Methods" section. For the primary hypothesis testing scenario, a comparison of hypothetical active arms 1 and 2 with day 28 area of 0.8 cm^2^ and 0.64 cm^2^, respectively, has less than 30% power with n = 10/arm even for the small variance (Fig. 4b). Comparison of hypothetical active arms 1 and 3 with day 28 area of 0.8 cm^2^ and 0.48 cm^2^, respectively, has 90%, 75%, and 65% with n = 10/arm for small, medium and large variances respectively (Fig. 4c). Power for additional two hypothetical active arms is shown in Fig. 4d. In the secondary hypothesis testing scenario, a comparison of hypothetical diluent and active arm 1 with day 28 areas of 2 cm^2^ and 0.8 cm^2^, respectively, has 100%, 99%, and 98% power with n = 10/arm with small, medium and large variances, respectively; power for this comparison is above 90% even with n = 5/arm (Fig. 4e) for half of the clinical trial PDX models since the medium variance represents the median variability observed among the clinical trial PDX models. Power for additional hypothetical active arms versus diluent is shown in Fig. 4f,g.

## Discussion

We have demonstrated step-by-step application of linear mixed effects regression models to analyze comparative drug studies in ovarian cancer PDX models in mice with repeated observations per mouse. Different metrics of model fit may point to different modeling choices, making the path forward unclear. Thus, we explain and demonstrate graphical and numerical summaries and their interpretation that we have found useful for evaluating model assumptions and provide guidance and rationale for modeling decisions. Regression functions with straight, and for some quadratic and cubic polynomial terms to accommodate curvature due to delayed drug response or other factors, described average tumor growth trajectories over the observed time period well. While variance increased somewhat over time for most PDX models, the increase was generally not consistent over time. Covariance and correlation tapered off as the length of time between two observations (lag) increased, and this covariation was not found to depend on the precise point in time. While the best structure varied across PDX models, it was possible to determine a single structure that was reasonable for all PDX models; sp(pow), sp(pow) + RE, and RE variance structures were found to be reasonable structures. Use of a multiple degree of freedom test of coincident curves was demonstrated to capture differences in growth/regression rates as well as mean shifts. Power and sample size calculations were provided.

As stated previously, the primary goal in these experiments is to compare the population average growth trajectory between drug arms with an appropriate covariance structure and appropriate handling of mice with incomplete follow-up data. The precise shape of the mean curve is a means to an end rather than the end goal here. To fit the mean model in the first step, assumptions must be made regarding the growth trajectory. Tumor growth has been shown to be multiplicative on the measurement scale and well described by the Gompertz, logistic and other functions, which are sigmoidal in shape^18–20^. In our study design we do not observe the full tumor life cycle and there are not sufficient data to confidently determine the precise ordinary differential equation growth model owing to: (i) a 0.1 cm^2^ ultrasound limit of detection, (ii) recording of tumor burden measurements begins at the time of randomization when the tumor is approximately 1 cm^2^, and (iii) tumors are not allowed to grow to their full size due to humane sacrifice of moribund mice according to IACUC requirements. We are not the first to face these limitations^21^. In spite of this, linear mixed effects models are a useful tool for comparing growth trajectories between treatment arms provided the regression model fits the data and statistical assumptions are satisfied as described herein, maximizing statistical power and controlling type I error rate^7–10,22–24^. Tumor area measured via ultrasound was used here as a measure of tumor burden in the response variable, though other measures may be used, including tumor volume or blood measurements^19,25,26^. We note that a straight growth trajectory on the natural log scale used herein for modeling is consistent with, but is not sufficient to imply, exponential growth and refer the reader to the following references on the topic, which we have found useful^18,19,27–29^. Nonlinear mixed effects models may be useful in settings where the growth model is known and/or sufficient data exist with which to estimate the needed parameters^20^, and incorporation of nonparametric smoothing splines or changepoints in settings where a growth model cannot be assumed and polynomial terms are not sufficient to explain the observed data^7,30,31^.

Performing hypothesis testing to determine inclusion of higher order polynomial terms has been shown to increase type I error rates of the primary drug growth rate comparison^32^. Thus, we rely on collective evidence from residual plots, alignment of observed and predicted growth trajectories, and information criteria from ML fits to determine whether higher order polynomial terms should be added to the model. REML, AIC and BIC may not be used to compare mean models. While the parameter estimates change once the final variance structure is chosen and REML estimates are generated, the ML estimates are sufficient for determining the overall functional form. Our overall philosophy is to accurately represent the data while maintaining parsimony and ease of interpretation, and we tend to prioritize BIC above AIC, as BIC generally favors simpler models with fewer parameters^17^. In general, we have noted that a given PDX model tends to exhibit similar functional forms across experiments. In addition, a straight or quadratic line on the natural log scale accurately describes growth trajectories for the majority of PDX models.

In determining the covariance structure, it is important to first focus consideration on covariance structures that are biologically plausible^16,17^. Due to the inability to randomize time in repeated measures experiments, it is common to observe: (i) variance that is either constant or increases with time, and (ii) covariance between measurements from the same mouse that tapers off as the length of time between measurements increases. Structures evaluated here accommodate these properties with exception of CS. RE, sp(pow), sp(pow) + RE and CS accommodate unequal time spacing. The RE model can be interpreted as fitting a linear regression line separately for each mouse, and then averaging the slopes and intercepts for a population level model (though the actual computational details are more sophisticated). There are many other possible structures available with modern software. Regardless of whether time is centered in the mean model, it must not be centered for computation of the covariance structure because some of the structures depend not only on lag, but on the actual value of time as well. In such structures, a centered time variable causes a bow tie effect that is not biologically plausible, with large variance at the beginning and end of the study, and smaller variance in the center of the study. The REML estimation should be used to estimate variance parameters for inference purposes because ML estimates of variance parameters are biased and lead to type I error rates that are higher than stated. The mean model must be held fixed to use AIC and BIC from REML estimation to compare the covariance structures. Guerin and Stroup^33^ showed that power and type I error rates are preserved if the chosen structure is close to the true structure. As with our mean modeling philosophy, we tend to prioritize BIC above AIC when choosing variance structures to align with the goal of parsimony.

We have reported the Case Study previously^13^ using *ln*(*A*_*t*_/*A*_0_) as the response variable, choosing to avoid the cubic model needed to accommodate the delay in growth for PDX model PH39 due to the complexity of interpretation, and relying on the estimated correlation matrix to capture the extra correlation induced by division of all data points by *A*_*0*_. Subsequent experience has taught us that the primary goal is to test for differences in drug activity rather than interpretation of the model parameters, making it more appealing to fit a more complex mean functional form adhering to basic statistical principles^34^. Note that while specific p-values do differ from the original report, the analyses herein support the same conclusions drawn in the original report of these data, though this may not always be the case.

In contrast to designs that utilize many PDX models, each with one mouse/treatment arm^3^, our experiments generally use one to five PDX models, each having multiple mice per treatment arm due to (i) the laboratory effort involved in reestablishing a frozen PDX model tumor for comparative drug or mechanism of action experiments, (ii) PDX models are variable in the speed at which they are reestablished, and (iii) our clinical trial (clinicaltrials.gov ID: NCT02312245) was designed to perform comparative drug experiments in a patient’s PDX model to choose between four different standard of care therapies for platinum resistant ovarian cancer at time of the patient’s recurrence. This strategy of analyzing multiple mice/arm across a modest number of different PDX models has been used frequently in our Ovarian SPORE research projects to evaluate promising agents, combinations of drugs, and/or mechanism of action before moving into clinical trials. This translational work is typically designed to investigate clinically relevant subsets of cancer, such as platinum sensitive or platinum resistant tumors, or in PDX models with specific tumor genotypes such as *BRCA1*- or *BRCA2*-mutant models. Multiple PDX models could be combined in a single statistical linear mixed effects model for global comparisons of drug efficacy between, for example, multiple platinum sensitive and multiple platinum resistant tumors as described by Guo et al.^10^, though they assume the straight trajectory holds for all PDX models.

Correctly modeling the statistical experimental design structure and how randomization is performed is important for accurate test statistics. Linear mixed effects models can accommodate a wide variety of treatment designs and randomization schemes to assess hypotheses in the most efficient manner possible. The factorial treatment design demonstrated here involves crossed treatment factors, with each level of chemotherapy (present/absent) being used in combination with each level of PARPi (present/absent). Nested treatment designs in which levels of one treatment are unique to levels of a different treatment are easily accommodated as well, as is analysis of covariance. While a completely randomized design randomization strategy was used herein, other randomization structures are easily accommodated, including randomized complete or incomplete block designs, split plot and others^17^. A randomized block design leverages the fact that variability is greater between PDX models than within a PDX model by performing randomization of all arms of interest within a PDX model; inference is made only regarding drug arms and no inference is made regarding the PDX models themselves. A split plot design is appropriate when two levels of randomization are performed. For example, random selection at the PDX model level of homologous repair (HR) proficient and deficient (HRD) models, followed by random allocation to drug arms within PDX model at the mouse level, with the goal of comparing the chemotherapy versus chemotherapy + PARPi delta between HR proficient and HRD PDX models; inference is made regarding both the type of PDX model and the drug arms.

**Figure 5:**
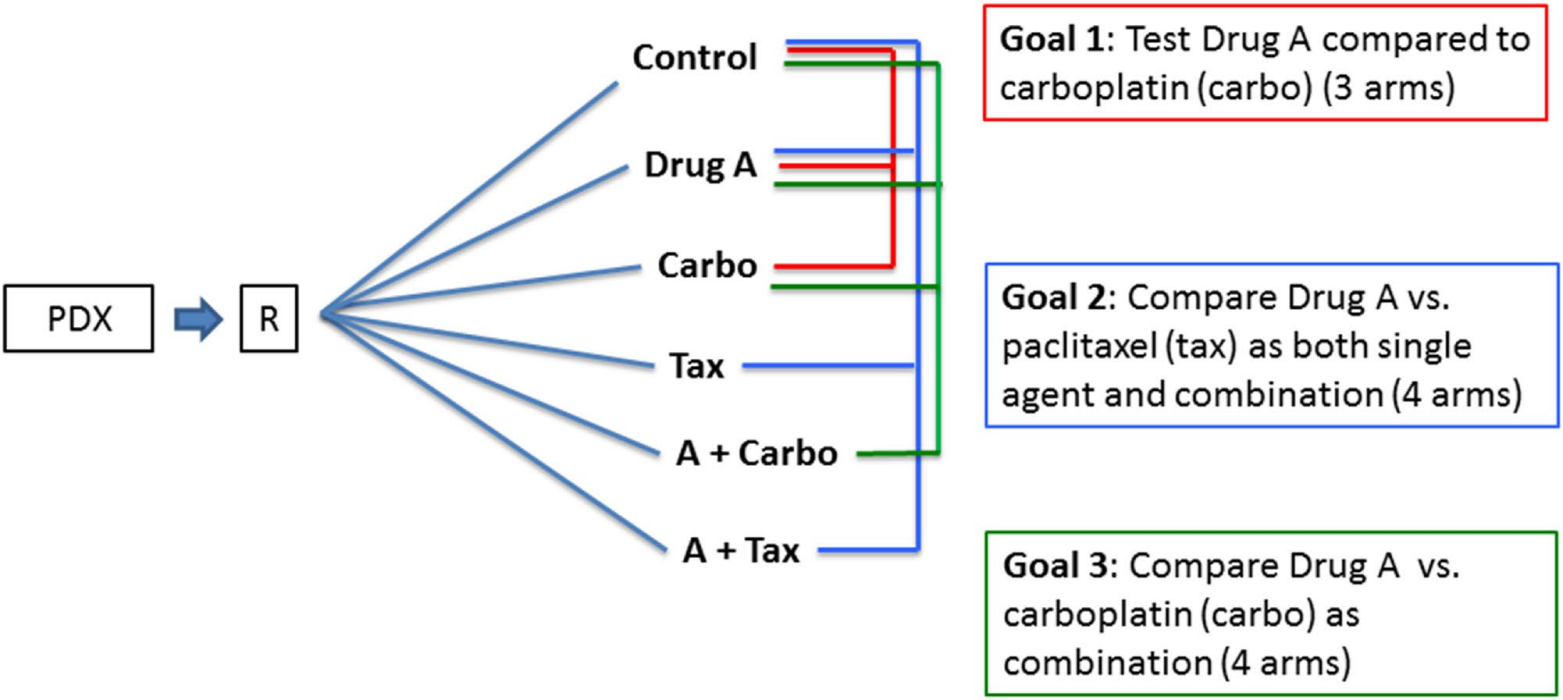
Experimental design. A series of three sequential studies was proposed to evaluate the goals stated in the colored boxes. Within a given PDX model, mice were to be randomized to the arms indicated, where the line color reflects the corresponding study goal. The first study goal used three drug arms to test efficacy of Drug A compared to carboplatin (goal 1, red). The second study goal used four drug arms to test efficacy of Drug A as a single agent and in combination with paclitaxel versus paclitaxel alone (goal 2, blue). The third study goal used four drug arms to test Drug A in combination with carboplatin (goal 3, green). The three studies called for 8 mice per arm in each of 11 arms, or 88 mice per PDX model. After discussion with the study team, we proposed a single six-arm study to be performed within each PDX model, with all three study goals utilizing the same control and Drug A arms, using 8 mice/arm * 6 drug arms * = 48 mice per PDX model. This represented a 45% reduction in mice required. Each study was to be performed in multiple PDX models, randomly drawn from platinum sensitive or platinum resistant phenotypes, so that effects could be compared between the two phenotypes.

Employing these study designs can help minimize the use of resources. For example, when an investigator proposed a series of three sequential studies in multiple PDX models with the goal of assessing the efficacy of a novel agent (Drug A) alone and in combination therapy (Fig. 5), the sequential studies would have used 8 mice per arm in each of 11 arms, or 88 mice, per PDX model. After discussion with the study team, we proposed a six-arm study to be performed within in each PDX model for a total of 48 mice per PDX model, resulting in a 45% reduction in mice. In addition, the multiple PDX models were randomly drawn from models known to be either platinum sensitive or platinum resistant so that effects could be compared between these two phenotypes. Thus, the entire multi-PDX experiment utilized a split plot design structure to randomly select sensitive/resistant PDX models, with mice randomly allocated to the six arms within PDX, with sensitive/resistant PDX models balanced over time^35,36^. In practice, since implementing the study in the laboratory one PDX model at a time was logistically feasible, PDX model tumors could be selected and reestablished from their frozen state over the course of the experiment to maintain consistent staffing and daily workload in the laboratory; and our data management system provided the laboratory personnel the ability to manage the treatment and measurement workload. A multi-arm protocol for each PDX model and a pre-determined randomization schedule was generated by the statistics team to assign treatment arms for individual mice when mouse tumors met criteria for therapy. While a full discussion of these design concepts is out of the scope of this manuscript, further reading for biologists regarding such experimental designs and how to implement them can be found in the following references^35,36^, and for the statistician regarding implementation and analytical code in this reference^17^.

The process of determining sample size requires balancing the desired statistical power to determine meaningful changes in light of the random variation present without being wasteful of time, money and animal resources^35,37–39^. The results herein provide guidance regarding sample size for the primary hypothesis test in ovarian PDX models. The results herein demonstrate that the common n = 10/arm may not provide sufficient power for some PDX models with larger variances. Alternatively, the secondary hypotheses comparing active arms to diluent generally have a larger expected effect size, and researchers may find lower levels of power, say 70%, to be acceptable for these hypothesis tests, and so smaller sample sizes may suffice. Thus, a reasonable resource-saving strategy is to first determine the sample size for arms involved in the primary hypothesis test, and then allocate half the sample size to arms only involved in the secondary hypothesis tests. For example, using a PDX model with medium variance to test the Case Study primary hypothesis of chemotherapy + MK versus chemo, we may allocate n = 10 per arm to the chemotherapy and chemotherapy + MK arms and n = 5/arm to the MK and diluent arms.

The present report has several strengths. Our data reflect experience with > 45 PDX models across several experiments to date. The mixed effects models use the full data available, not throwing any data away, and avoid discordant results that may be generated by performing tests at each time point. Thus, they simultaneously maximize power and minimize false discovery. The fitted models allow estimation of tumor doubling time, either from model parameters or from portions of the fitted curve in the case of curved trajectories. The data entry system has greatly improved efficiencies in the laboratory, allowing technicians to maintain more PDX models and perform more experiments with the same number of personnel. It has also greatly reduced the amount of time required to perform data cleaning and statistical analysis. This modeling strategy can accommodate change points, nonparametric estimates of the mean functional form (e.g., splines), or mathematical growth models other than exponential, all of which may be useful in studies with an observation phase in which mice are observed for recurrence after the end of treatment^7,30,31^.

Our report also has limitations. Implementation requires advanced statistical training and advanced software but results in improved efficiency and unified inference. The existence of collaborative teams that include both subject matter and statistical experts enables use of these methods by anyone in the scientific community. While our goal herein was to demonstrate application of linear mixed effects models to analyze tumor growth data in a single PDX model, it should be noted that analysis within each PDX model has inference space limited to that specific PDX model. This is appropriate for the clinical trial described, but too limited if the goal is to infer to the general ovarian PDX model population. The conclusions herein regarding between- and within-mouse variation and power and sample size calculations may not hold for PDX models from different tumor types or other modalities of tumor measurement (e.g., bioluminescence or serum biomarkers), and are likely most applicable to solid tumors. However, the general analytical and model fit assessment strategy we have outlined is applicable to any repeated measures setting.

## Methods

### Statistical modeling framework and design

Linear mixed effects regression models were used to compare growth trajectories between drug treatment arms^8,17,22,40^. Tumor burden measurements were assumed to be multiplicative on the raw scale and so additive on the natural log scale, and the overall tumor growth pattern was assumed to follow a smooth trajectory. The response variable was *ln*(*A*_*t*_) where *A*_*t*_ is the tumor area at time *t*. Measurements below the limit of detection were set to the limit of detection of 0.1 cm^2^ so that the natural log was defined for all data points. This limit was determined by SJW based on work in his PDX laboratory to validate the area measurements (personal communication). The between or among mouse covariate was treatment arm, coded as indicator variables. The within mouse covariate was time (the repeated factor) in days, ranging from t = 0 (baseline) to 28. Analyses were performed separately for each PDX model using the difference parameterization with diluent serving as the reference. Thus, treatment arm specific parameter estimates represent difference from diluent.

### Mean model

The independent variables day (centered), treatment arm indicator, and the 2-way interaction were included as fixed effects in the mean model. Day was centered by subtracting half of the length of the study from each time value, i.e., $t - \frac{28}{2}, t = \left( {0,4,8,12,16,20,24,28} \right)$, mapping the time values to $t_{c} = \left( { - 14, - 10, - 6, - 2,2,6,10,14} \right)$. With day centered, the y-intercept is interpreted as the average ln(A_t_) at the midpoint of the study (rather than at day 0) and can be thought of as an approximation to the area under the curve^41^. The slope parameter provides an estimate of the net growth and death rates^8,19^. Tumor area doubling (or halving) times can be estimated from a statistical model that is linear in time (straight) using [ln(2)/growth rate], where the growth (or regression) rate is the estimated slope for the treatment arm of interest^18,23,24,27^; if desired this could be converted to the volume scale^25^. Polynomial terms were added for PDX models with curved growth trajectories. With these more complicated models, the growth (or regression) rate varies over time, so there is not a single formula for doubling time, but plots or estimates of the fitted regression model can be used to generate these estimates^23^. The mean model functional form for each PDX model was chosen based on consideration of the following: (i) plots of overlaid observed and predicted mean model plots, (ii) residual plots, and (iii) ML AIC and BIC from models assuming independence^15^. Mouse-specific predicted trajectories were computed as the fixed effects solution plus the empirical best linear unbiased predictor. Mean values in figures were population average estimates from these models, scaled to the arm specific baseline estimate^27^, i.e., [(estimate at day t)/(estimate at baseline)]. 95% confidence intervals were indicted via shading.

### Variance/covariance structure

The following covariance structures were evaluated: (CS), sp(pow), Toep, UN, sp(pow) + RE, and (RE) with random between subject intercept and slope. In the sp(pow) model fits, the autoregressive parameter was verified to be not near the parameter space boundaries of 0 or 1. In the SAS PROC MIXED procedure, within subject variation is specified in the REPEATED statement (CS, Toep, sp(pow), UN), while between subject variation is specified in the RANDOM statement (RE). Since the covariance is a function of time for some structures, the time variable in the REPEATED or RANDOM statements was *not* centered. Variance parameters were estimated via REML for choosing variance structure and making inferences while holding the mean model fixed to allow proper comparison of model information criterion statistics. Correlation structures were evaluated via: (i) semivariogram plots, (ii) REML AIC and BIC criteria, and (iii) estimated covariance matrices^11,16,17^. The UN structure was also approximated by computing a covariance matrix from the residuals of a ML independence model, which is especially useful if the limited amount of available data does not support estimation of the UN structure.

### Hypothesis testing

A test of equal intercepts addresses the question of whether the average *ln*(*A*_*t*_) is different in the middle of the study since the day variable is centered. A test of equal slopes addresses the question of whether the rate of growth/regression is different across the range of the study. A tumor that responds immediately in one treatment arm and has a delayed but similar response rate in a different arm may have similar slopes. However, the growth trajectories look different by eye and thus are judged clinically different, indicating that differences in one or the other or both parameters are meaningful. Thus, to address the primary goal of comparing average tumor growth trajectories between treatment arms, a multiple degree of freedom hypothesis test of coincident curves incorporating both mean and slope parameters that differ by treatment arm was performed via contrast statements. Denominator degrees of freedom were estimated via the Kenward-Rogers approximation^17^. Contrast statements were used to implement Wald F-tests, as these are appropriate with REML estimation in small sample sizes.

### Power calculations

An RShiny application was developed to perform power calculations via simulation for a 28-day experiment with centered times $t_{c} = \left( { - 14, - 10, - 6, - 2,2,6,10,14} \right)$. The following straight trajectories were used to simulate 500 datasets $\ln \left( {A_{t} } \right) = - 0.1115 - 0.0079t_{c}$, $\ln \left( {A_{t} } \right) = - 0.2231 - 0.0159t_{c}$, $\ln \left( {A_{t} } \right) = - 0.3669 - 0.0262t_{c}$, $\ln \left( {A_{t} } \right) = - 0.6019 - 0.0429t_{c}$, and $\ln \left( {A_{t} } \right) = - 0.3465 - 0.0247t_{c}$ assuming sp(pow) covariance with parameter 0.98. Per arm mouse sample size ranged from 5 to 20, and three residual variance values representing approximate Q25, Q50 and Q75 percentiles of the NCT02312245 clinical trial PDX model *ln*(*A*_*t*_) residual variance distribution. All arms start with day 0 (*t*_*c*_ = − 14) 1cm^2^ tumor area. For each hypothesis test, power is the proportion of times out of 500 the p-value was less than 0.05.

### Study conduct

A SAS based web data entry system has been developed to facilitate tracking of PDX models and performing these experiments. Randomization of mice to study arm is built into the system according to a balanced block design (incorporated after the Case Study shown here), where block size is equal to the number of study arms. The system sends automatic daily to-do lists via email to laboratory personnel to inform them which mice need procedures or treatments that day. Tumor area measurements are entered by laboratory personnel directly into the system as they perform the measurements. Data are easily extracted via SAS and/or R functions at the completion of an experiment for analysis. Analyses were performed via R^42^ and SAS software (copyright 2016, SAS Institute Inc. SAS and all other SAS Institute Inc. product or service names are registered trademarks or trademarks of SAS Institute Inc., Cary, NC, USA).

## Supplementary Information

Supplementary Information

## Data Availability

The datasets generated during and/or analyzed during the current study are available from the corresponding author on reasonable request.

## Abbreviations

PDX: Patient derived xenograft
PARPi: Polymerase inhibitor
ANOVA: Analysis of variance
MK: MK-4827, niraparib
IACUC: Institutional Animal Care and Use Committee
AIC: Akaike information criterion
BIC: Bayesian information criterion
ML: Maximum likelihood
REML: Restricted (or residual) maximum likelihood
UN: Unstructured
sp(pow): Spatial(power)
sp(pow) + RE: Spatial(power) plus random effect
Toep: Toeplitz
CS: Compound symmetric
RE: Random effects
HR: Homologous repair
HRD: Homologous repair deficient
AUC: Area under the curve
EBLUP: Empirical best linear unbiased predictor
