# Supplementary material for: Statistical analysis of comparative tumor growth repeated measures experiments in the ovarian cancer patient derived xenograft (PDX) setting
Source: Sci Rep. 2021 Apr 13;11:8076. doi: 10.1038/s41598-021-87470-x (PMC8044116; doi:10.1038/s41598-021-87470-x)
Supplement: Supplementary file 1 — Supplementary Information [file 41598_2021_87470_MOESM1_ESM.pdf]

Supplementary Information for: Statistical analysis of comparative tumor growth repeated measures experiments in the ovarian cancer patient derived xenograft (PDX) setting

Ann L. Oberg[1*], Ethan P. Heinzen[1], Xiaonan Hou[2], Mariam M. Al Hilli[3#,4§], Rachel M. Hurley[5#,6§], Andrea E. Wahner Hendrickson[2], Krista M. Goergen[1], Melissa C. Larson[1], Marc A. Becker[2#,7§], Jeanette E. Eckel-Passow[1], Matthew J. Maurer[1], Scott H. Kaufmann[5,8], Paul Haluska[2#,§9], S. John Weroha[2]

1. Division of Computational Biology, Department of Quantitative Health Sciences, Mayo Clinic, Rochester, MN, USA.
2. Division of Medical Oncology, Department of Oncology, Mayo Clinic, Rochester, MN, USA.
3. Division of Gynecologic Oncology, Department of Obstetrics & Gynecology, Mayo Clinic, Rochester, MN, USA.
4. Division of Subspecialty Care for Women's Health, Gynecologic Oncology, Women's Health Institute, Cleveland Clinic, Cleveland, OH, USA.
5. Department of Molecular Pharmacology & Experimental Therapeutics, Mayo Clinic, Rochester, MN, USA.
6. Department of Pediatrics, The Children's Hospital of Philadelphia, Philadelphia, PA, USA.
7. Blueprint Medicines, 45 Sidney St., Cambridge, MA, USA.
8. Division of Oncology Research, Department of Oncology, Mayo Clinic, Rochester, MN, USA.
9. Bristol-Meyers Squibb, 3401 Princeton Pike, Lawrenceville, NJ, USA.

*To whom correspondence should be addressed: Mayo Clinic, 200 First Street SW, Rochester MN, 55905, USA Tel: 1-507-538-1556; Fax: 1-507-266-2477; Email: oberg.ann@mayo.edu
[#] Site where work was performed
[§] Current address

Supplemental Figure 1. Jitter plots showing baseline tumor area (cm²) by drug arm for each PDX model. Horizontal lines indicate the median.

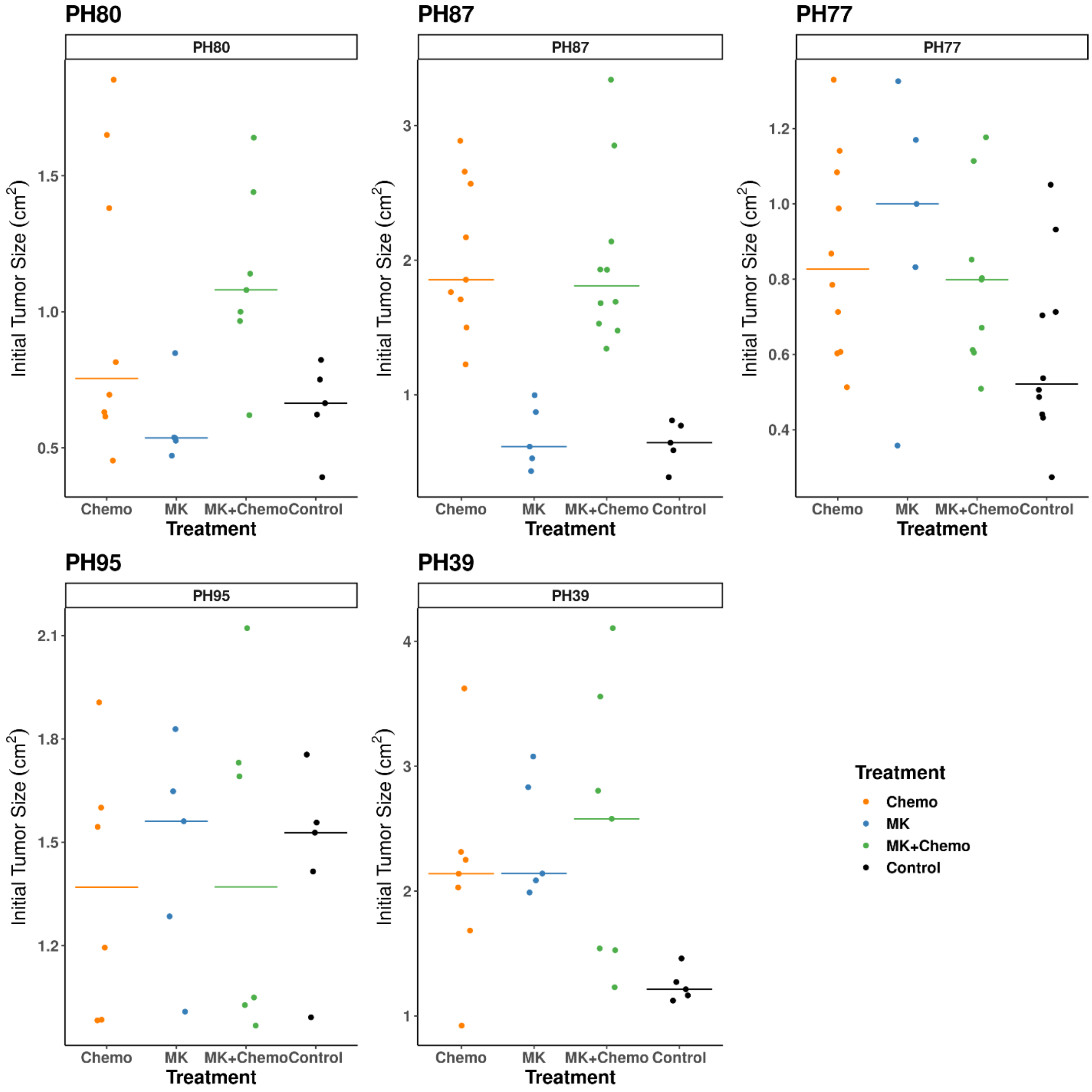

Supplemental Figure 2. Profile plot (per-mouse trajectories) for all five PDX models on the raw measurement scale (cm²). Coloring indicates the following four drug arms: control (diluent), chemotherapy (carboplatin+paclitaxel), the PARPi niraparib (MK-4827; abbreviated MK herein), or chemotherapy plus MK (carboplatin+paclitaxel+MK). Lines connect observations from the same mouse provide a visual check of balance at baseline (day=0), a sense of whether growth trajectories are straight or curved (functional form), and the magnitude of between mouse variation and loss to follow up. Per arm sample sizes at day 0 for each PDX model are as follows: PH80: control n=5, chemo n=8, MK n = 5, MK+chemo n=7; PH87: control n=5, chemo n=9, MK n=5, MK+chemo n=7; PH77: control n=10, chemo n=10, MK n=5, MK+chemo n=9; PH95: control n=5, chemo n=6, MK n=5, MK+chemo n=6; PH39: control n=5, chemo n=8, MK n=5, MK+chemo n=7.

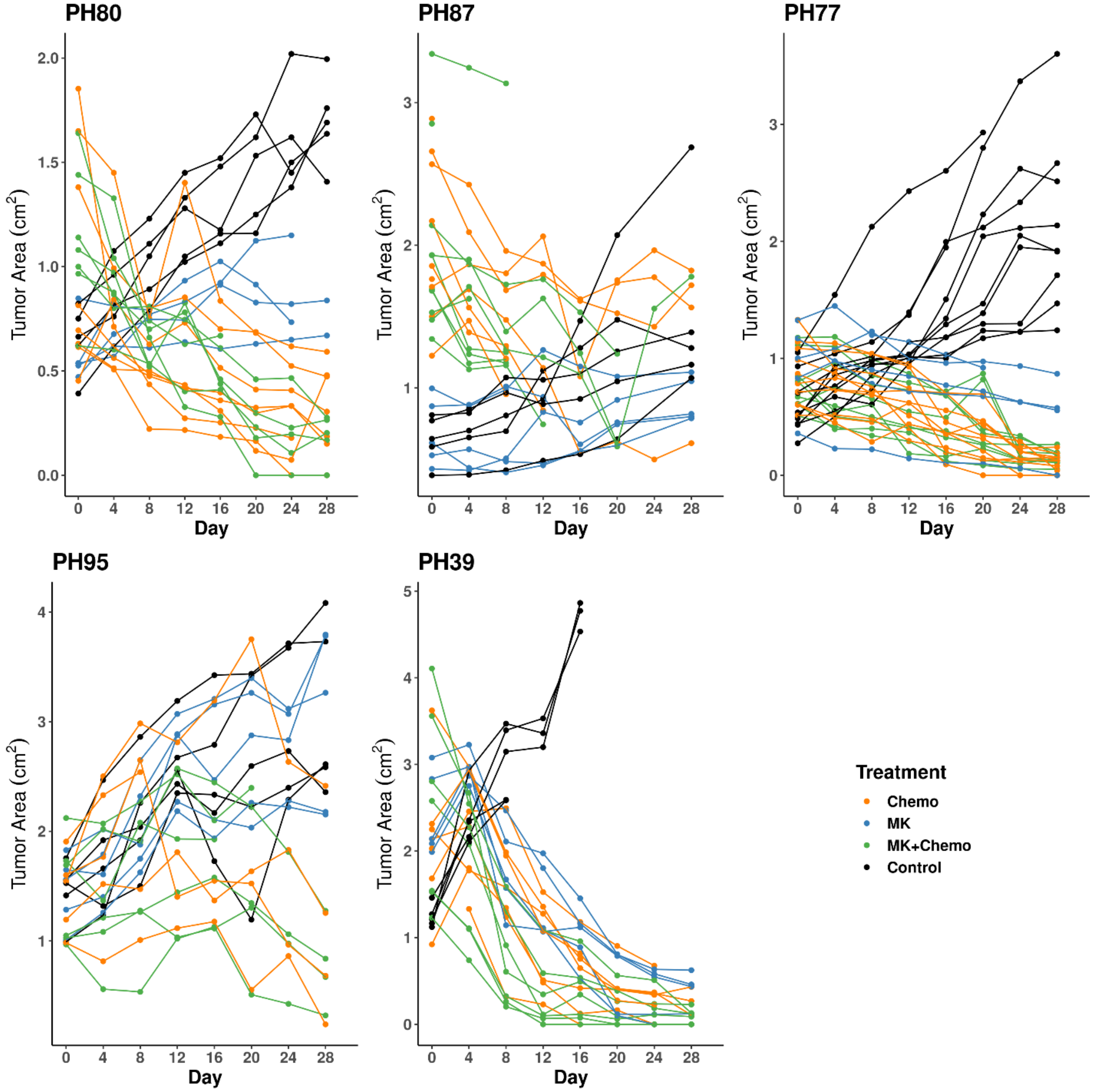

Supplemental Figure 3: ML Model Fits and Residuals

Contents

PH80 3

PH87 11

PH77 19

PH95 27

PH39 35

PH80

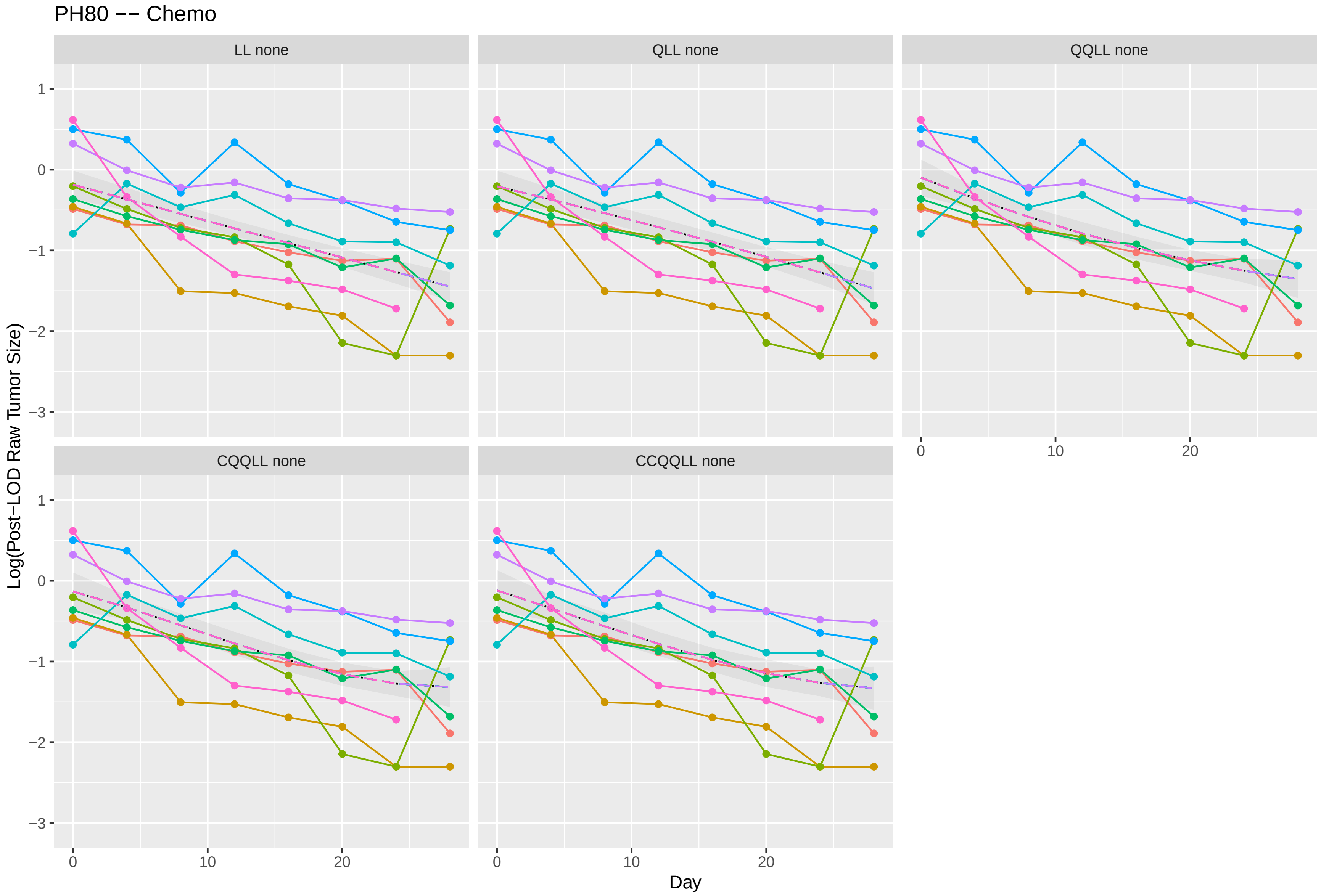

PH80 -- Chemo
LL none
QLL none
QQLL none
CQQLL none
CCQQLL none
Log(Post−LOD Raw Tumor Size)
Day
1
0
−1
−2
−3
0
10
20

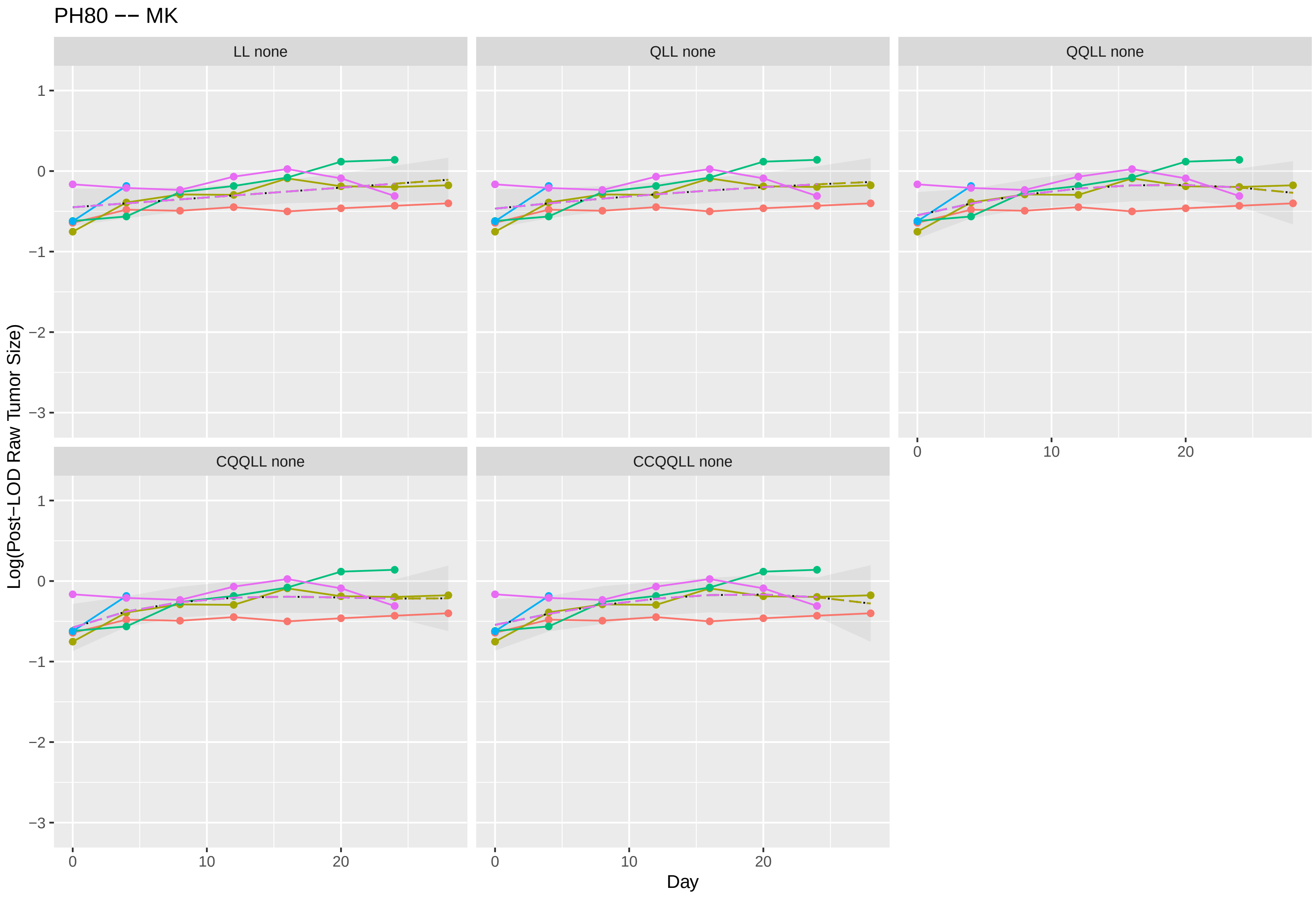
PH80 -- MK
LL none
QLL none
QQLL none
CQQLL none
CCQQLL none
Log(Post−LOD Raw Tumor Size)
Day
1
0
−1
−2
−3
0
10
20

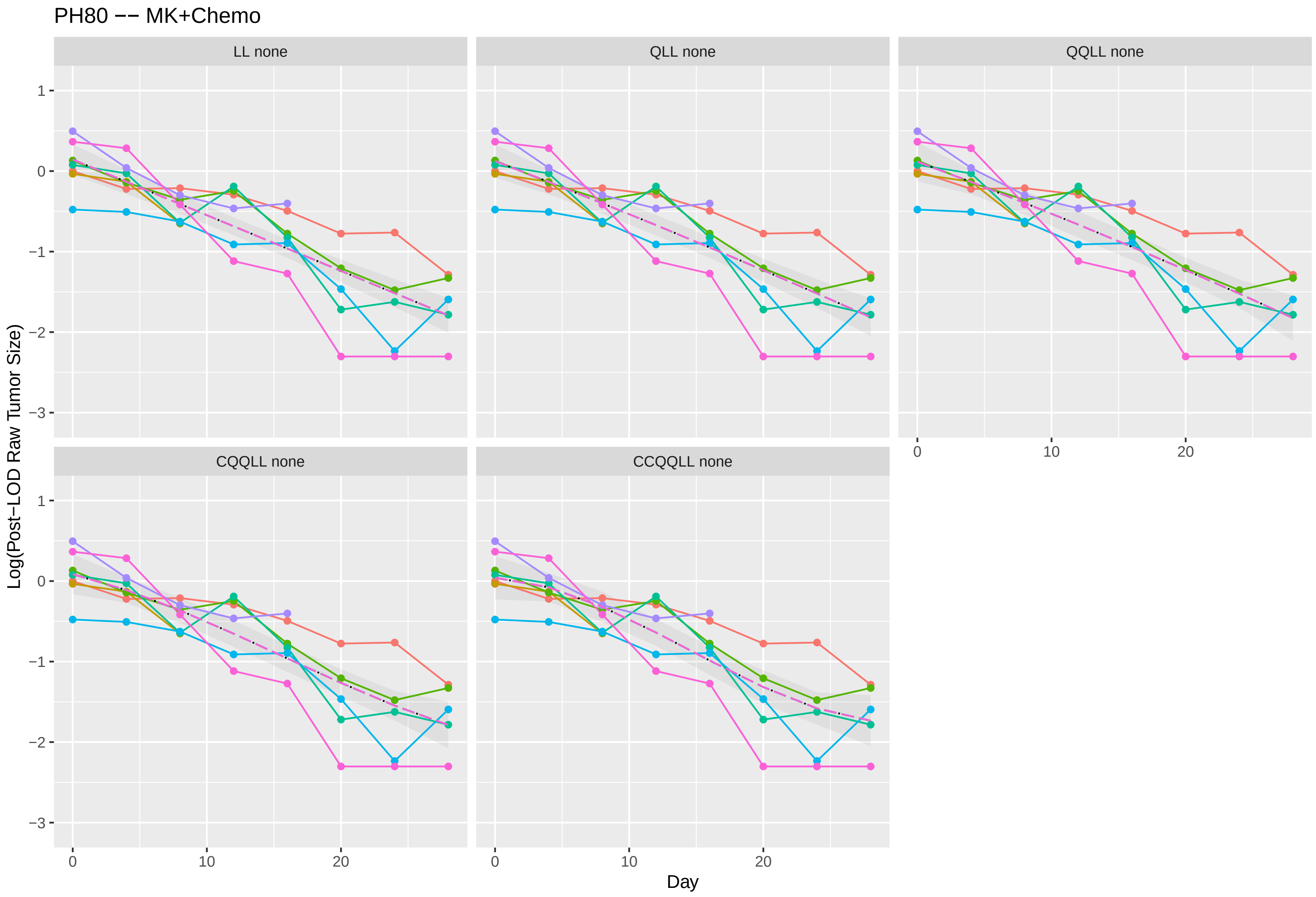
PH80 -- MK+Chemo
LL none
QLL none
QQLL none
CQQLL none
CCQQLL none
Log(Post-LOD Raw Tumor Size)
Day
1
0
−1
−2
−3
0
10
20

PH80 −− Control

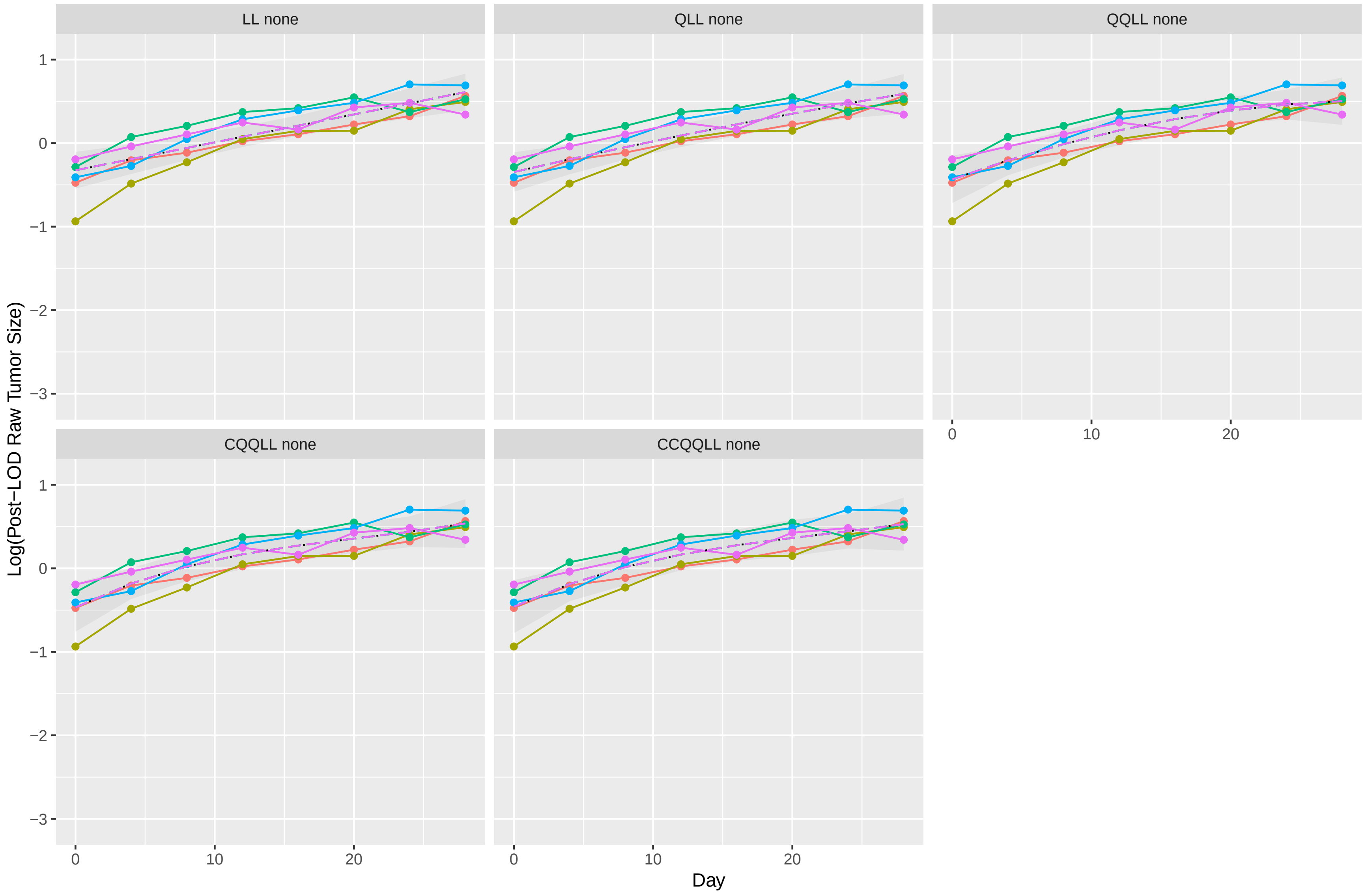

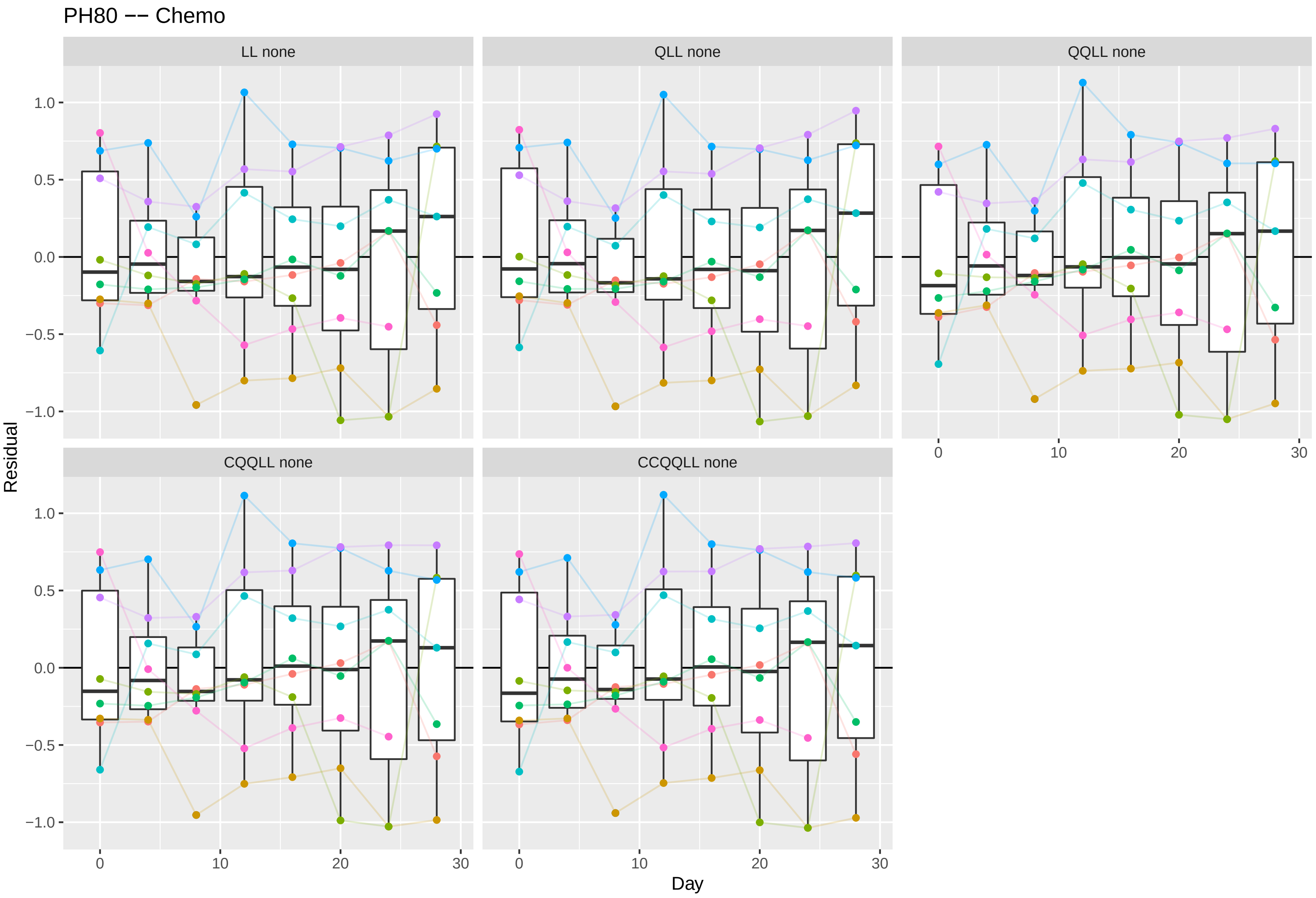
PH80 -- Chemo
LL none
QLL none
QQLL none
CQQLL none
CCQQLL none
Residual
Day
1.0
0.5
0.0
−0.5
−1.0
0
10
20
30

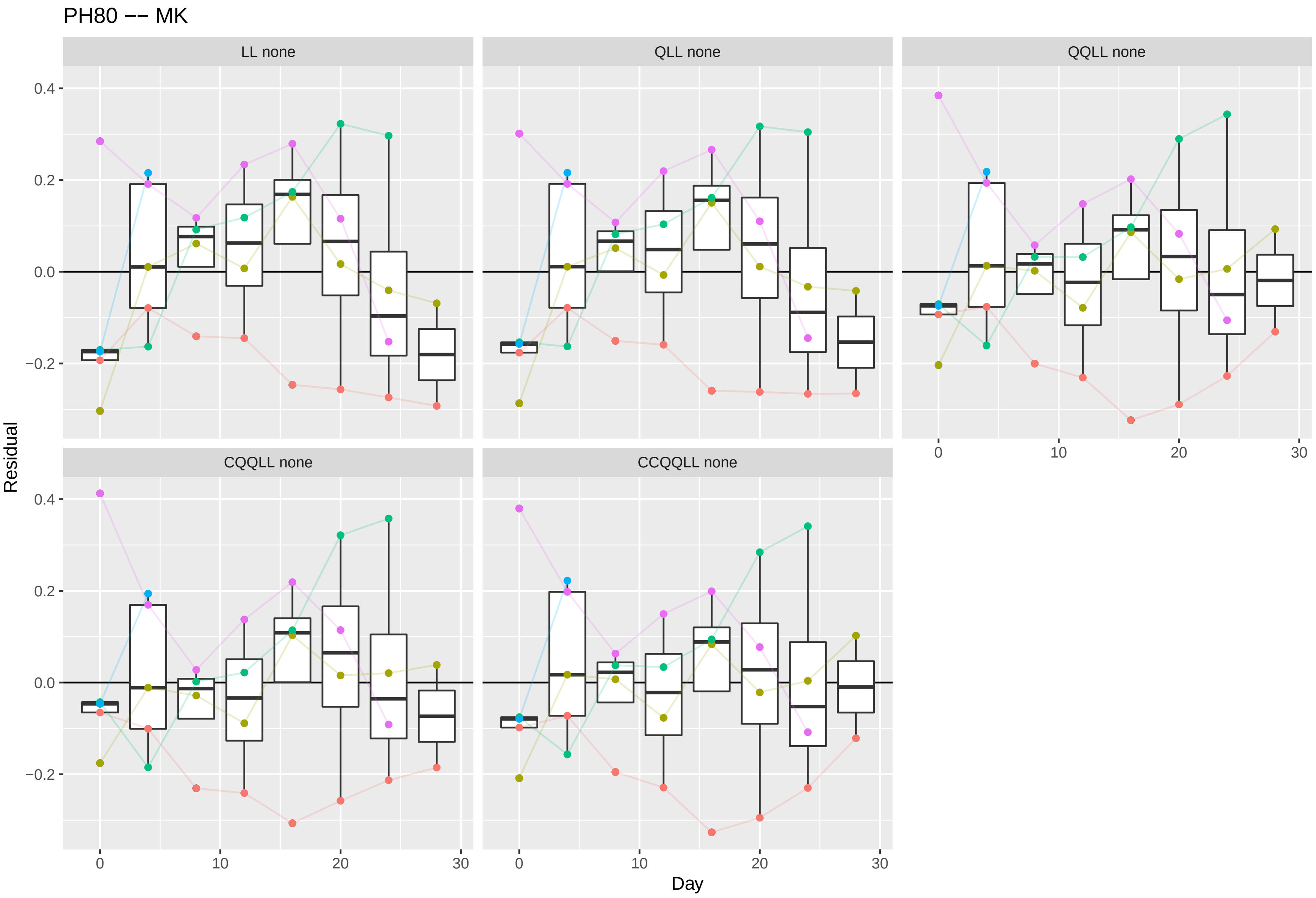
PH80 -- MK
LL none
QLL none
QQLL none
CQQLL none
CCQQLL none
Residual
Day
0.4
0.2
0.0
−0.2
0
10
20
30

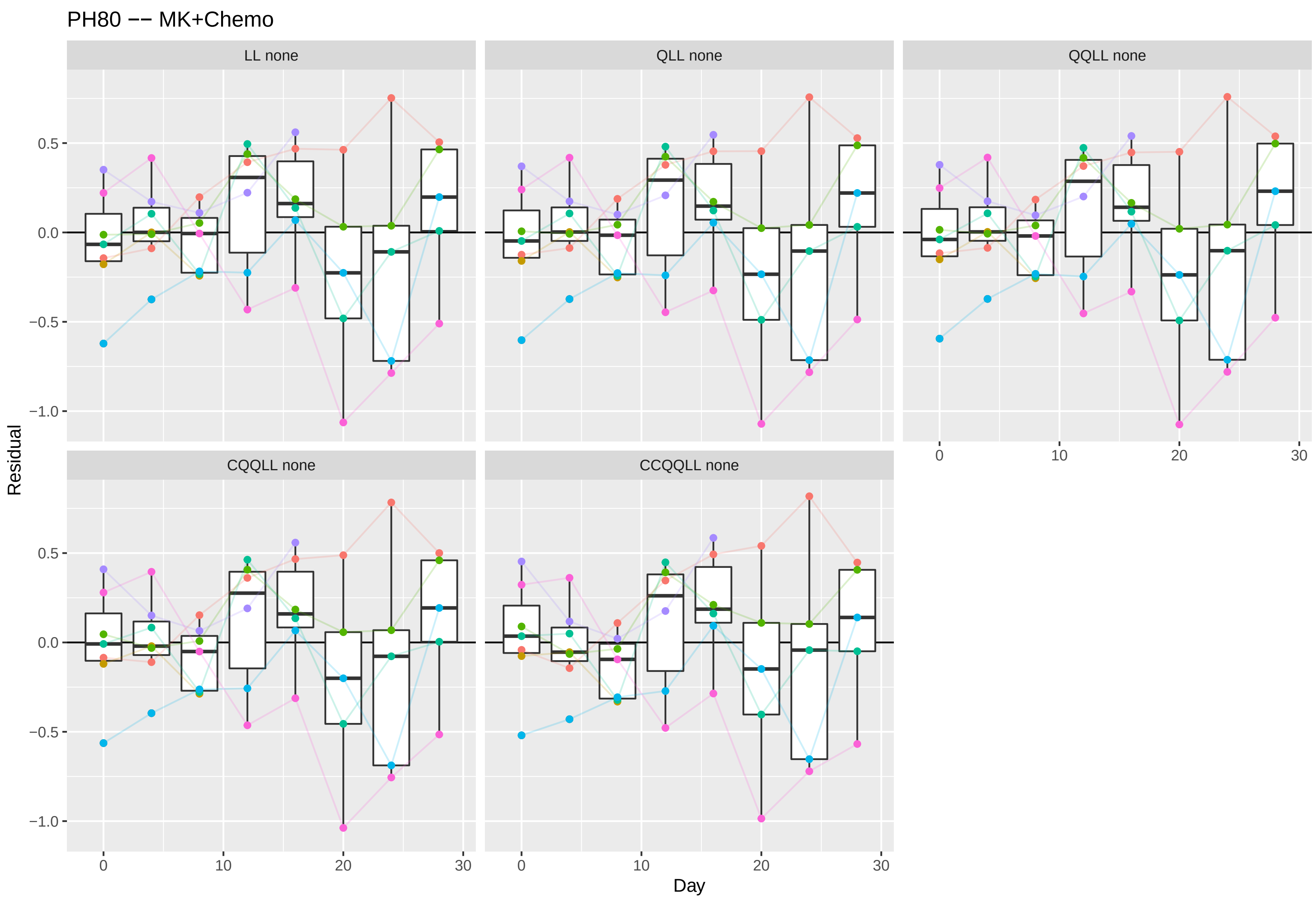
PH80 −− MK+Chemo
LL none
QLL none
QQLL none
CQQLL none
CCQQLL none
Residual
Day
0.5
0.0
−0.5
−1.0
0
10
20
30

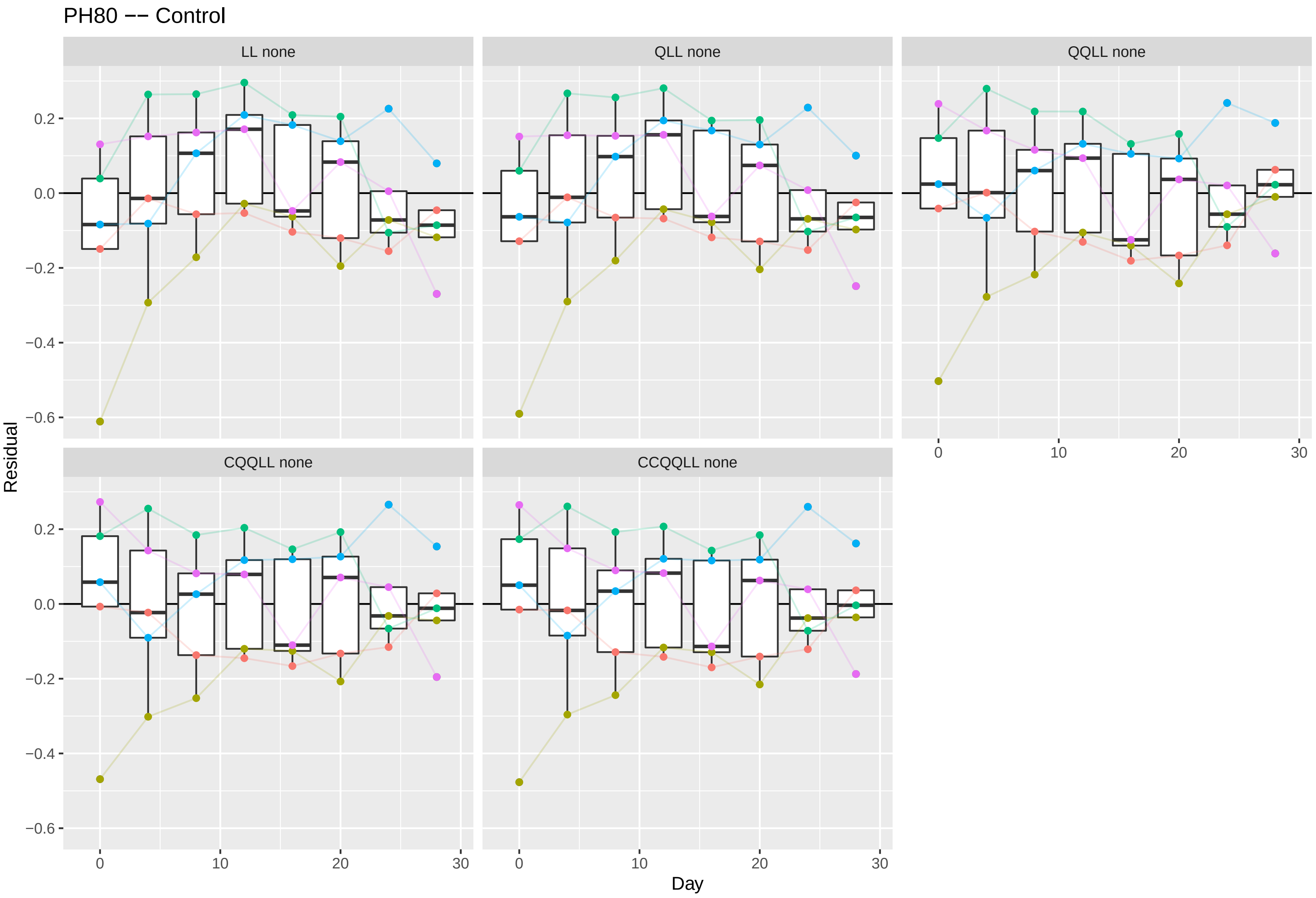
PH80 −− Control
LL none
QLL none
QQLL none
CQQLL none
CCQQLL none
Residual
Day
0.2
0.0
−0.2
−0.4
−0.6
0
10
20
30

PH87

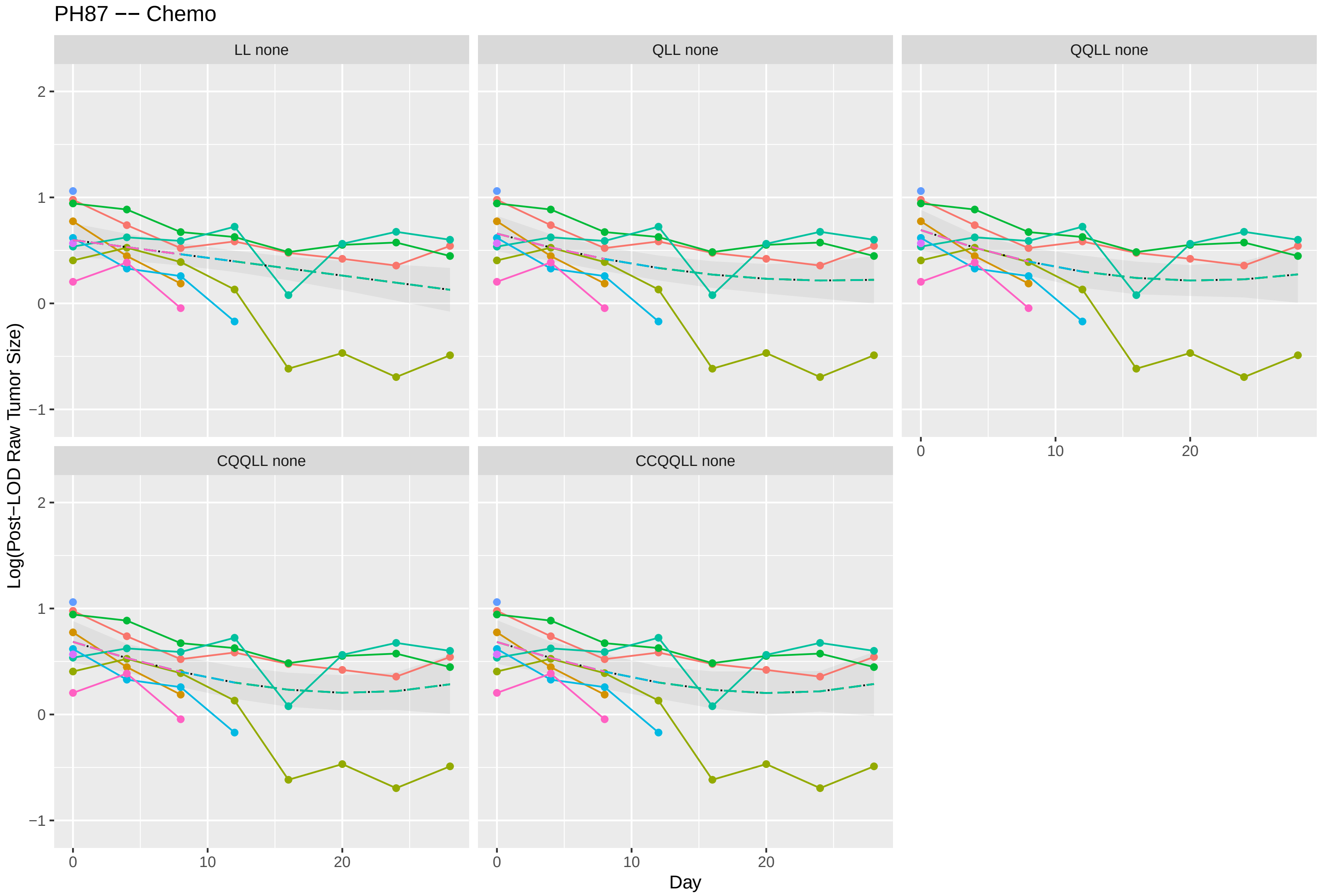

PH87 -- Chemo
LL none
QLL none
QQLL none
CQQLL none
CCQQLL none
Log(Post−LOD Raw Tumor Size)
Day
2
1
0
−1
0
10
20

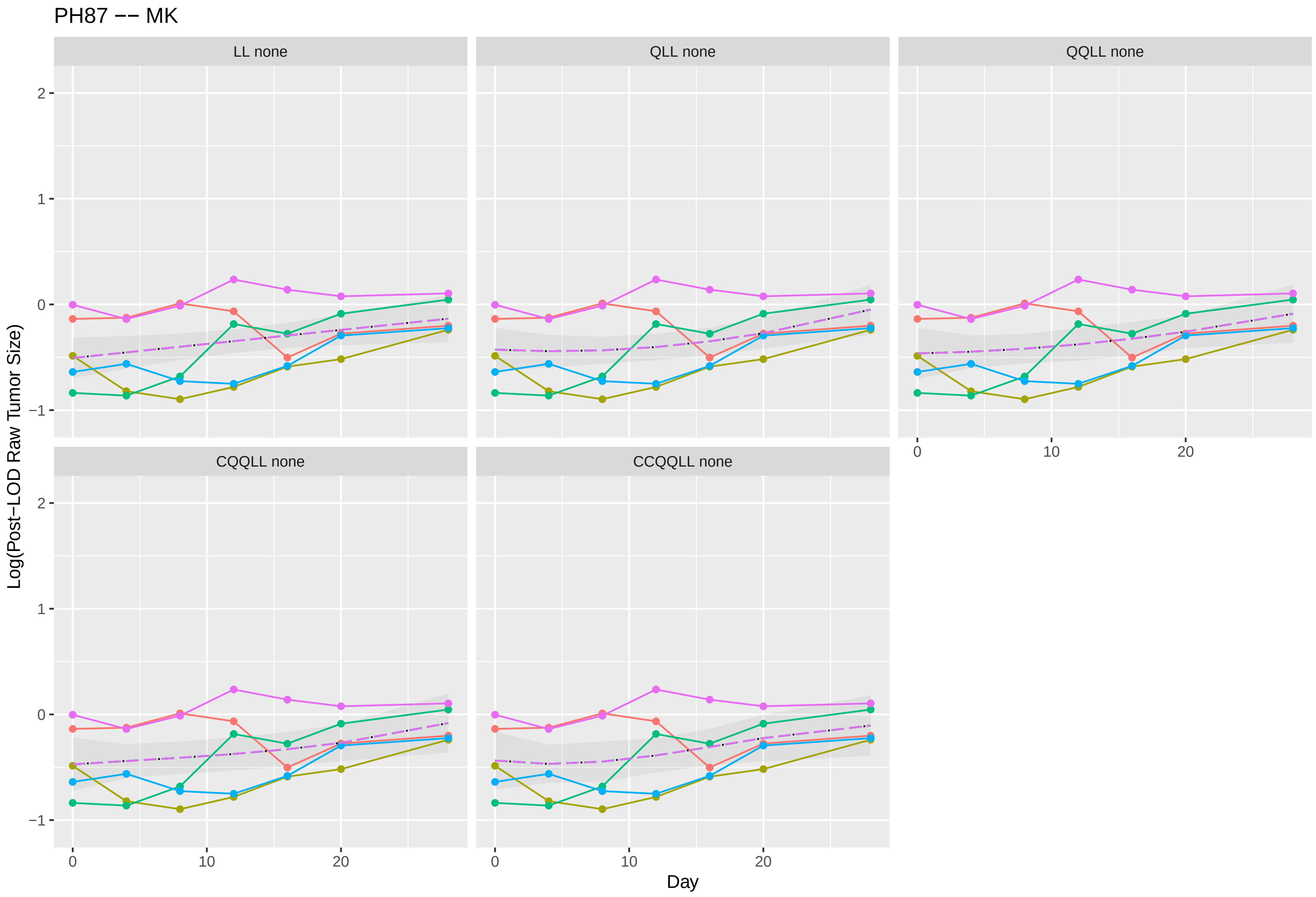
PH87 -- MK
LL none
QLL none
QQLL none
CQQLL none
CCQQLL none
Log(Post-LOD Raw Tumor Size)
Day
2
1
0
-1
0
10
20

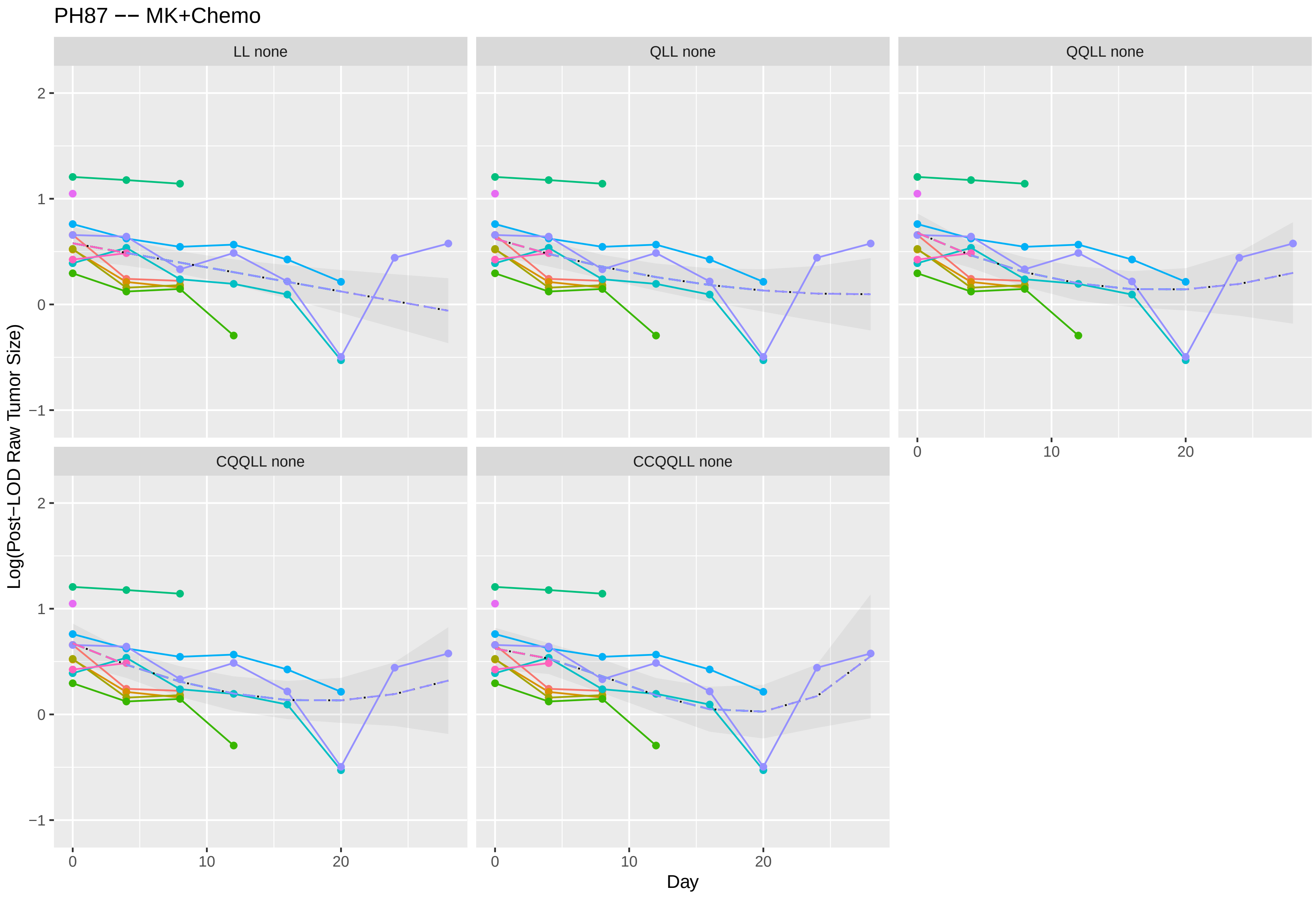

PH87 −− MK+Chemo
LL none
QLL none
QQLL none
CQQLL none
CCQQLL none
Log(Post−LOD Raw Tumor Size)
Day
2
1
0
−1
0
10
20

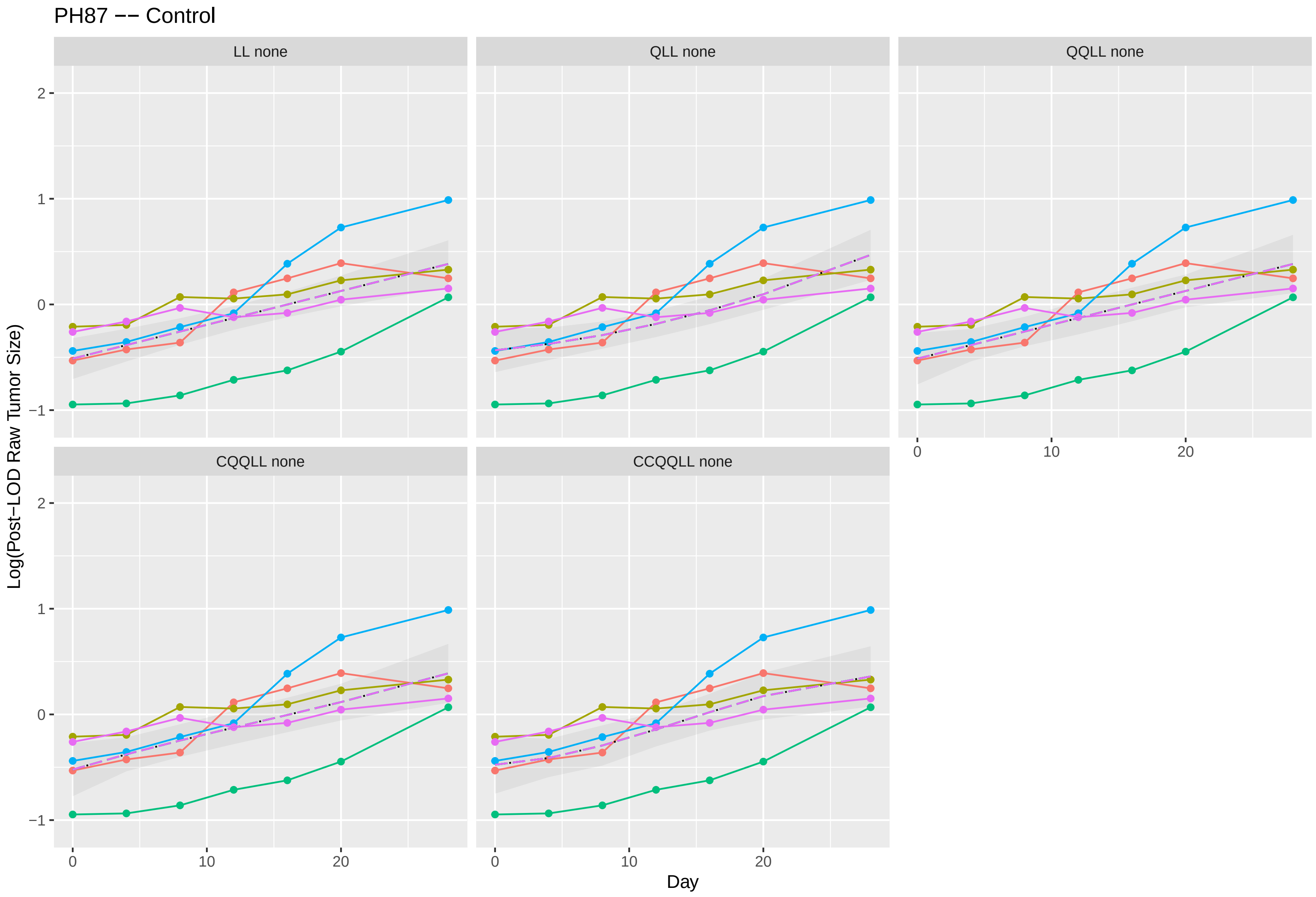
PH87 −− Control
LL none
QLL none
QQLL none
CQQLL none
CCQQLL none
Log(Post−LOD Raw Tumor Size)
Day
2
1
0
−1
0
10
20

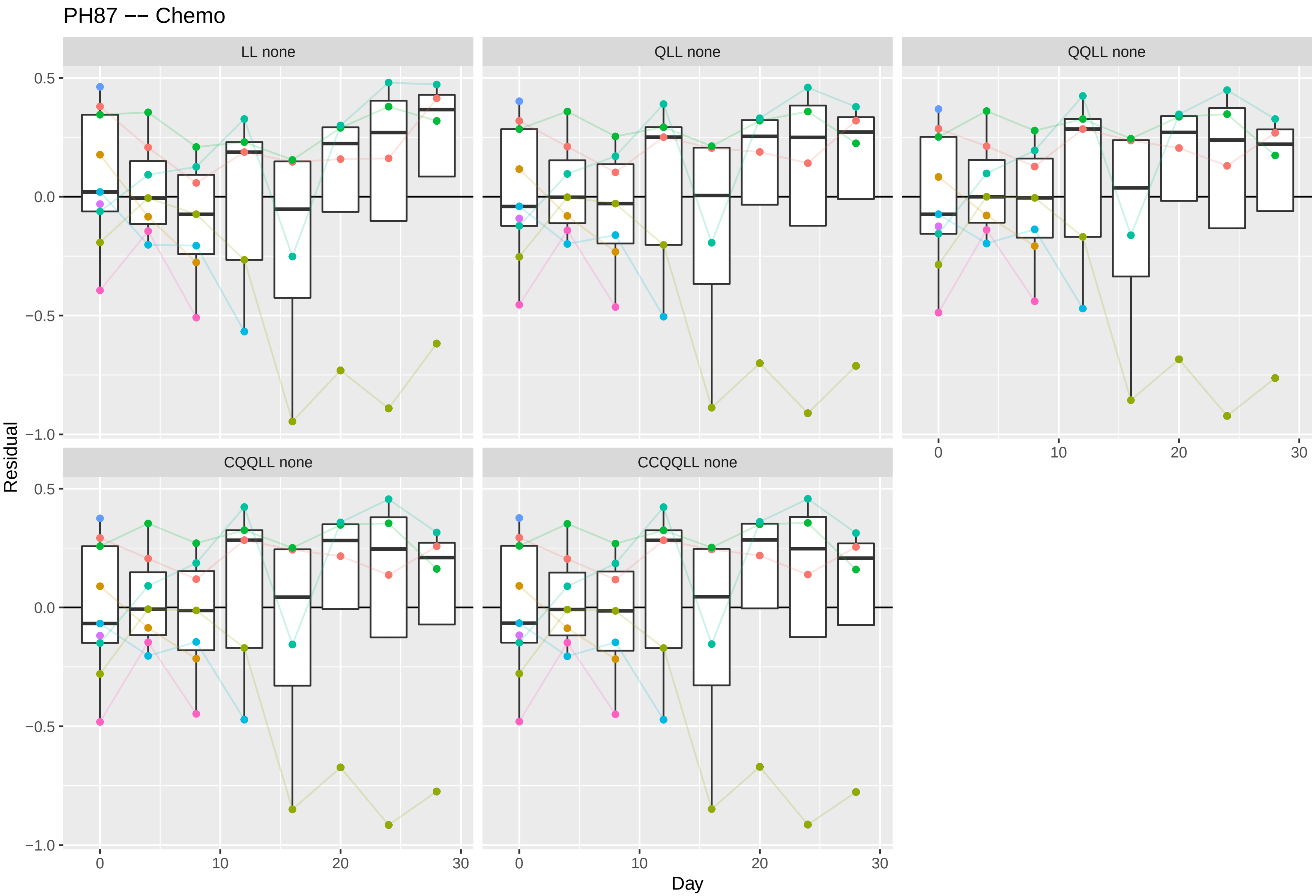
PH87 -- Chemo
LL none
QLL none
QQLL none
CQQLL none
CCQQLL none
Residual
Day
0.5
0.0
−0.5
−1.0
0
10
20
30

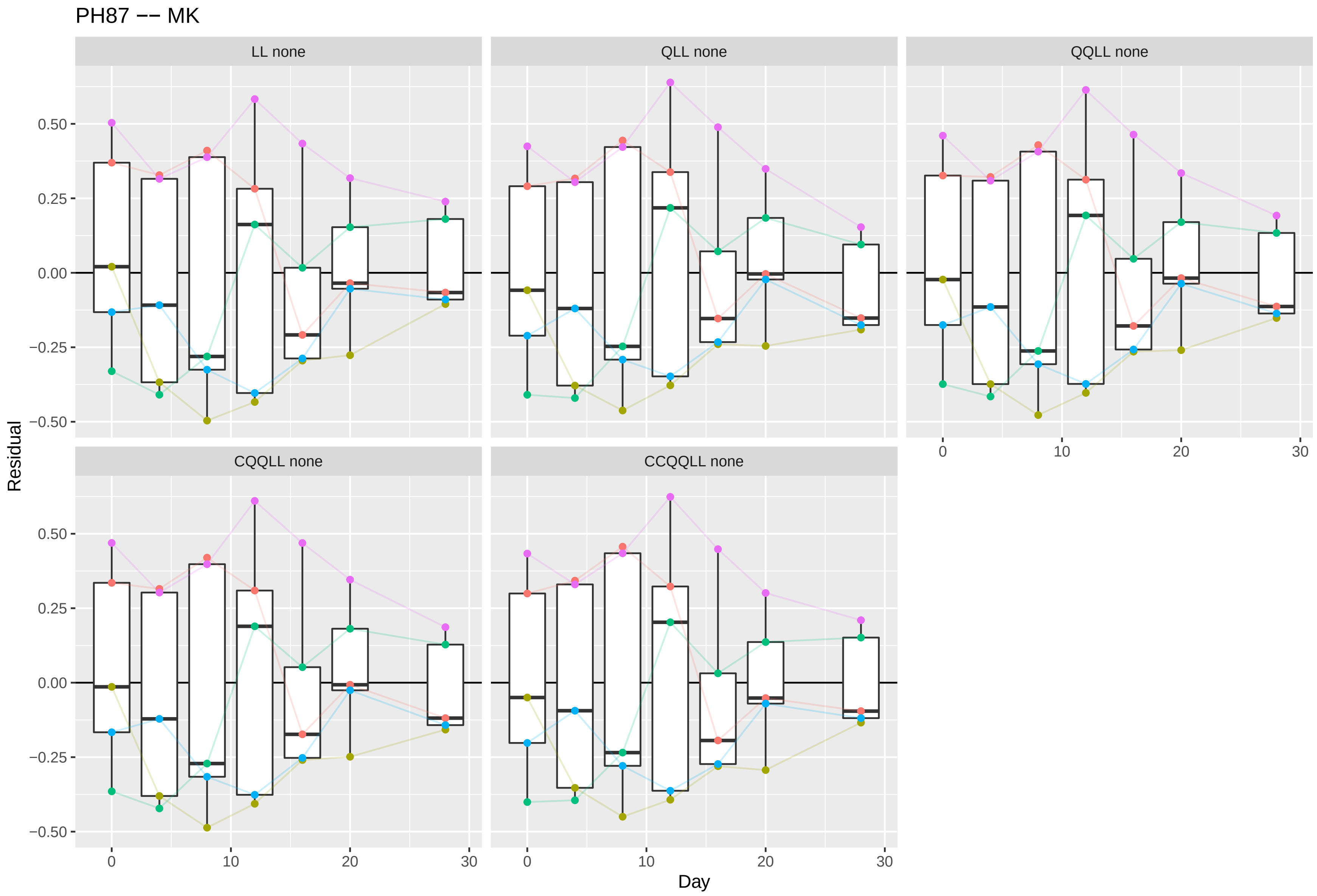
PH87 -- MK
LL none
QLL none
QQLL none
CQQLL none
CCQQLL none
Residual
Day
0.50
0.25
0.00
−0.25
−0.50
0
10
20
30

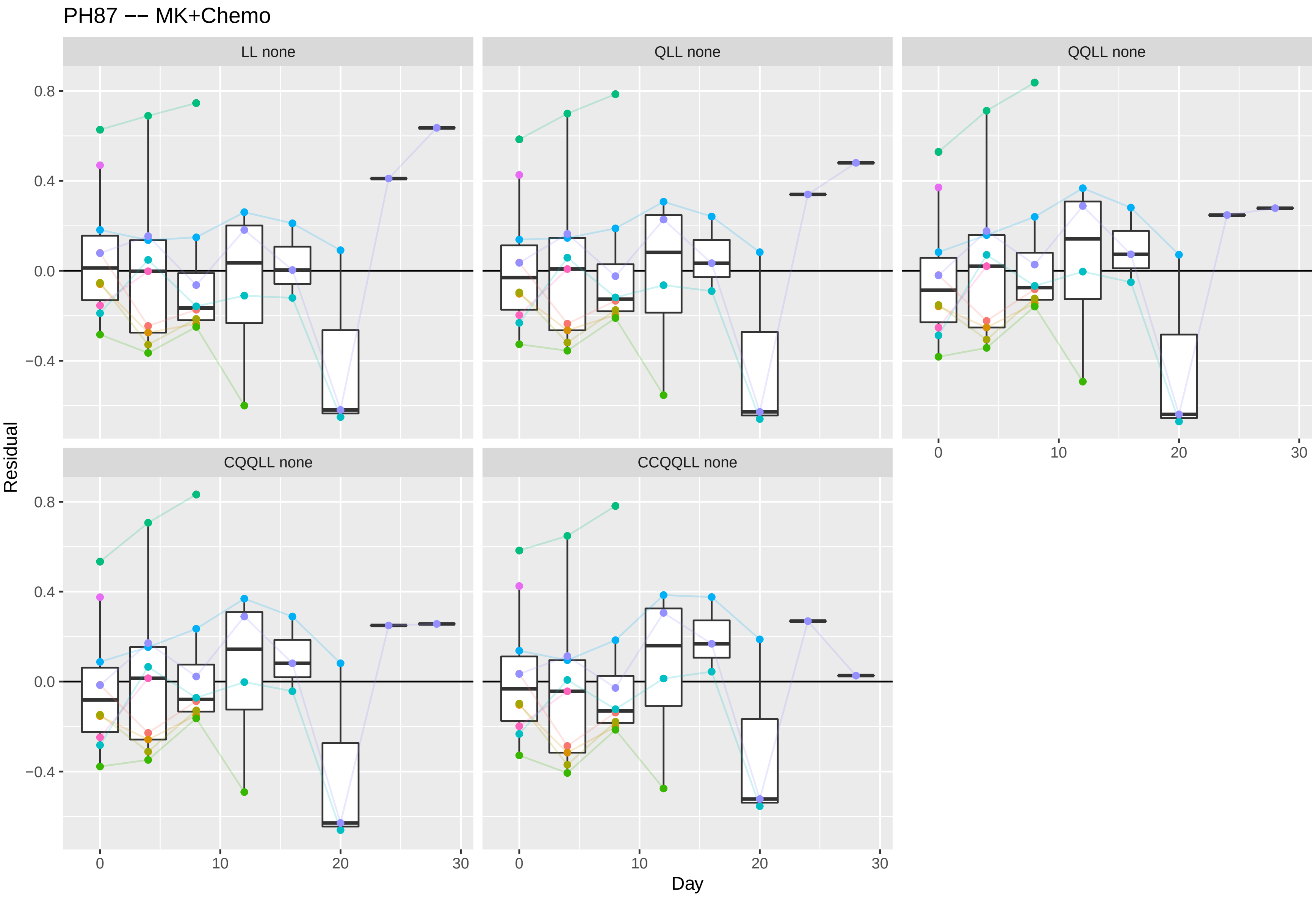

PH87 -- MK+Chemo
LL none
QLL none
QQLL none
CQQLL none
CCQQLL none
Residual
Day
0.8
0.4
0.0
−0.4
0
10
20
30

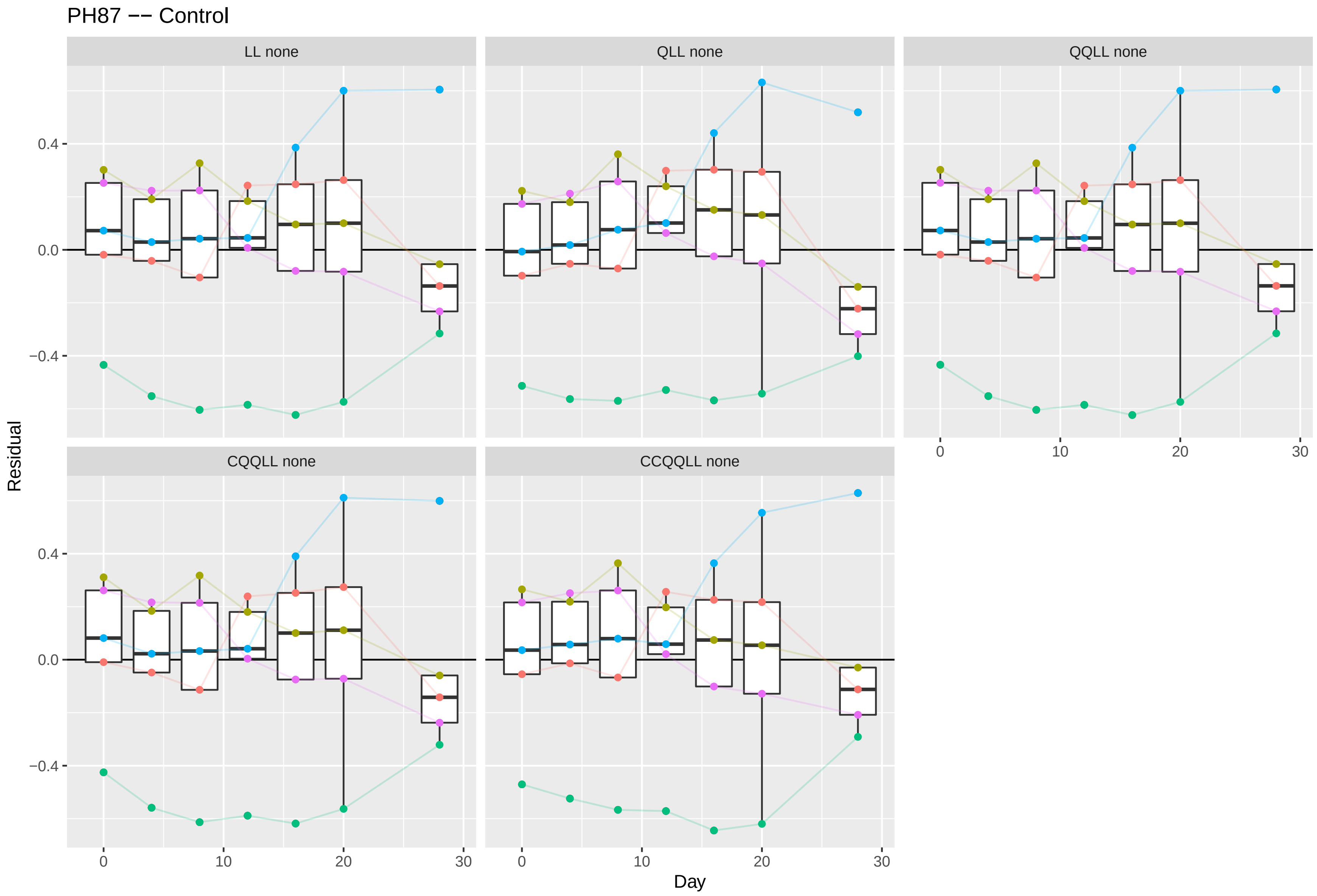
PH87 -- Control
LL none
QLL none
QQLL none
CQQLL none
CCQQLL none
Residual
0.4
0.0
−0.4
0
10
20
30
Day

PH77

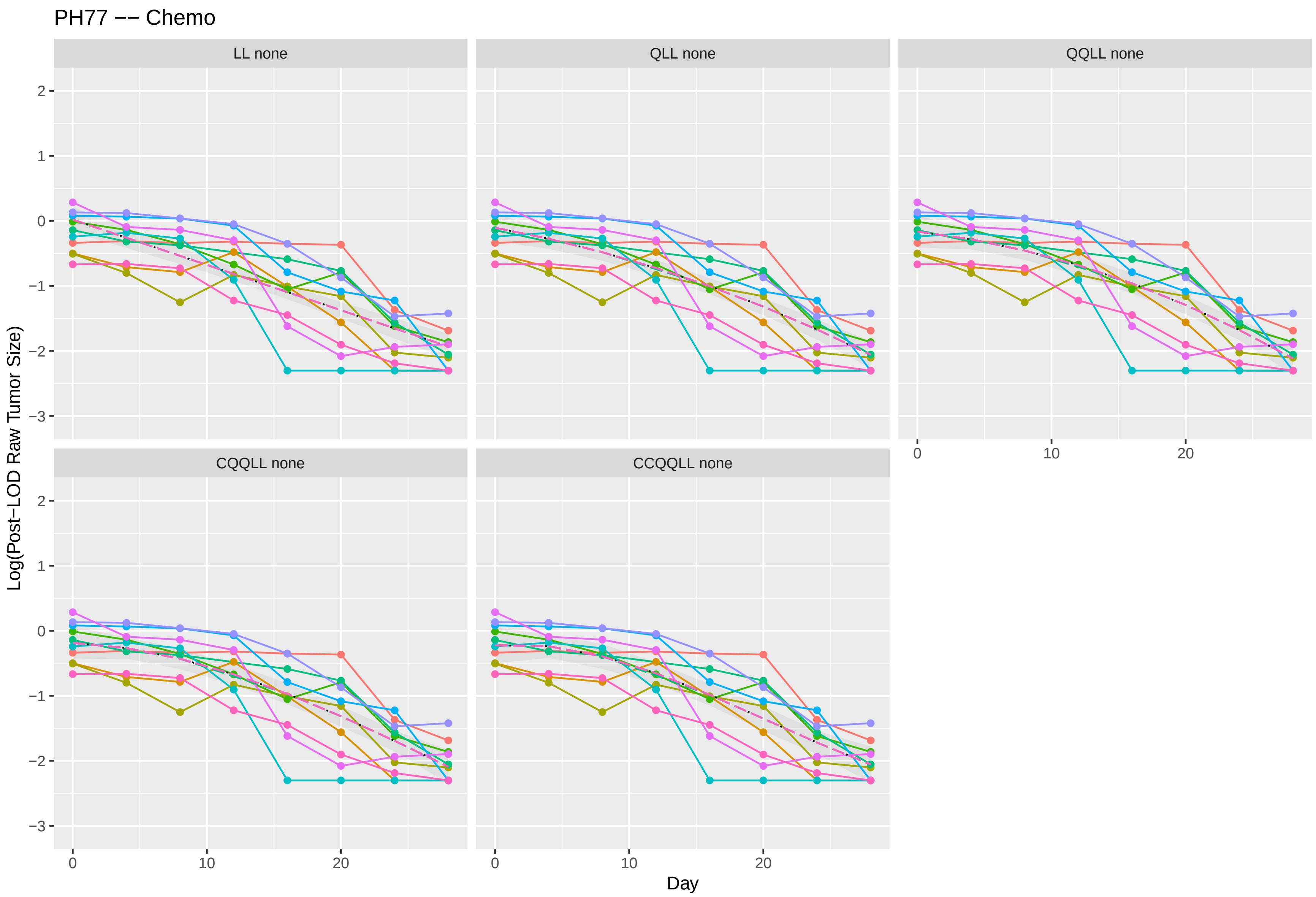

PH77 -- Chemo
LL none
QLL none
QQLL none
CQQLL none
CCQQLL none
Log(Post−LOD Raw Tumor Size)
Day
2
1
0
−1
−2
−3
0
10
20

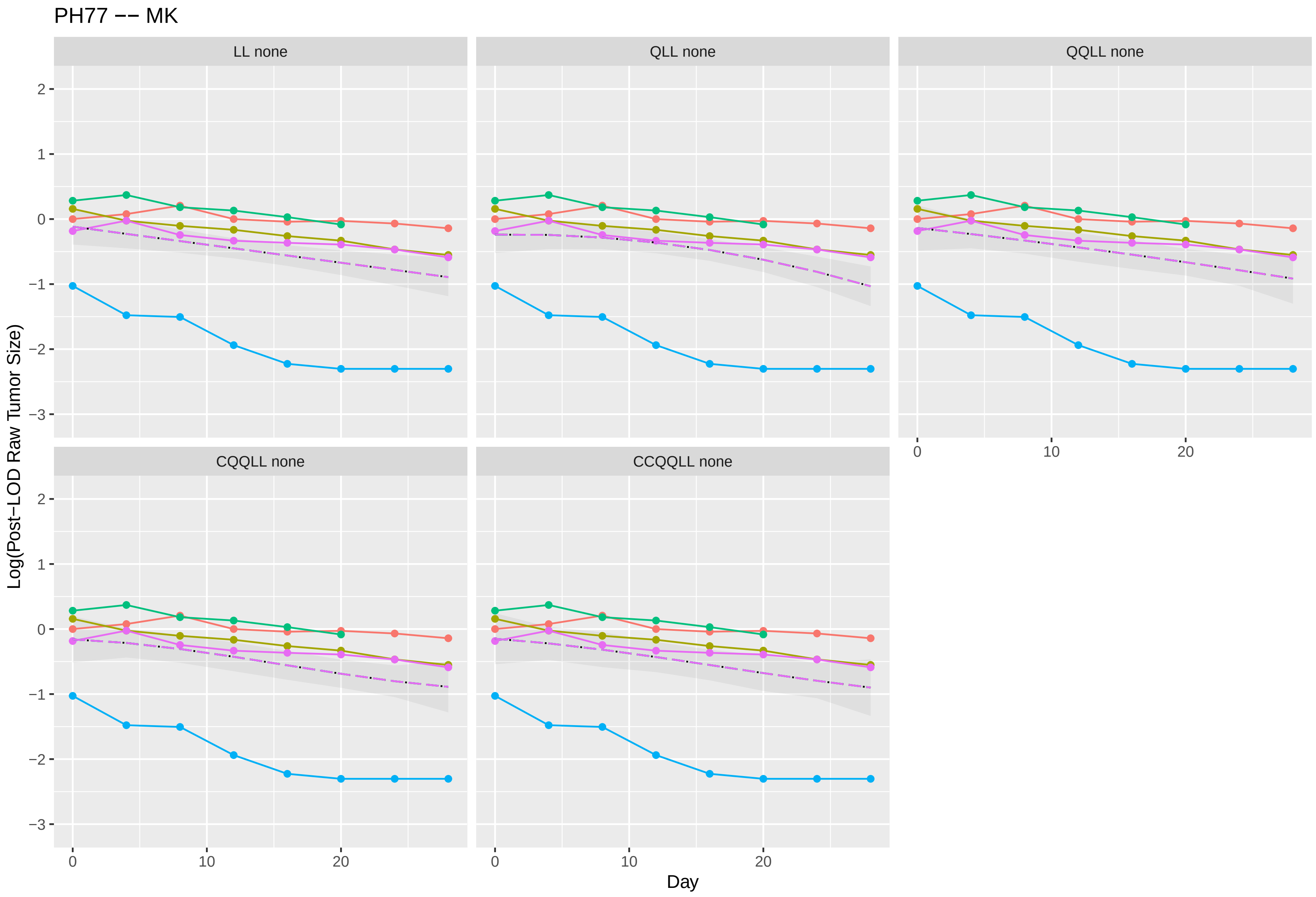
PH77 -- MK
LL none
QLL none
QQLL none
CQQLL none
CCQQLL none
Log(Post-LOD Raw Tumor Size)
Day
2
1
0
−1
−2
−3
0
10
20

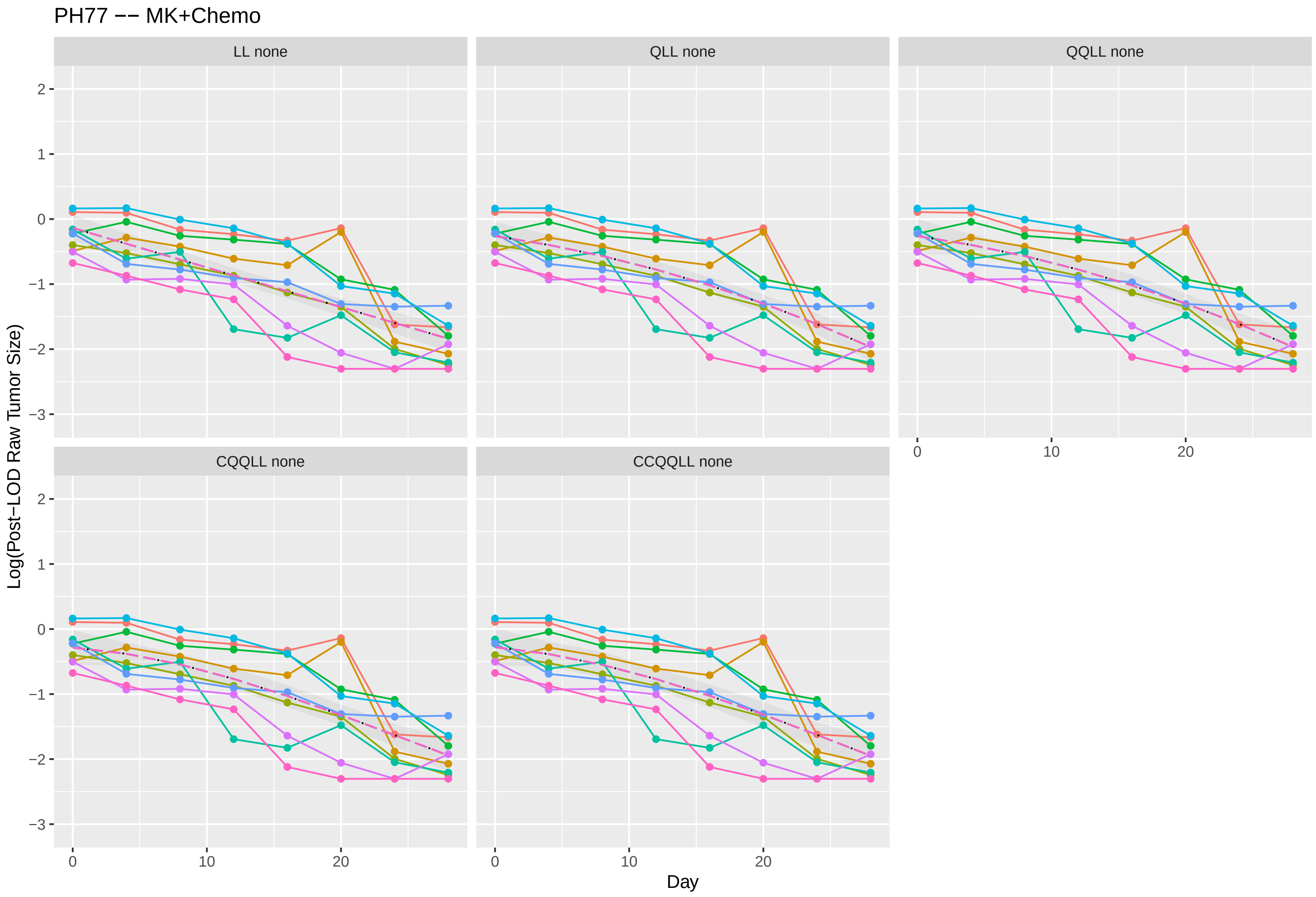

PH77 -- MK+Chemo
LL none
QLL none
QQLL none
CQQLL none
CCQQLL none
Log(Post-LOD Raw Tumor Size)
Day
2
1
0
−1
−2
−3
0
10
20

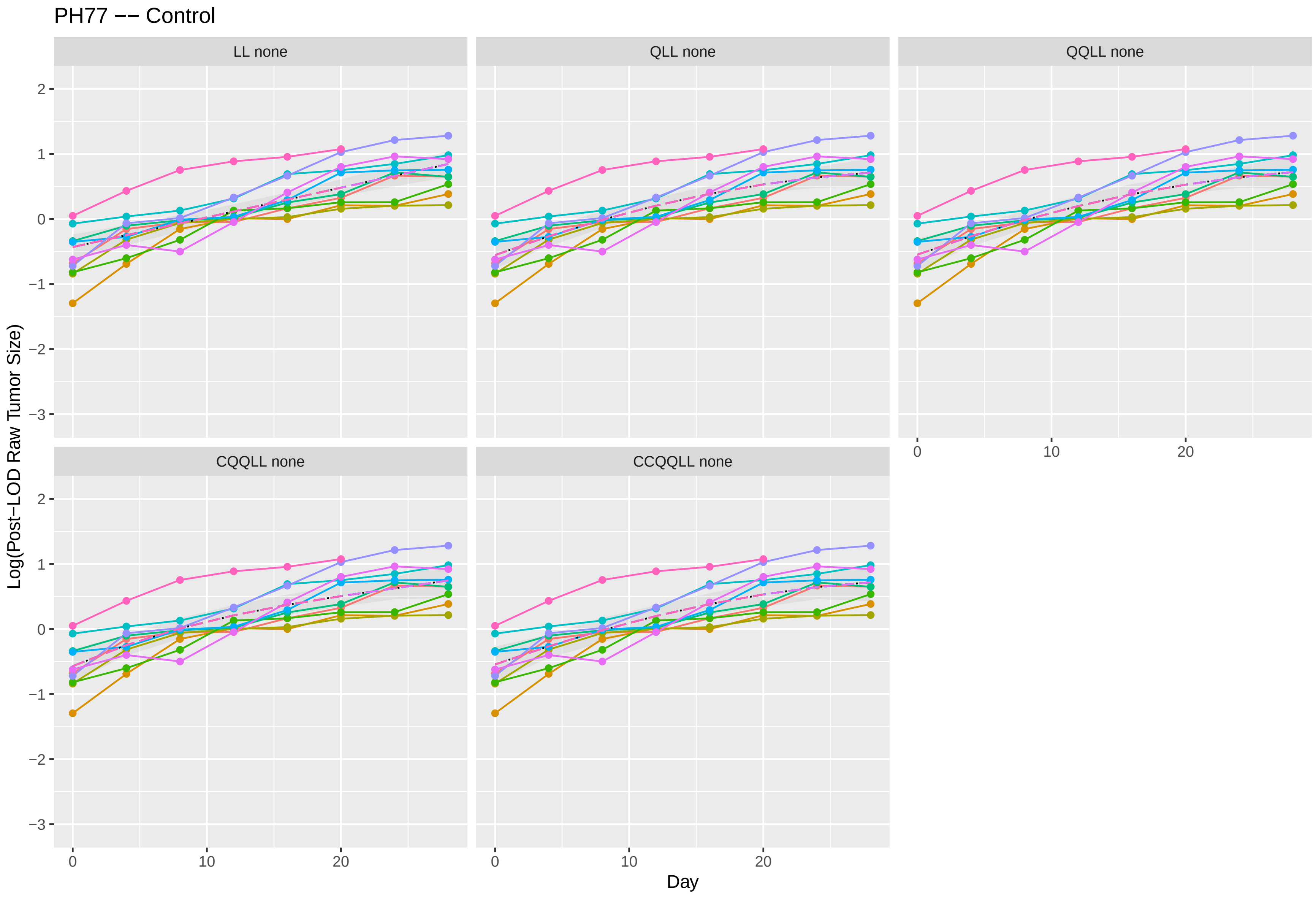

PH77 -- Control
LL none
QLL none
QQLL none
CQQLL none
CCQQLL none
Log(Post−LOD Raw Tumor Size)
Day
2
1
0
−1
−2
−3
0
10
20

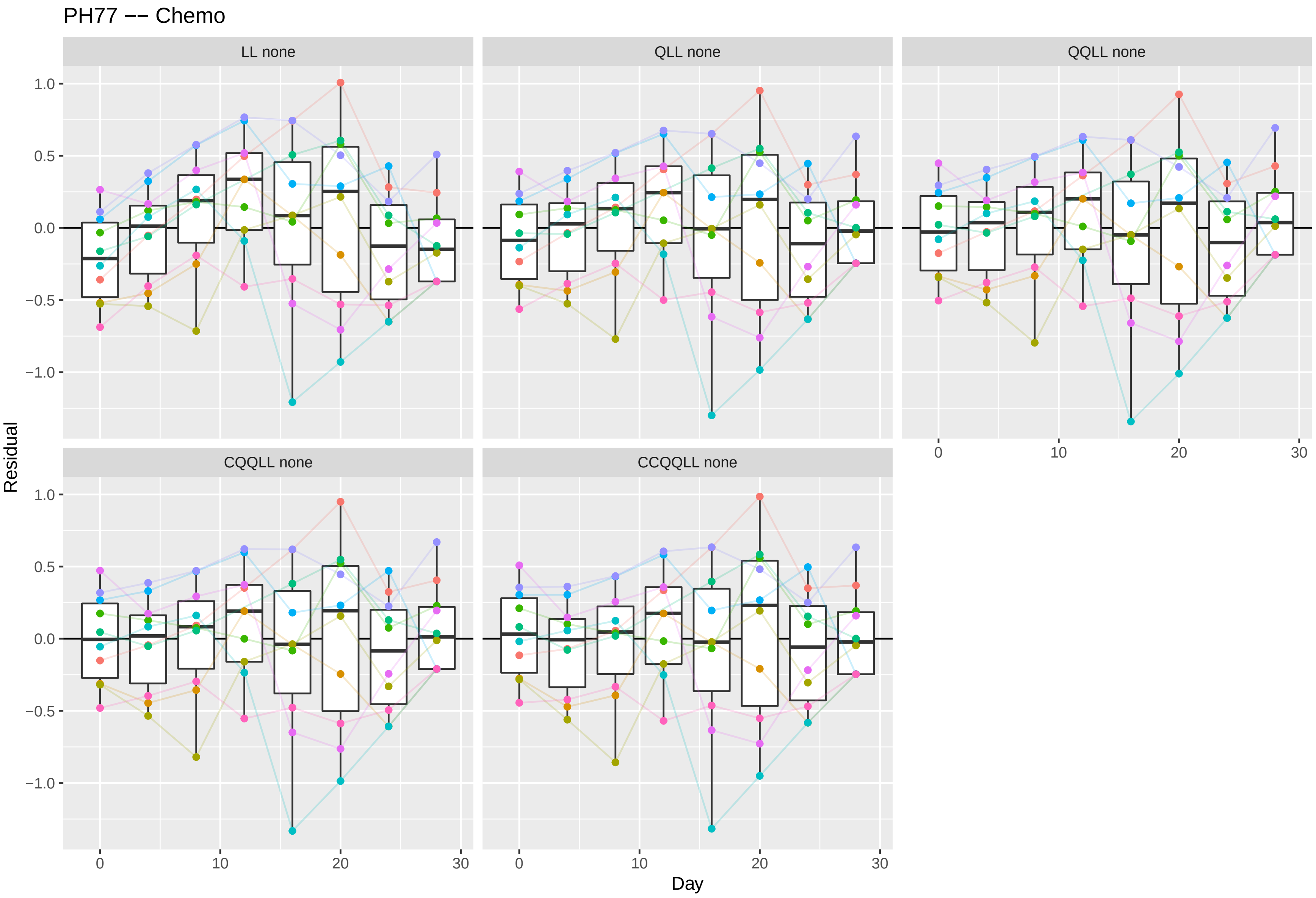
PH77 -- Chemo
LL none
QLL none
QQLL none
CQQLL none
CCQQLL none
Residual
Day
1.0
0.5
0.0
−0.5
−1.0
0
10
20
30

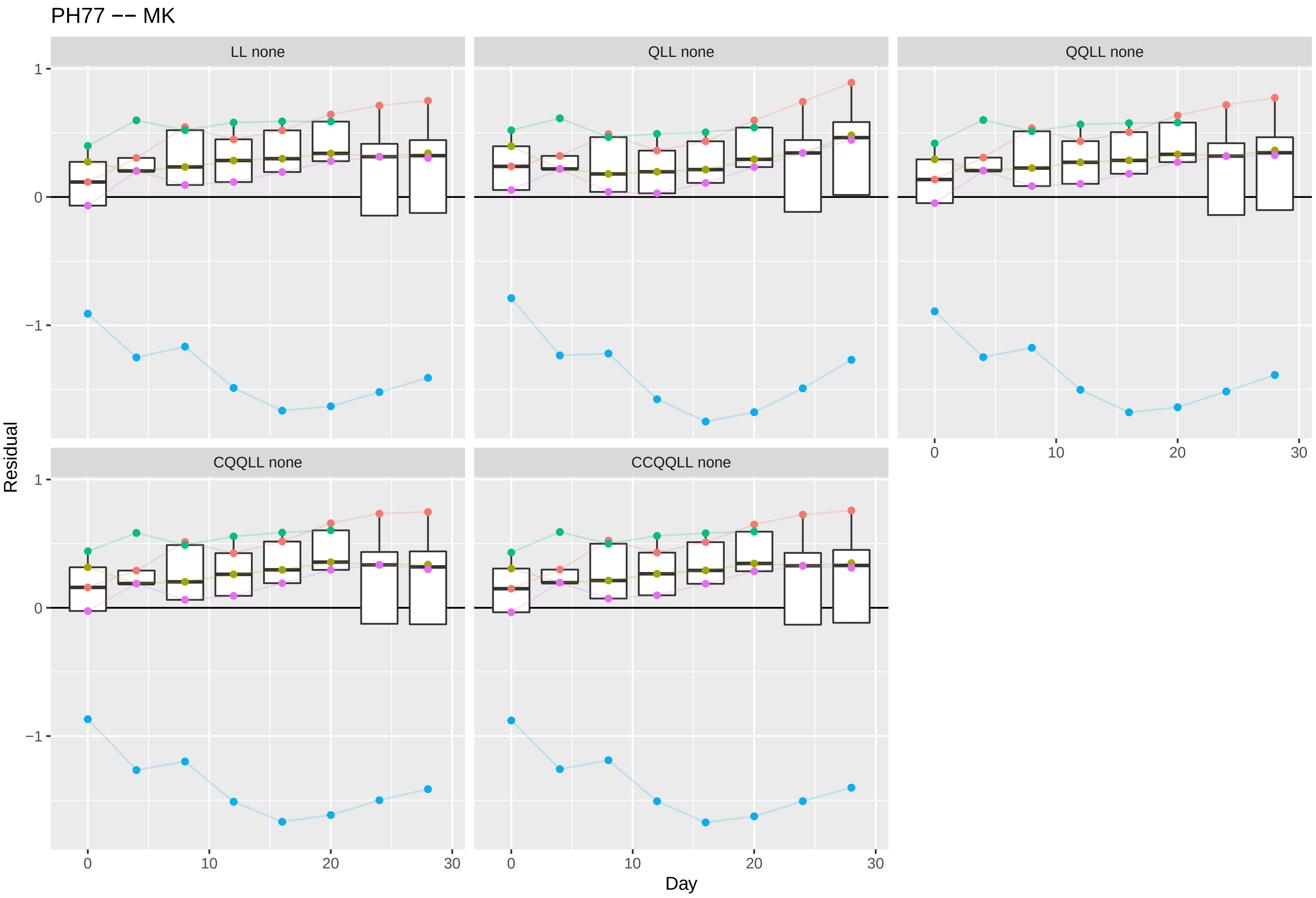
PH77 -- MK
LL none
QLL none
QQLL none
CQQLL none
CCQQLL none
Residual
Day
1
0
−1
0
10
20
30

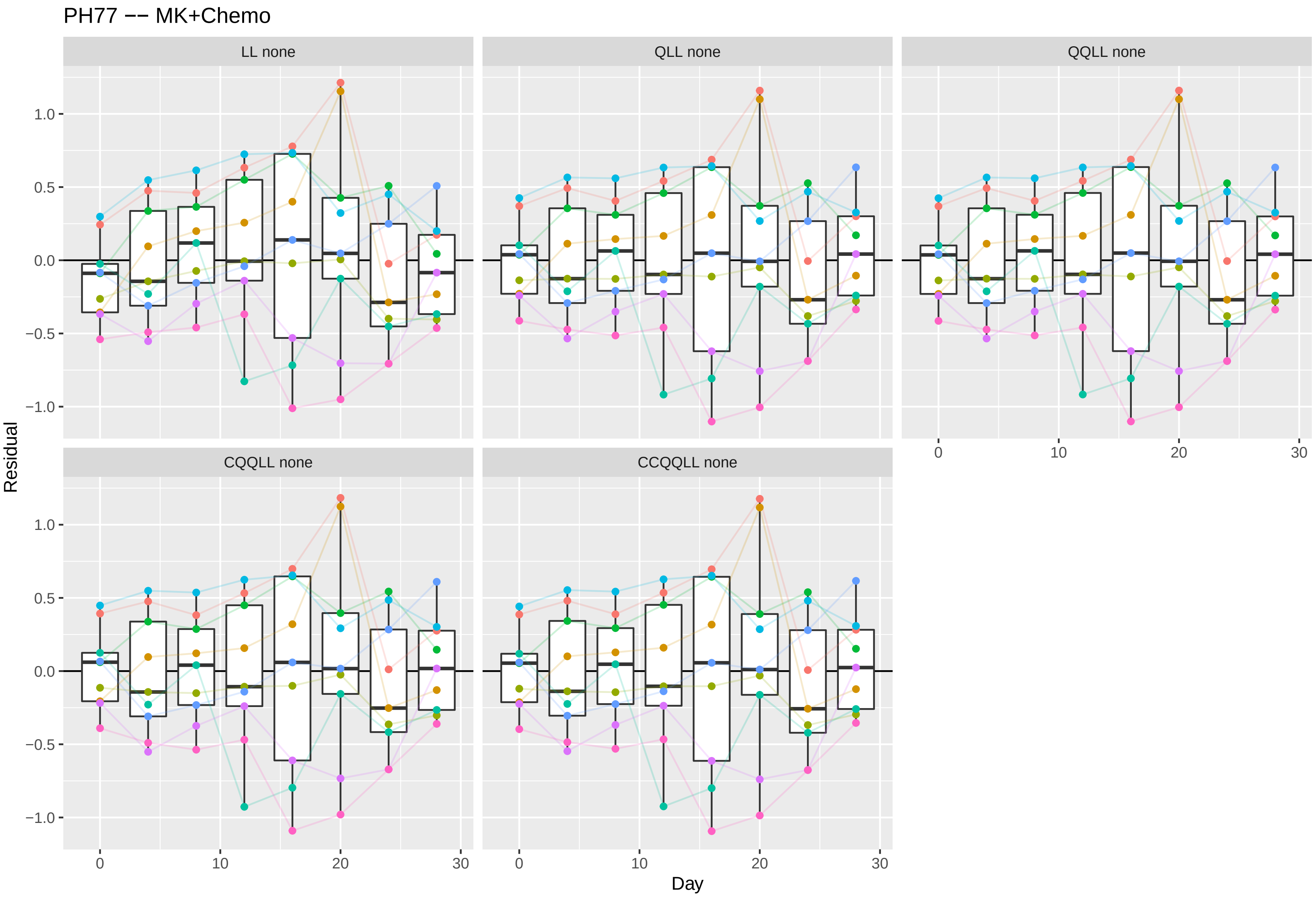
PH77 -- MK+Chemo
LL none
QLL none
QQLL none
CQQLL none
CCQQLL none
Residual
Day
1.0
0.5
0.0
−0.5
−1.0
0
10
20
30

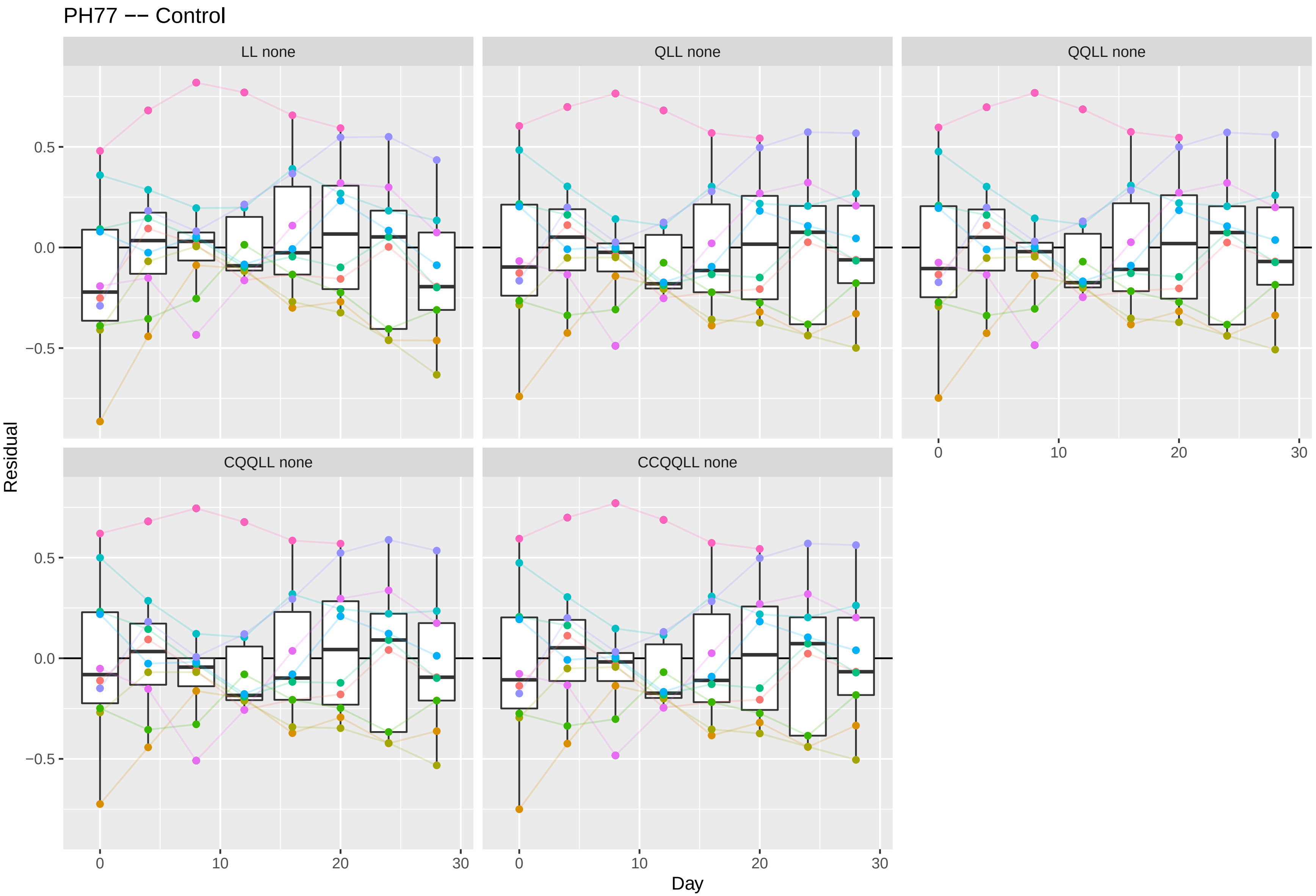
PH77 -- Control
LL none
QLL none
QQLL none
CQQLL none
CCQQLL none
Residual
Day
0.5
0.0
−0.5
0
10
20
30

PH95

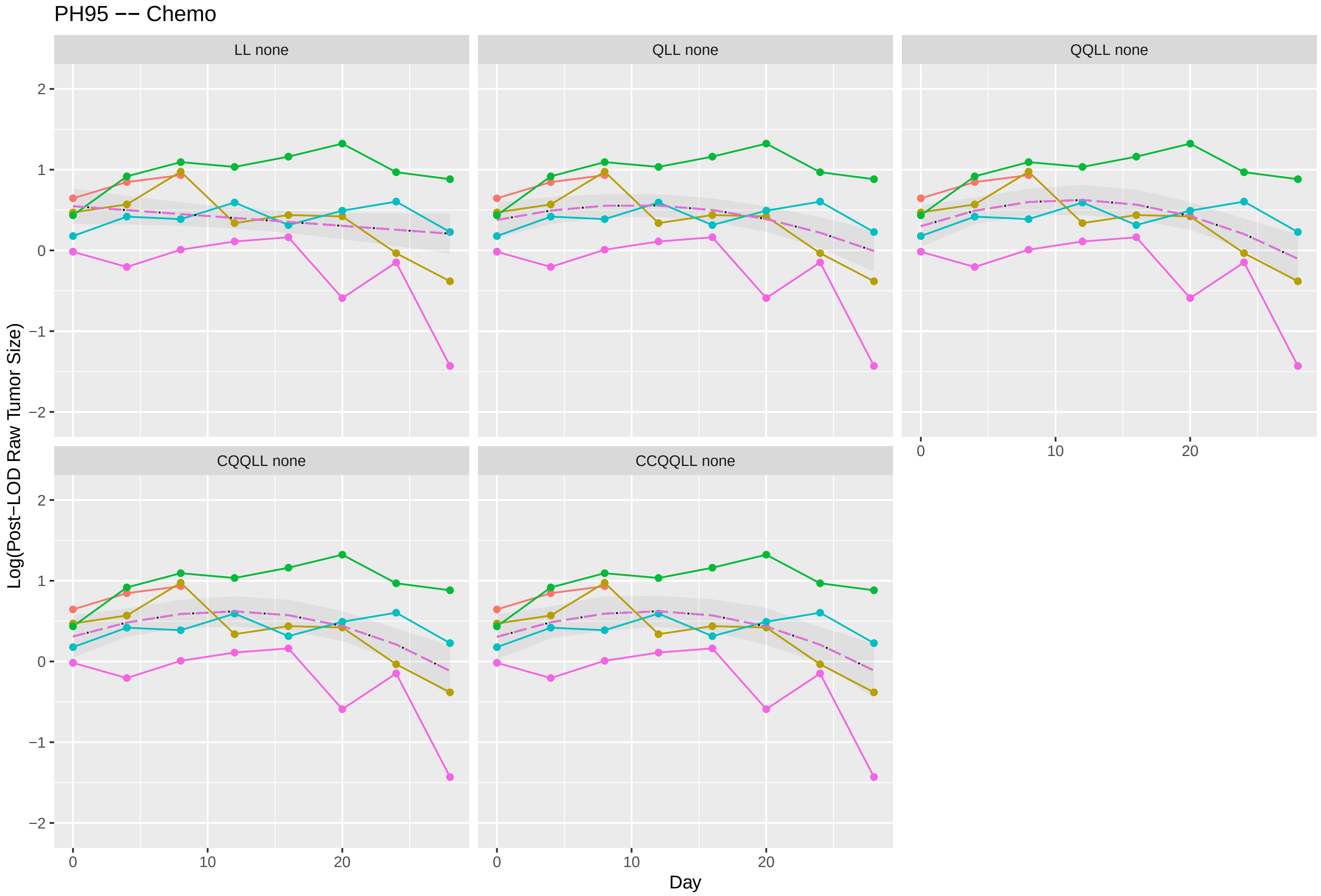

PH95 -- Chemo
LL none
QLL none
QQLL none
CQQLL none
CCQQLL none
Log(Post-LOD Raw Tumor Size)
Day
2
1
0
−1
−2
0
10
20

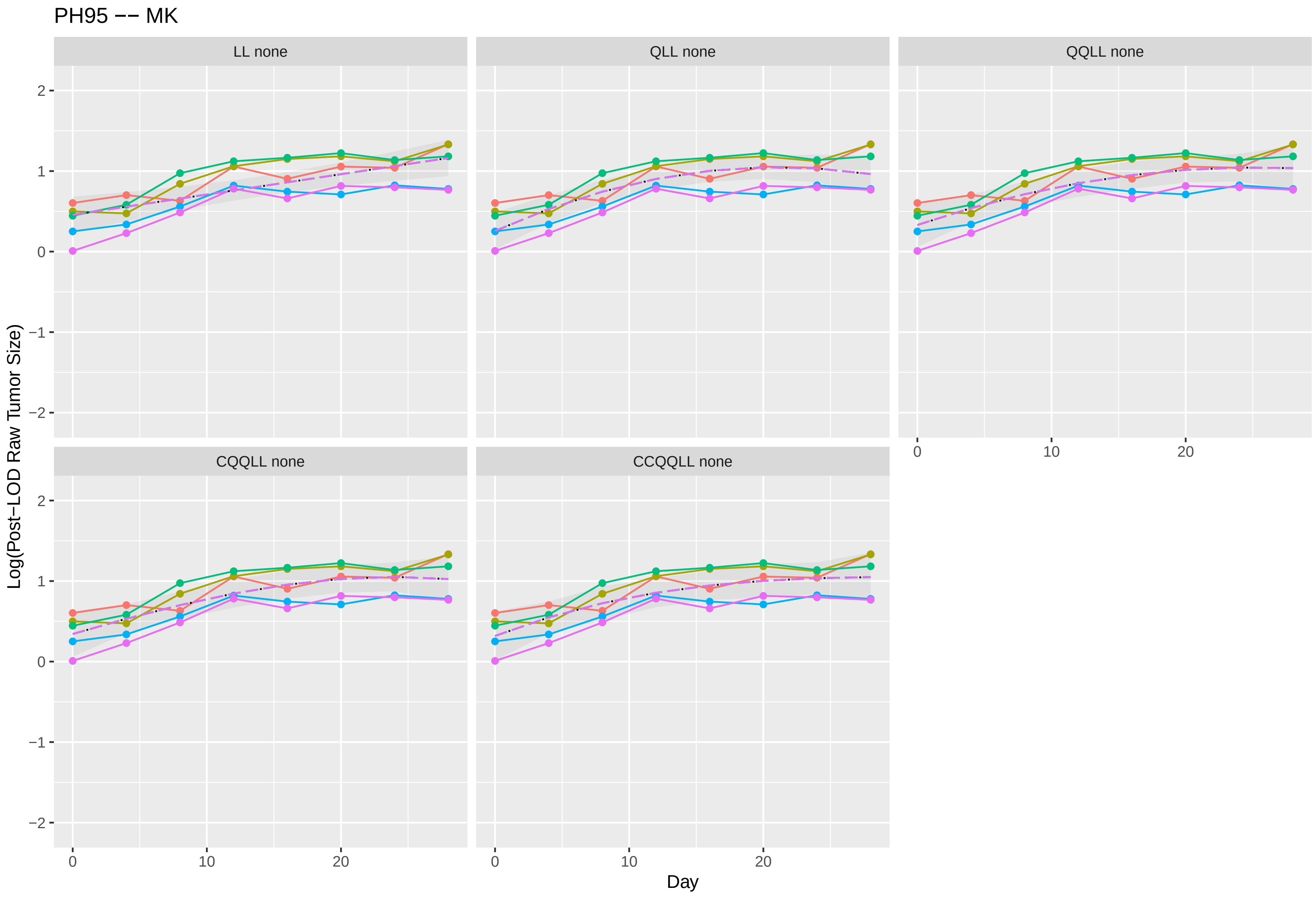
PH95 -- MK
LL none
QLL none
QQLL none
CQQLL none
CCQQLL none
Log(Post−LOD Raw Tumor Size)
Day
2
1
0
−1
−2
0
10
20

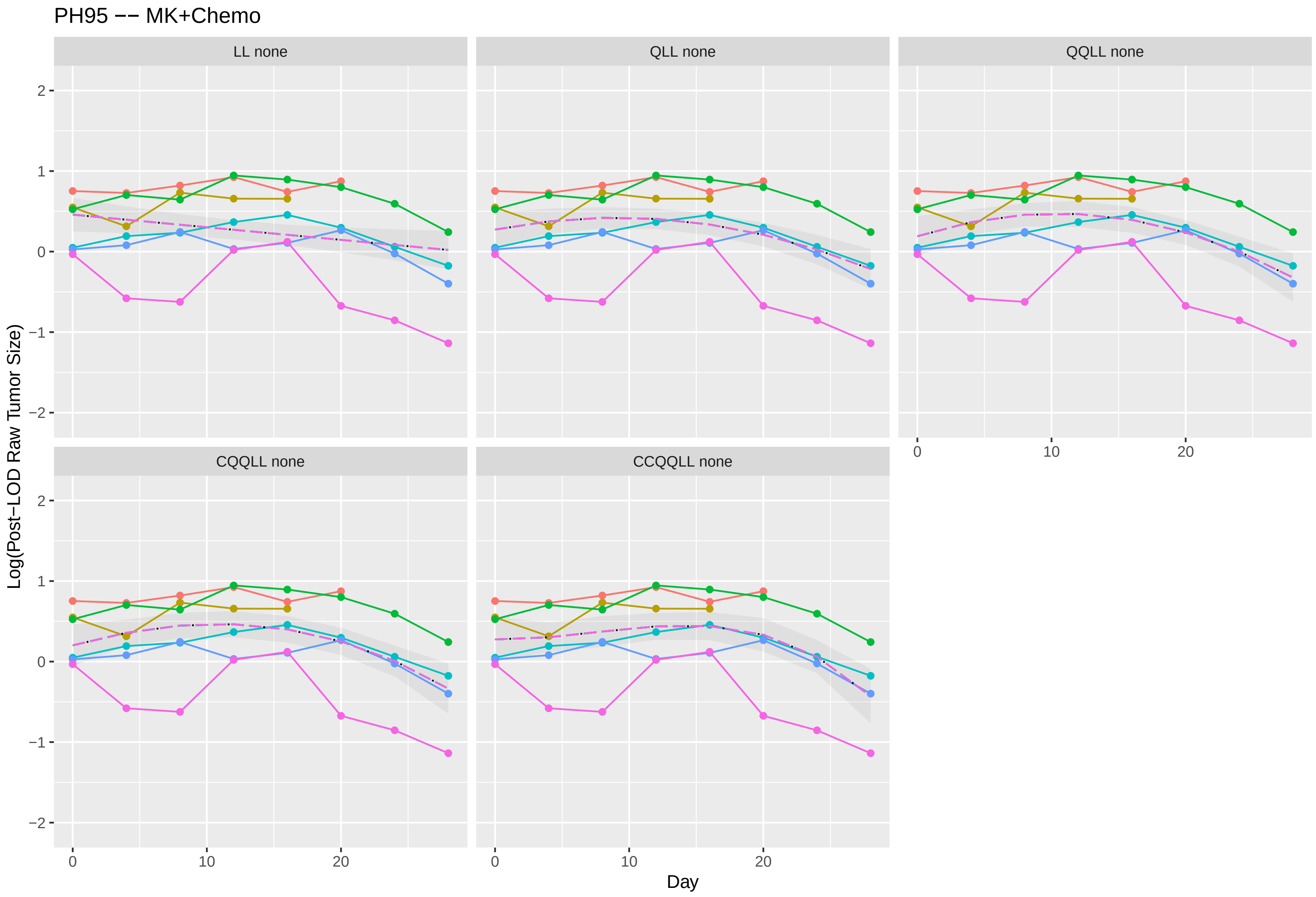

PH95 −− MK+Chemo
LL none
QLL none
QQLL none
CQQLL none
CCQQLL none
Log(Post−LOD Raw Tumor Size)
Day
2
1
0
−1
−2
0
10
20

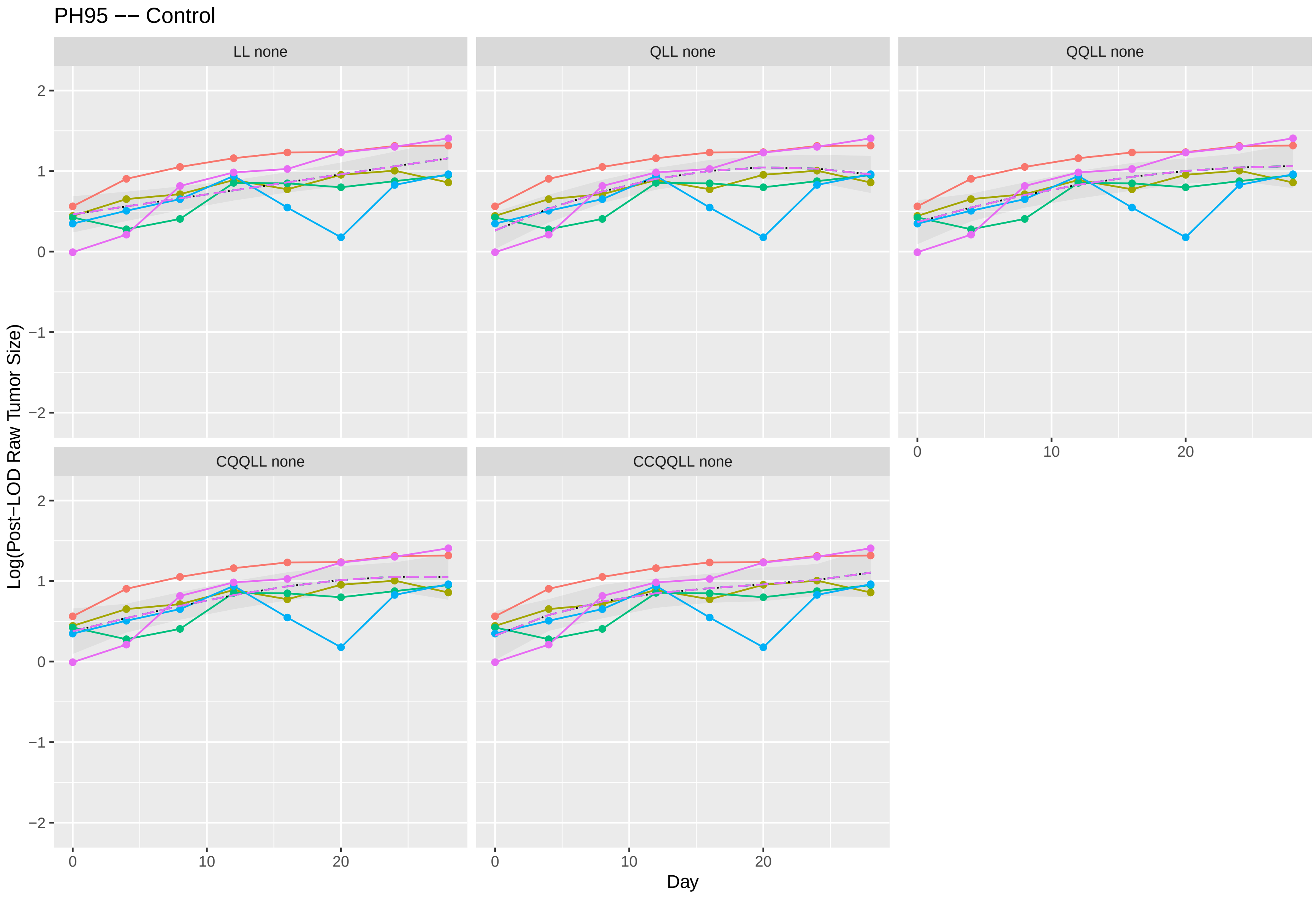
PH95 −− Control
LL none
QLL none
QQLL none
CQQLL none
CCQQLL none
Log(Post−LOD Raw Tumor Size)
Day
2
1
0
−1
−2
0
10
20

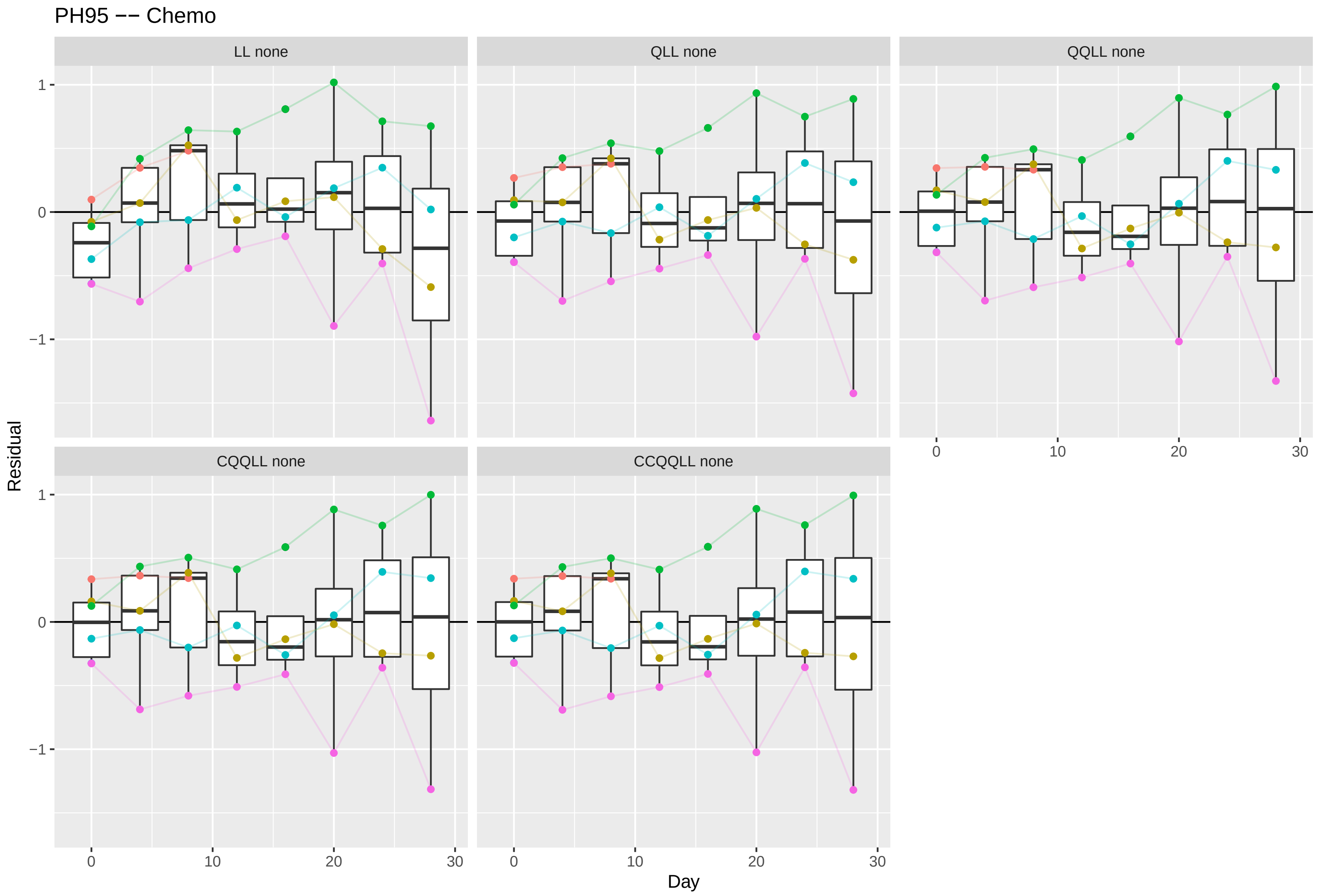
PH95 −− Chemo
LL none
QLL none
QQLL none
CQQLL none
CCQQLL none
Residual
Day
1
0
−1
0
10
20
30

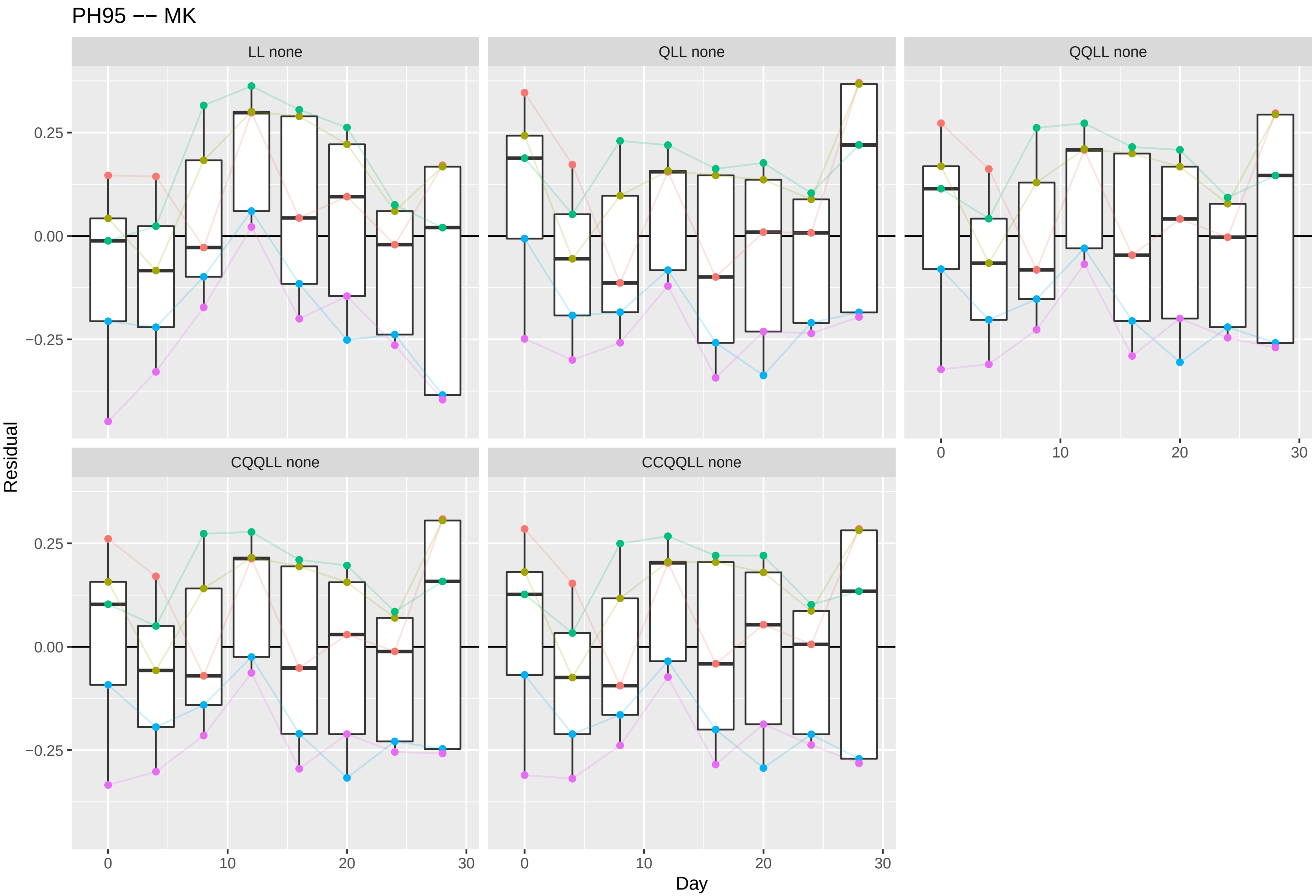
PH95 -- MK
LL none
QLL none
QQLL none
CQQLL none
CCQQLL none
Residual
Day
0.25
0.00
−0.25
0
10
20
30

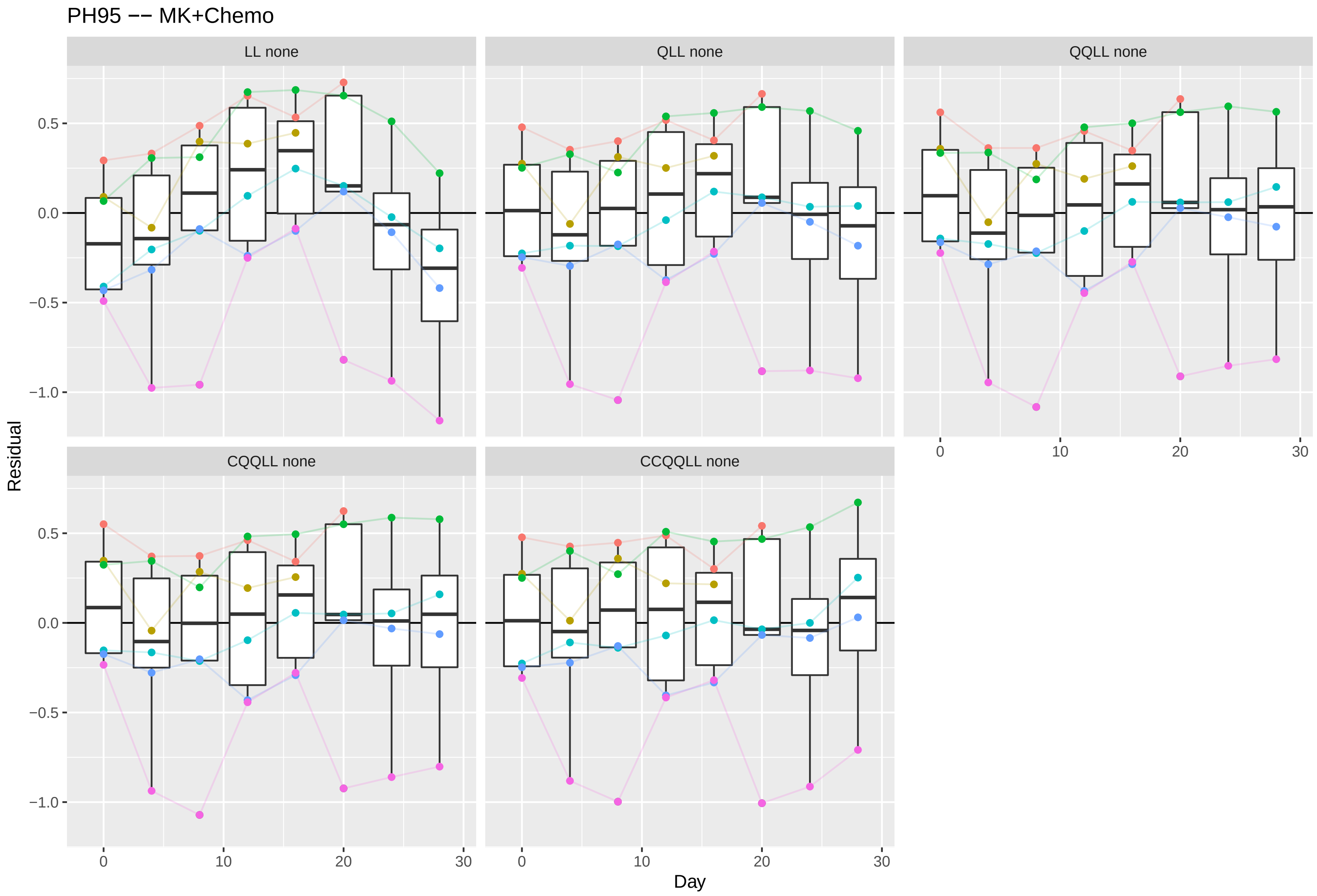
PH95 -- MK+Chemo
LL none
QLL none
QQLL none
CQQLL none
CCQQLL none
Residual
Day
0.5
0.0
−0.5
−1.0
0
10
20
30

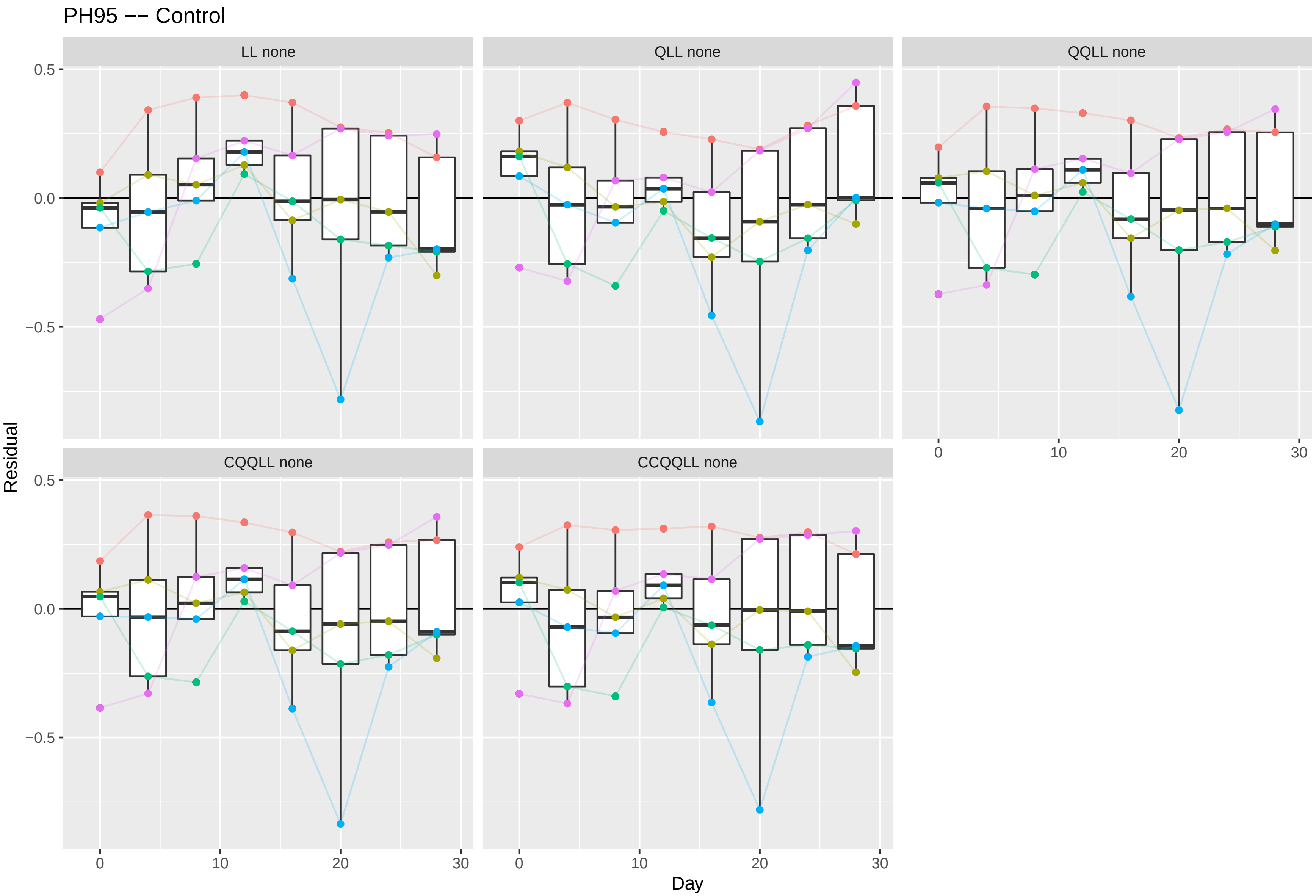

PH95 −− Control
LL none
QLL none
QQLL none
CQQLL none
CCQQLL none
Residual
Day
0.5
0.0
−0.5
0
10
20
30

PH39

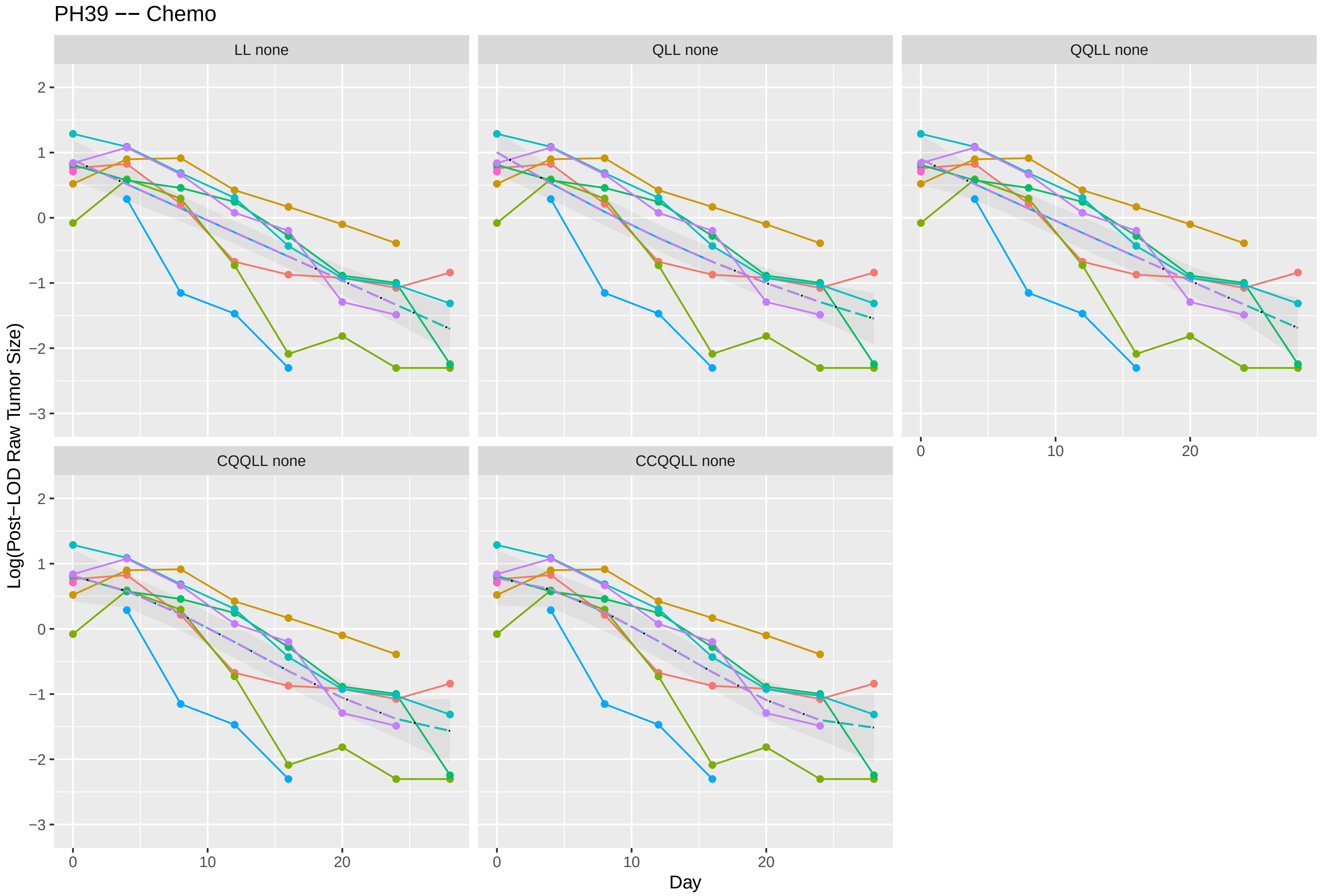

PH39 -- Chemo
LL none
QLL none
QQLL none
CQQLL none
CCQQLL none
Log(Post-LOD Raw Tumor Size)
Day
2
1
0
−1
−2
−3
0
10
20

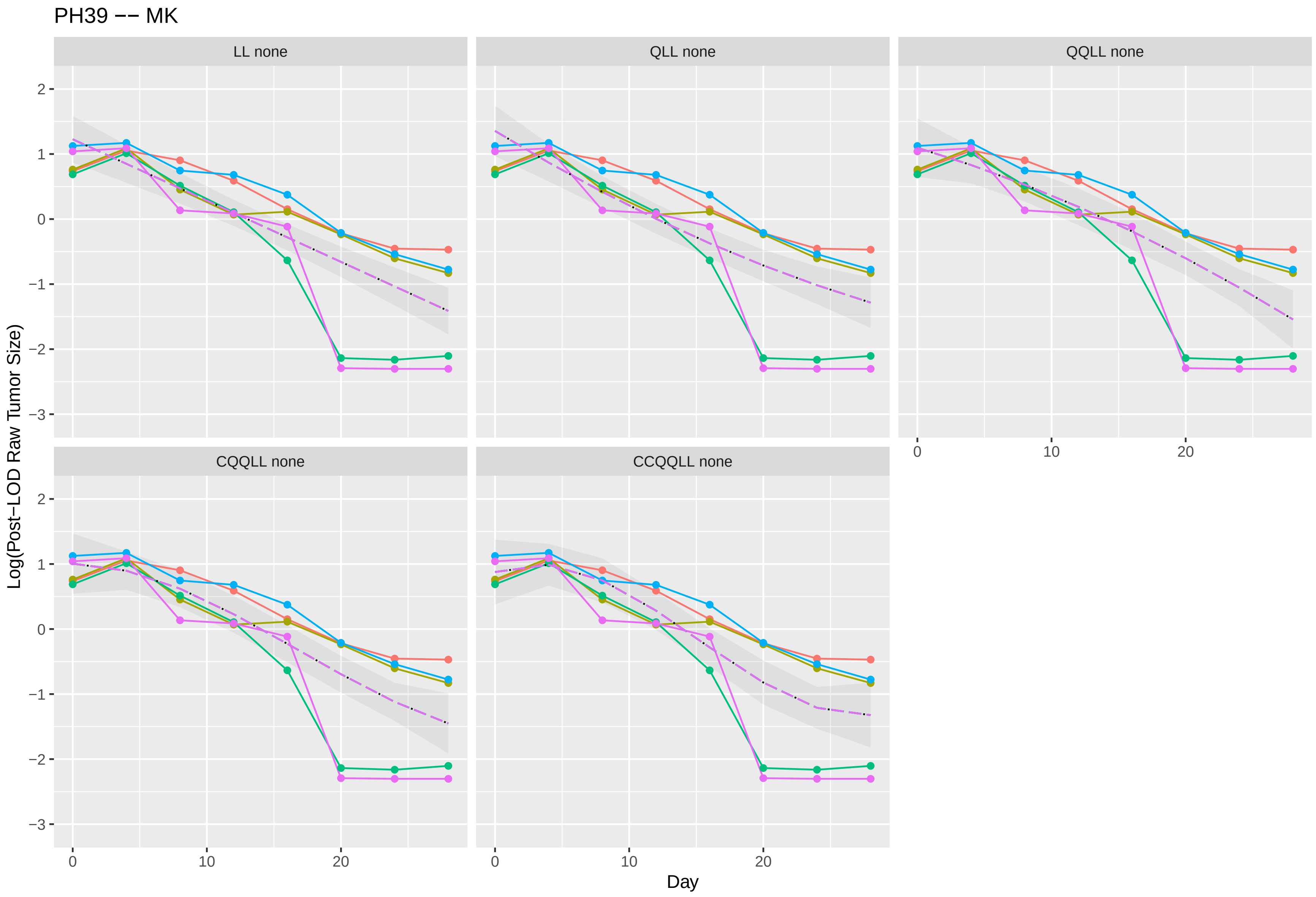
PH39 -- MK
LL none
QLL none
QQLL none
CQQLL none
CCQQLL none
Log(Post−LOD Raw Tumor Size)
Day
2
1
0
−1
−2
−3
0
10
20

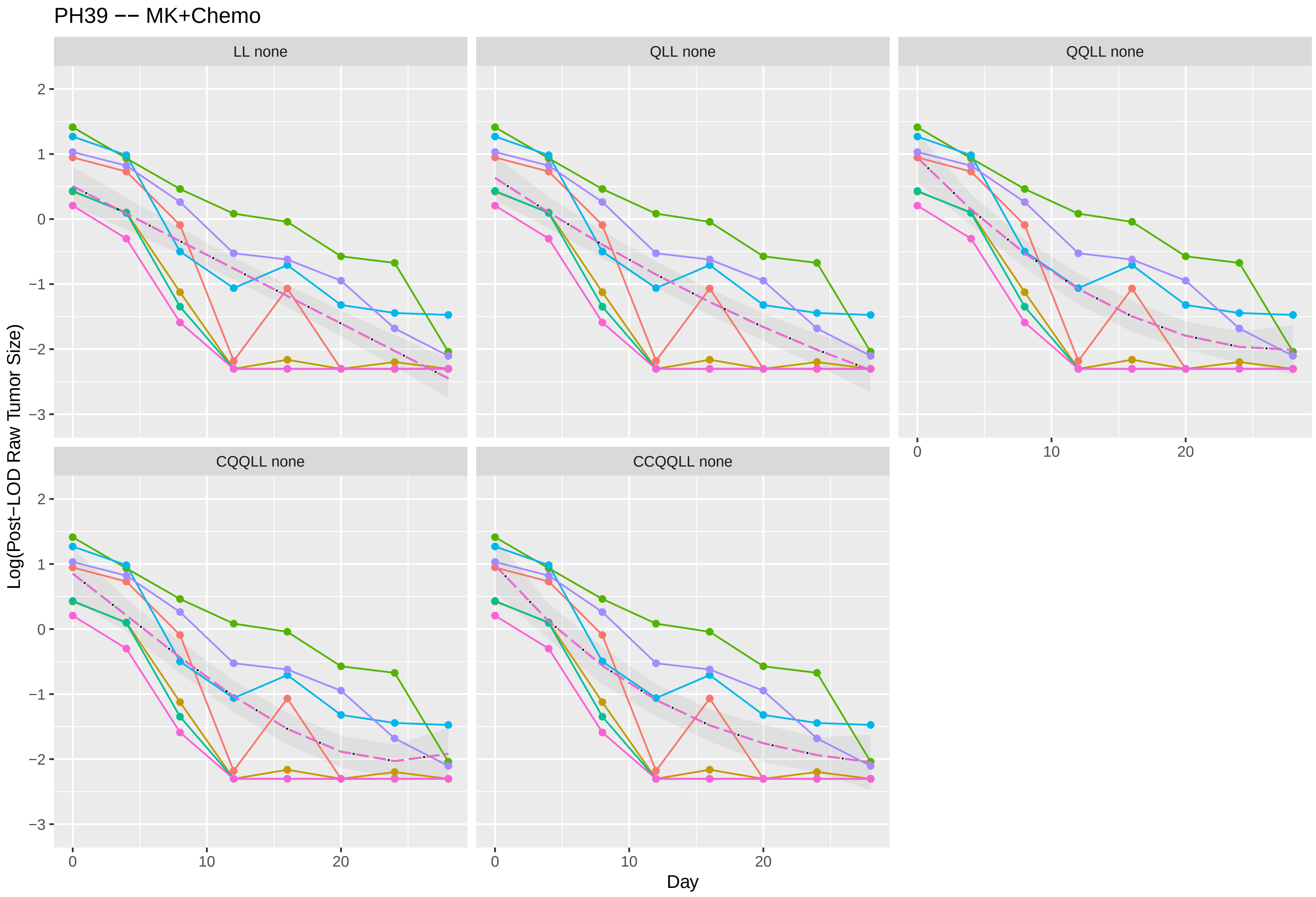

PH39 −− MK+Chemo
LL none
QLL none
QQLL none
CQQLL none
CCQQLL none
Log(Post−LOD Raw Tumor Size)
Day
2
1
0
−1
−2
−3
0
10
20

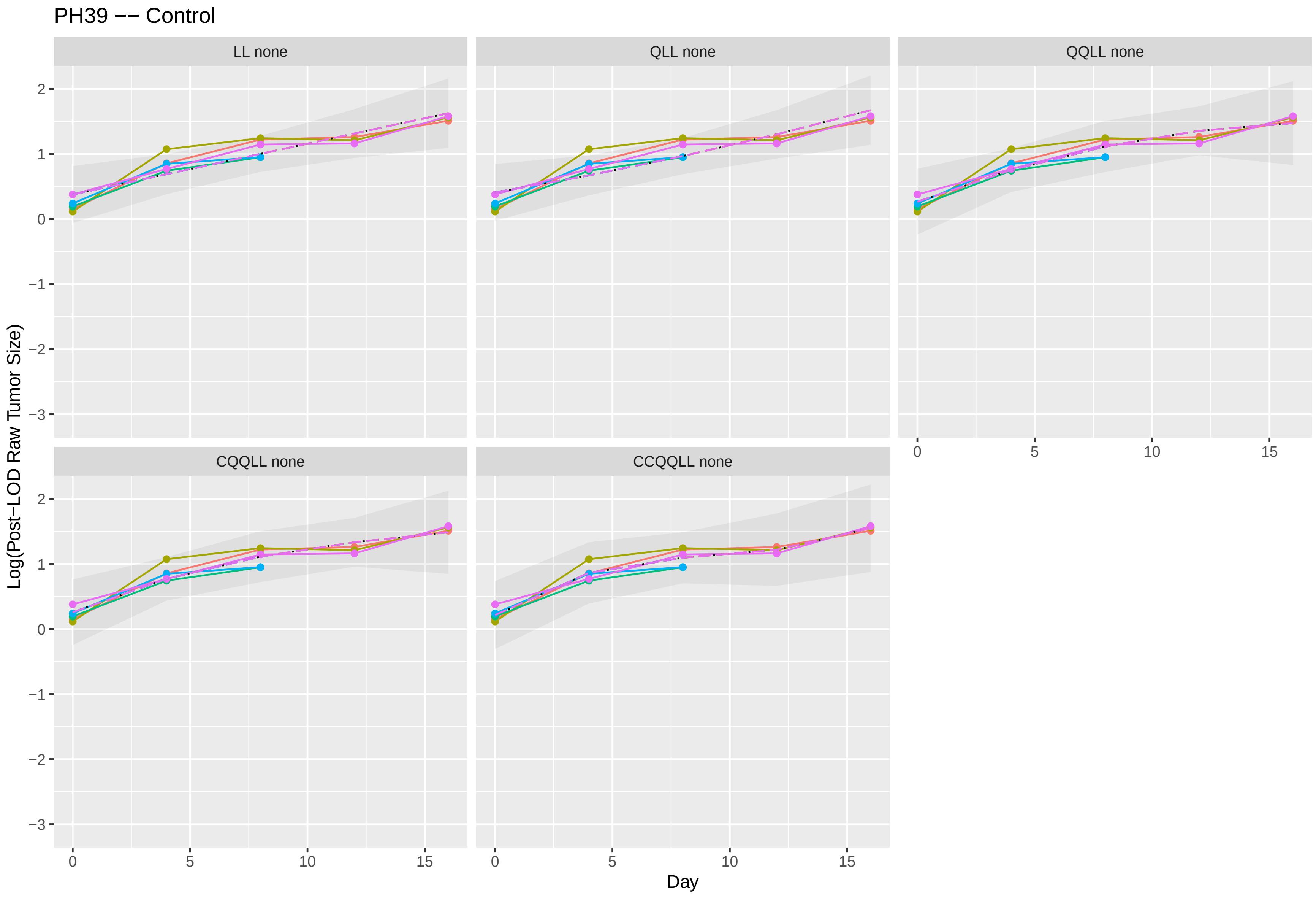

PH39 −− Control
LL none
QLL none
QQLL none
CQQLL none
CCQQLL none
Log(Post−LOD Raw Tumor Size)
Day
2
1
0
−1
−2
−3
0
5
10
15

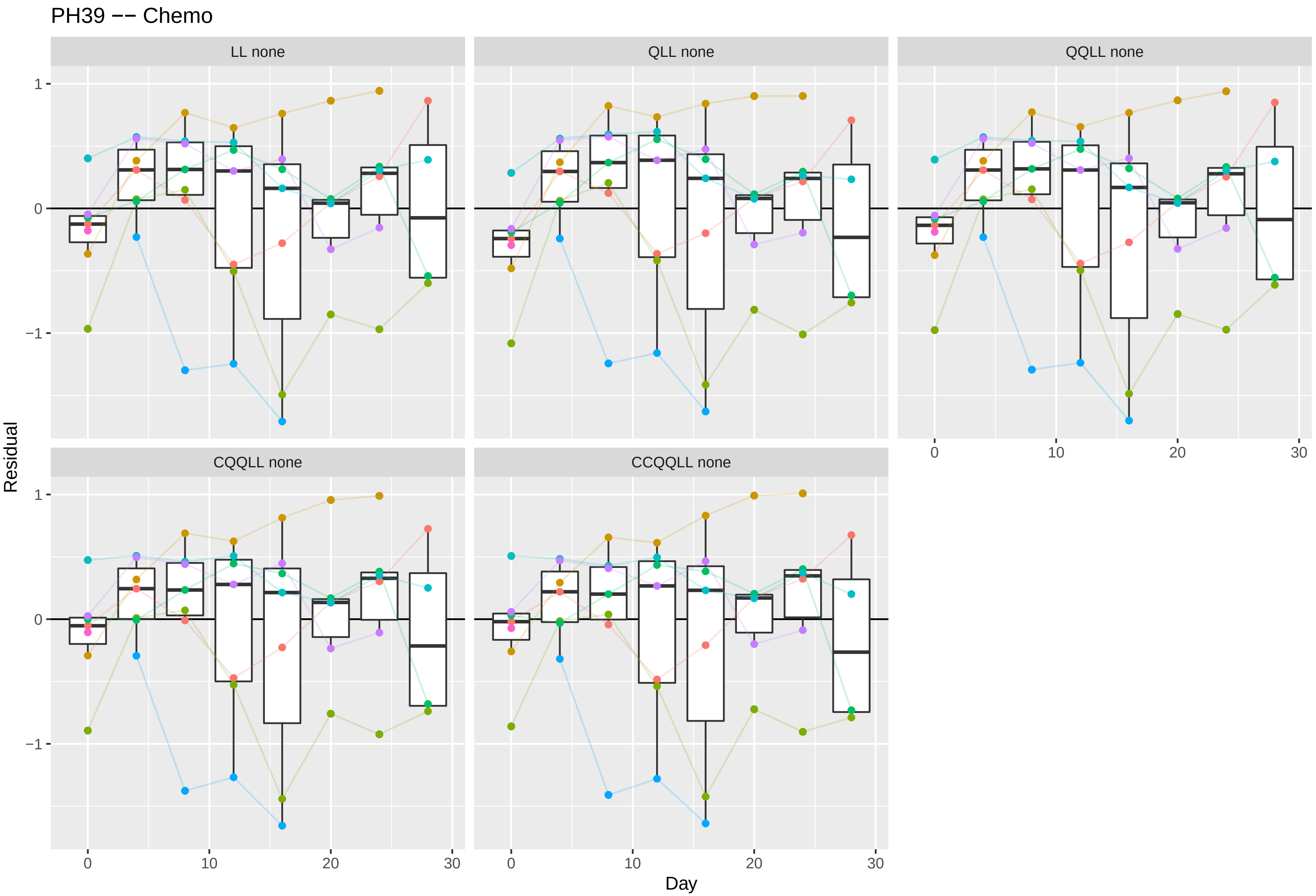

PH39 −− Chemo
LL none
QLL none
QQLL none
CQQLL none
CCQQLL none
Residual
Day
1
0
−1
0
10
20
30

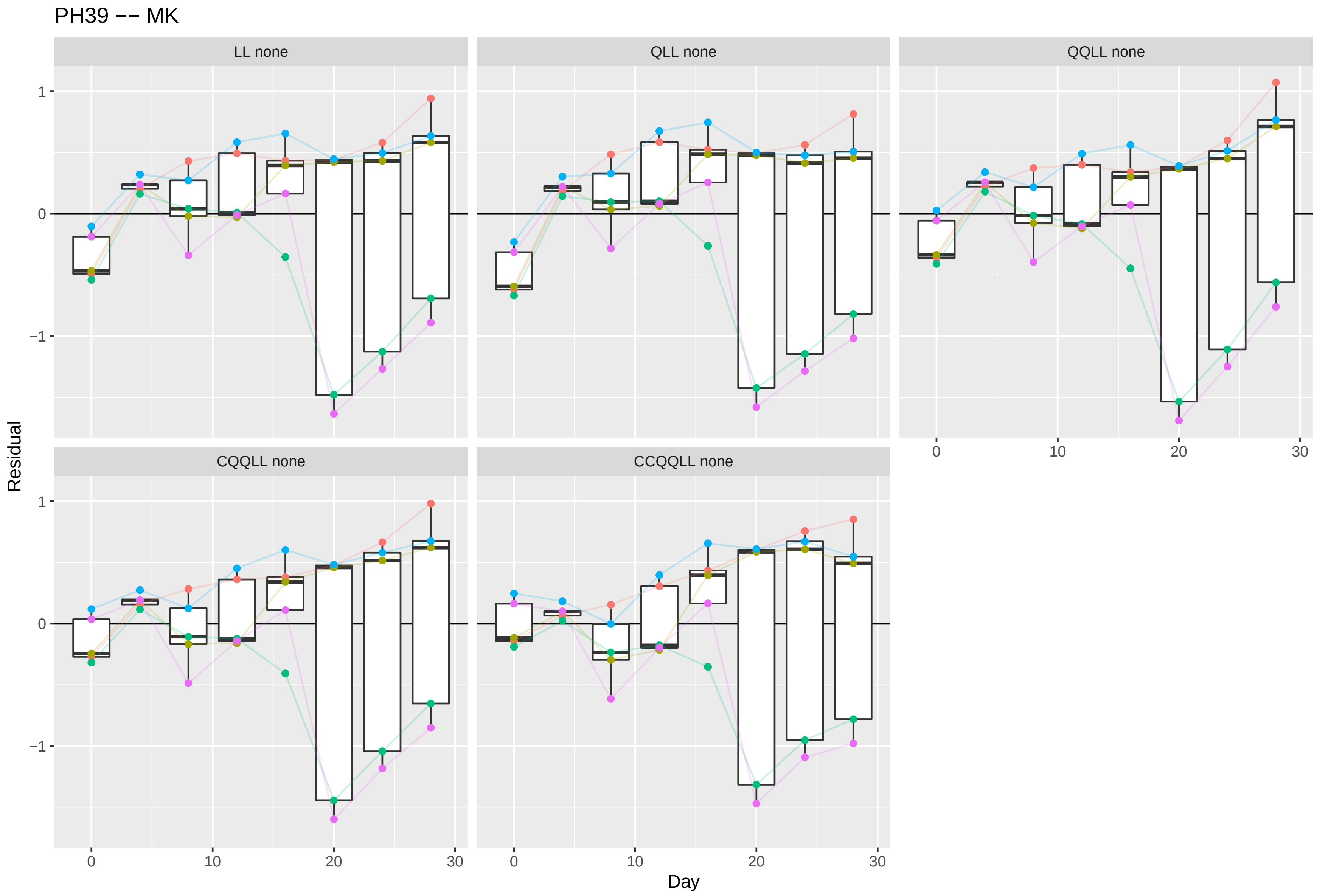

PH39 -- MK
LL none
QLL none
QQLL none
CQQLL none
CCQQLL none
Residual
Day
1
0
−1
0
10
20
30

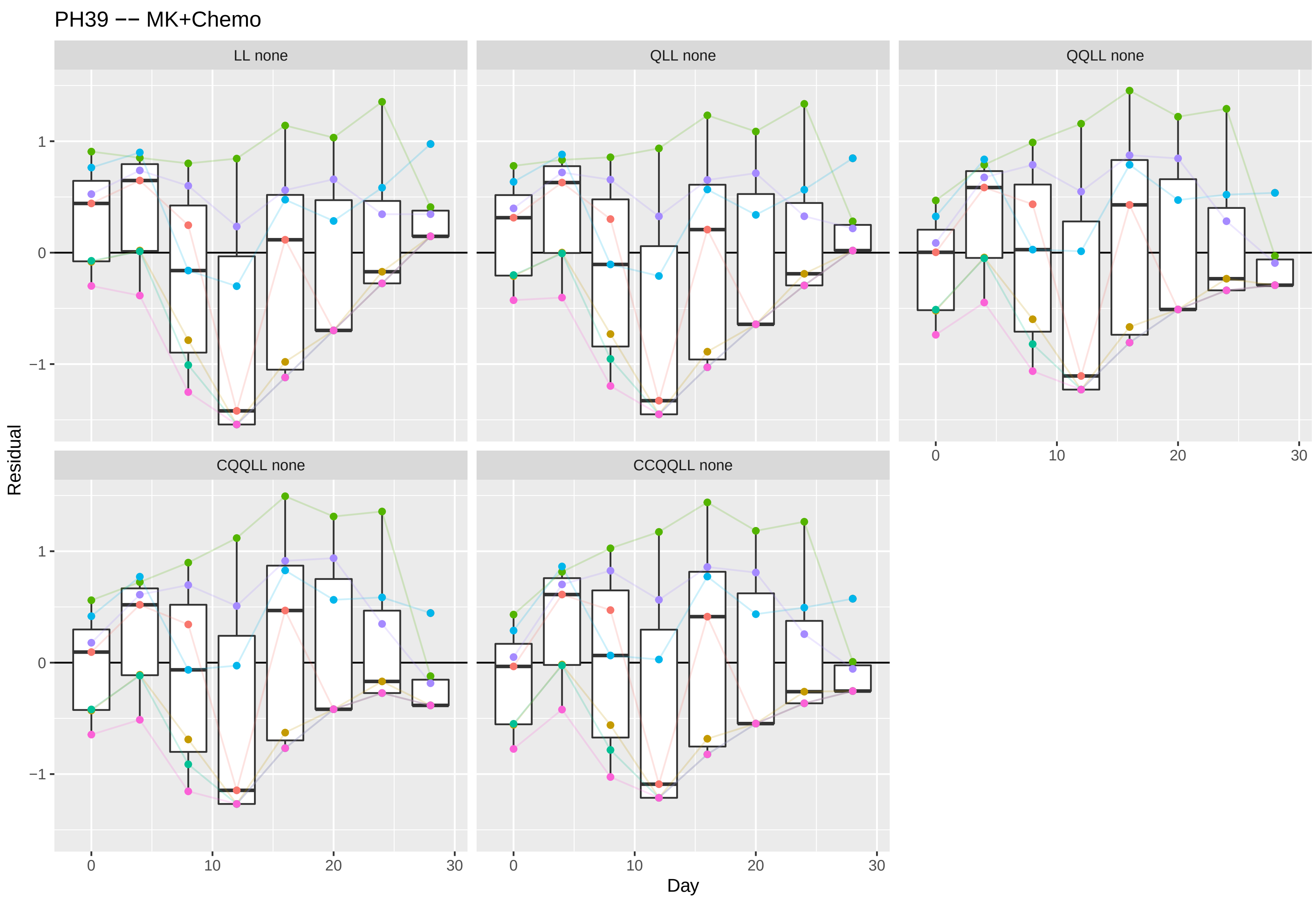
PH39 -- MK+Chemo
LL none
QLL none
QQLL none
CQQLL none
CCQQLL none
Residual
Day
1
0
−1
0
10
20
30

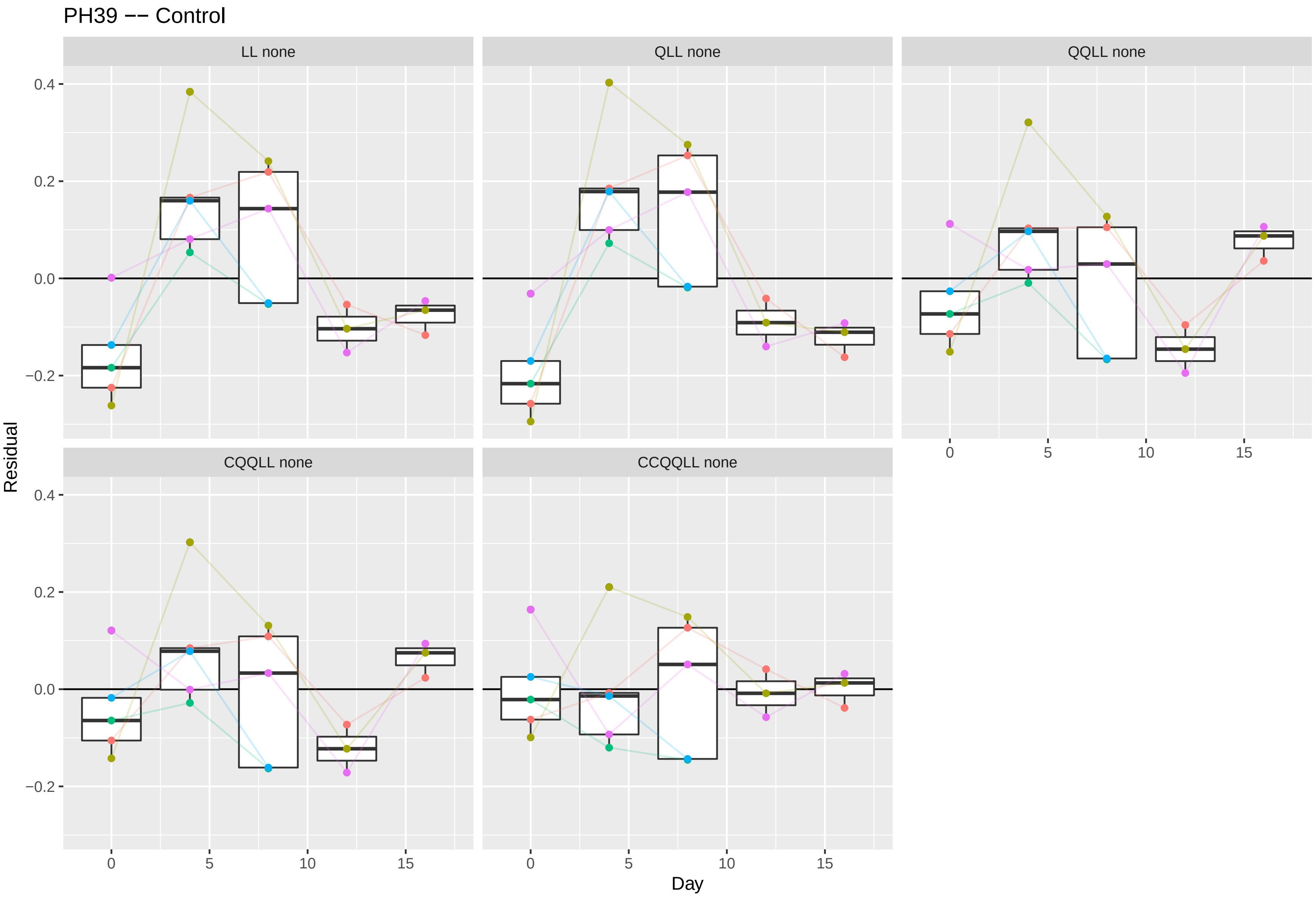
PH39 -- Control
LL none
QLL none
QQLL none
CQQLL none
CCQQLL none
Residual
Day
0.4
0.2
0.0
−0.2
0
5
10
15

Supplemental Table 1. Overview of the modeling steps, the figures and metrics used for each, and any special considerations for each step.

Step	Useful plots and metrics	Questions to evaluate	Notes to consider
Evaluate the data, randomization	• Growth trajectories on raw and modeling scale<br>• Baseline tumor burden jitter plots	• Are there outlying data values that should be verified?<br>• Do baseline distributions overlap?	• Tumor sizes outside of the general trajectory for a given mouse may represent errors in the measurement or data entry process that could be corrected<br>• If distributions of baseline tumor sizes do not overlap, this is a possible explanation for observed treatment effects to consider when interpreting final hypothesis testing results
1. Fit the mean model: determine functional form of mean model	• Growth trajectories on raw and modeling scale overlaid with model predicted trajectories<br>• Residual plots from ML independence fit<br>• AIC/BIC from ML fits with candidate linear, quadratic, cubic models<br>• Parameter estimates	• Are trajectories straight or curved?<br>• Does curvature vary between arms?<br>• For which candidate model do predicted mean trajectories best describe observed trajectories?<br>• Do residual plots show trends?<br>• Which models fit well according to information criteria, deferring slightly more to BIC than to AIC in favor of parsimony?	• Balance parsimony versus describing the data well
2. Determine covariance structure	• Semivariogram plots of variance and covariance as a function of lag<br>• Printed matrices of biologically plausible covariance structures<br>• AIC/BIC from REML fits of candidate structures holding mean model constant	• Does variance appear to be constant or increasing over time?<br>• Does the covariance taper off (decrease as lag increases) to 0? Or to a number greater than 0?<br>• Are parameter estimates within or nearly at the edges of the parameter space?<br>• Which structure is a good representation of the unstructured parameter estimates or covariance matrix computed from independence model residuals?<br>• If reporting multiple PDXs, is there one structure that provides a reasonable representation of variance for all PDXs for simplicity of reporting?	• Balance parsimony versus describing the data well<br>• Parameter estimates on the boundary of the parameter space likely indicates an over-specified covariance model
3. Re-fit mean model with the chosen	• Growth trajectories overlaid with model fits	• Do residual plots and model fit metrics still support the decisions made?	• Remember that the goal is a useful and parsimonious model

covariance structure	• Residual plots from REML fit		
4. Perform hypothesis testing and draw conclusions regarding activity relative to control and comparisons between active arms	• Multiple degree of freedom test of coincident curves; if significant proceed to understand source of differences<br>• 1 degree of freedom test of equal slope (growth rates)<br>• 1 degree of freedom test of equal intercept (AUC in centered time model)<br>• Plot model predicted growth trajectories together with confidence bands and per-arm sample size over time<br>• Compute estimates of time to tumor doubling if of interest	• Is there evidence that growth trajectories are not coincident, i.e., differ in growth rate and/or in AUC?<br>• If so, is there evidence that growth rates differ?<br>• Is there evidence that AUCs differ?	• Significant coincident curve test leads to conclusion that the curves are not coincident<br>• Significant test of slope leads to conclusion that growth rates differ between arms<br>• Significant test of intercept in a centered time model leads to conclusion that AUCs differ between arms<br>• Graphics should reflect hypothesis testing results<br>• Differences in AUC and not growth rate can indicate delay of tumor shrinkage in one arm relative to another

Supplemental Table 2. Characteristics of covariance matrices commonly considered for repeated measures data, where "X" indicates the property is applicable.

	Unstructured	Toeplitz	Random Effects	Spatial(power)	Spatial(power) plus random effects	Compound symmetric or Exchangeable
Abbreviation	UN	Toep	RE	sp(pow)	sp(pow)+RE	CS
# Covariance parameters	m(m+1)/2=36	m=8	4	2	3	2
Dominant source of variation	Between	Within	Between	Within	Combination of Between, Within	Between
Makes most sense with equal time spacing of measurements	X	X	Allowed but not required	Allowed but not required	Allowed but not required	Allowed but not required
Variance constant over time	Allowed but not required	X		X	X	X
Covariance depends on lag	Allowed but not required	X		X	X	
Covariance depends on precise time of measurement	X		X			
Covariance tapers off	Allowed but not required	Allowed but not required		Yes, to 0 as an exponential function of lag	Yes, to the between-mouse covariance	
Extensions or special cases (may alter number of parameters)		Allow non-constant variance; Allow covariance to be thresholded at 0 beyond a fixed lag	Higher order polynomial terms can be specified as random if the data supports their estimation	Reduces to autoregressive of order 1 (AR(1)) in the case of equal spacing; Allow non-constant variance with AR(1);	Reduces to AR(1)+RE in the case of equal spacing; Allow non-constant variance with AR(1);	Allow non-constant variance

Footnotes

1. m = number of time points (m=8 in the present experiment)
2. Between mouse sources: correlation due to a mouse being generally high or low for all observations
3. Within mouse sources: correlation due to fluctuations about a smooth trend and measurement error
4. RE: in the case of random slope and intercept

Supplemental Table 3. Mathematical representation of the covariance structures in matrix format for the chemotherapy±MK case study with m=8 observation time points.

**Name (abbreviation)**	**Mathematical representation**
Compound Symmetry (CS)	$\begin{bmatrix} \sigma^2+\sigma_1 & \sigma_1 & \sigma_1 & \sigma_1 & \sigma_1 & \sigma_1 & \sigma_1 & \sigma_1 \\ \sigma_1 & \sigma^2+\sigma_1 & \sigma_1 & \sigma_1 & \sigma_1 & \sigma_1 & \sigma_1 & \sigma_1 \\ \sigma_1 & \sigma_1 & \sigma^2+\sigma_1 & \sigma_1 & \sigma_1 & \sigma_1 & \sigma_1 & \sigma_1 \\ \sigma_1 & \sigma_1 & \sigma_1 & \sigma^2+\sigma_1 & \sigma_1 & \sigma_1 & \sigma_1 & \sigma_1 \\ \sigma_1 & \sigma_1 & \sigma_1 & \sigma_1 & \sigma^2+\sigma_1 & \sigma_1 & \sigma_1 & \sigma_1 \\ \sigma_1 & \sigma_1 & \sigma_1 & \sigma_1 & \sigma_1 & \sigma^2+\sigma_1 & \sigma_1 & \sigma_1 \\ \sigma_1 & \sigma_1 & \sigma_1 & \sigma_1 & \sigma_1 & \sigma_1 & \sigma^2+\sigma_1 & \sigma_1 \\ \sigma_1 & \sigma_1 & \sigma_1 & \sigma_1 & \sigma_1 & \sigma_1 & \sigma_1 & \sigma^2+\sigma_1 \end{bmatrix}$
Autoregressive of order 1 [AR(1)]	$\begin{bmatrix} 1 & \rho & \rho^2 & \rho^3 & \rho^4 & \rho^5 & \rho^6 & \rho^7 \\ \rho & 1 & \rho & \rho^2 & \rho^3 & \rho^4 & \rho^5 & \rho^6 \\ \rho^2 & \rho & 1 & \rho & \rho^2 & \rho^3 & \rho^4 & \rho^5 \\ \rho^3 & \rho^2 & \rho & 1 & \rho & \rho^2 & \rho^3 & \rho^4 \\ \rho^4 & \rho^3 & \rho^2 & \rho & 1 & \rho & \rho^2 & \rho^3 \\ \rho^5 & \rho^4 & \rho^3 & \rho^2 & \rho & 1 & \rho & \rho^2 \\ \rho^6 & \rho^5 & \rho^4 & \rho^3 & \rho^2 & \rho & 1 & \rho \\ \rho^7 & \rho^6 & \rho^5 & \rho^4 & \rho^3 & \rho^2 & \rho & 1 \end{bmatrix}$
Spatial(power) [(sp(pow)]	$\begin{bmatrix} 1 & \rho^{d_{12}} & \rho^{d_{13}} & \rho^{d_{14}} & \rho^{d_{15}} & \rho^{d_{16}} & \rho^{d_{17}} & \rho^{d_{18}} \\ \rho^{d_{21}} & 1 & \rho^{d_{23}} & \rho^{d_{24}} & \rho^{d_{25}} & \rho^{d_{26}} & \rho^{d_{27}} & \rho^{d_{28}} \\ \rho^{d_{31}} & \rho^{d_{32}} & 1 & \rho^{d_{34}} & \rho^{d_{35}} & \rho^{d_{36}} & \rho^{d_{37}} & \rho^{d_{38}} \\ \rho^{d_{41}} & \rho^{d_{42}} & \rho^{d_{43}} & 1 & \rho^{d_{45}} & \rho^{d_{46}} & \rho^{d_{47}} & \rho^{d_{48}} \\ \rho^{d_{51}} & \rho^{d_{52}} & \rho^{d_{53}} & \rho^{d_{54}} & 1 & \rho^{d_{56}} & \rho^{d_{57}} & \rho^{d_{58}} \\ \rho^{d_{61}} & \rho^{d_{62}} & \rho^{d_{63}} & \rho^{d_{64}} & \rho^{d_{65}} & 1 & \rho^{d_{67}} & \rho^{d_{68}} \\ \rho^{d_{71}} & \rho^{d_{72}} & \rho^{d_{73}} & \rho^{d_{74}} & \rho^{d_{75}} & \rho^{d_{76}} & 1 & \rho^{d_{78}} \\ \rho^{d_{81}} & \rho^{d_{82}} & \rho^{d_{83}} & \rho^{d_{84}} & \rho^{d_{85}} & \rho^{d_{86}} & \rho^{d_{87}} & 1 \end{bmatrix}$

Toeplitz (toep)	$$\begin{bmatrix} \sigma^2 & \sigma_1 & \sigma_2 & \sigma_3 & \sigma_4 & \sigma_5 & \sigma_6 & \sigma_7 \\ \sigma_1 & \sigma^2 & \sigma_1 & \sigma_2 & \sigma_3 & \sigma_4 & \sigma_5 & \sigma_6 \\ \sigma_2 & \sigma_1 & \sigma^2 & \sigma_1 & \sigma_2 & \sigma_3 & \sigma_4 & \sigma_5 \\ \sigma_3 & \sigma_2 & \sigma_1 & \sigma^2 & \sigma_1 & \sigma_2 & \sigma_3 & \sigma_4 \\ \sigma_4 & \sigma_3 & \sigma_2 & \sigma_1 & \sigma^2 & \sigma_1 & \sigma_2 & \sigma_3 \\ \sigma_5 & \sigma_4 & \sigma_3 & \sigma_2 & \sigma_1 & \sigma^2 & \sigma_1 & \sigma_2 \\ \sigma_6 & \sigma_5 & \sigma_4 & \sigma_3 & \sigma_2 & \sigma_1 & \sigma^2 & \sigma_1 \\ \sigma_7 & \sigma_6 & \sigma_5 & \sigma_4 & \sigma_3 & \sigma_2 & \sigma_1 & \sigma^2 \end{bmatrix}$$
Unstructured (UN)	$$\begin{bmatrix} \sigma_1^2 & \sigma_{21} & \sigma_{31} & \sigma_{41} & \sigma_{51} & \sigma_{61} & \sigma_{71} & \sigma_{81} \\ \sigma_{21} & \sigma_2^2 & \sigma_{32} & \sigma_{42} & \sigma_{52} & \sigma_{62} & \sigma_{72} & \sigma_{82} \\ \sigma_{31} & \sigma_{32} & \sigma_3^2 & \sigma_{43} & \sigma_{53} & \sigma_{63} & \sigma_{73} & \sigma_{83} \\ \sigma_{41} & \sigma_{42} & \sigma_{43} & \sigma_4^2 & \sigma_{54} & \sigma_{64} & \sigma_{74} & \sigma_{84} \\ \sigma_{51} & \sigma_{52} & \sigma_{53} & \sigma_{54} & \sigma_5^2 & \sigma_{65} & \sigma_{75} & \sigma_{85} \\ \sigma_{61} & \sigma_{62} & \sigma_{63} & \sigma_{64} & \sigma_{65} & \sigma_6^2 & \sigma_{76} & \sigma_{86} \\ \sigma_{71} & \sigma_{72} & \sigma_{73} & \sigma_{74} & \sigma_{75} & \sigma_{76} & \sigma_7^2 & \sigma_{87} \\ \sigma_{81} & \sigma_{82} & \sigma_{83} & \sigma_{84} & \sigma_{85} & \sigma_{86} & \sigma_{87} & \sigma_8^2 \end{bmatrix}$$
Random Effects (RE)	For a mixed effects model with straight growth trajectories for a given drug,<br>$$Y_{ij} = (\beta_o + b_{oi}) + (\beta_1 + b_{1i})t_j + e_{ij}$$<br>Where<br>• Y_{ij} is the observed tumor ultrasound area for the ith mouse at the jth time point,<br>• β_o, β_1 are the intercept and slope mean parameters (fixed effects), respectively,<br>• b_{oi}, b_{1i} are the random intercept and slope parameters containing the ith mouse's deviations from the mean intercept and slope, respectively, and are assumed to have mean 0, variances D_{11} and D_{22}, respectively, and covariance D_{12},<br>• t_j is the day of the jth observation,<br>• e_{ij} is the within mouse error due to within mouse variation and measurement error, and is assumed to be independent of the b_{oi} and b_{1i}, and to have mean 0 and variance σ_e^2 ,<br>then<br>• $Var(Y_{ij}) = D_{11} + D_{22}t_j^2 + 2D_{12}t_j + \sigma_e^2$, and<br>• $Cov(Y_{ij}, Y_{ik}) = D_{11} + D_{22}t_j t_k + D_{12}(t_j + t_k)$.<br>The dependence on time is evident from the equations.

Supplemental Table 4. For each PDX, REML estimates of the overall variance matrix for UN, Toeplitz, RE, sp(pow)+RE, sp(pow), and CS variance structures**.** Col1 – Col8 indicate time 1 through 8 (days 0 to 28). Row 1 to 8 indicates the same. Index is the internal ID of the mouse used for printing. Variances for each time point are along the diagonals (indicated by added line in first matrix shown), and covariances are along the off-diagonals, and the top and bottom diagonals mirror each other (i.e., the matrices are symmetric). For example (cells for these examples are lightly shaded), in the PH77 UN fit, the variance at time 1 (day 0) is 0.138, and at time 8 (day 28) 0.198. The covariance between observations at time 1 and time 2 (days 0 and 4) is 0.135, and between time 1 and 8 (day 0 and 28) is 0.115.

PH77

un

Index	Row	Col1	Col2	Col3	Col4	Col5	Col6	Col7	Col8
33	1	0.138	0.135	0.124	0.120	0.135	0.125	0.149	0.115
33	2	0.135	0.178	0.167	0.170	0.188	0.183	0.186	0.134
33	3	0.124	0.167	0.178	0.169	0.170	0.165	0.164	0.122
33	4	0.120	0.170	0.169	0.235	0.243	0.217	0.190	0.151
33	5	0.135	0.188	0.170	0.243	0.365	0.346	0.275	0.204
33	6	0.125	0.183	0.165	0.217	0.346	0.396	0.258	0.195
33	7	0.149	0.186	0.164	0.190	0.275	0.258	0.294	0.208
33	8	0.115	0.134	0.122	0.151	0.204	0.195	0.208	0.198

toep

Index	Row	Col1	Col2	Col3	Col4	Col5	Col6	Col7	Col8
33	1	0.241	0.200	0.175	0.159	0.153	0.146	0.152	0.176
33	2	0.200	0.241	0.200	0.175	0.159	0.153	0.146	0.152
33	3	0.175	0.200	0.241	0.200	0.175	0.159	0.153	0.146
33	4	0.159	0.175	0.200	0.241	0.200	0.175	0.159	0.153
33	5	0.153	0.159	0.175	0.200	0.241	0.200	0.175	0.159
33	6	0.146	0.153	0.159	0.175	0.200	0.241	0.200	0.175
33	7	0.152	0.146	0.153	0.159	0.175	0.200	0.241	0.200
33	8	0.176	0.152	0.146	0.153	0.159	0.175	0.200	0.241

RE

Index	Row	Col1	Col2	Col3	Col4	Col5	Col6	Col7	Col8
33	1	0.194	0.139	0.142	0.146	0.150	0.154	0.158	0.162
33	2	0.139	0.204	0.150	0.155	0.161	0.167	0.172	0.178
33	3	0.142	0.150	0.216	0.164	0.172	0.179	0.186	0.193
33	4	0.146	0.155	0.164	0.233	0.182	0.191	0.200	0.209
33	5	0.150	0.161	0.172	0.182	0.252	0.203	0.214	0.224
33	6	0.154	0.167	0.179	0.191	0.203	0.275	0.228	0.240
33	7	0.158	0.172	0.186	0.200	0.214	0.228	0.301	0.255
33	8	0.162	0.178	0.193	0.209	0.224	0.240	0.255	0.330

sppow+RE

Index	Row	Col1	Col2	Col3	Col4	Col5	Col6	Col7	Col8
33	1	0.231	0.191	0.167	0.152	0.144	0.139	0.136	0.134
33	2	0.191	0.231	0.191	0.167	0.152	0.144	0.139	0.136
33	3	0.167	0.191	0.231	0.191	0.167	0.152	0.144	0.139
33	4	0.152	0.167	0.191	0.231	0.191	0.167	0.152	0.144
33	5	0.144	0.152	0.167	0.191	0.231	0.191	0.167	0.152
33	6	0.139	0.144	0.152	0.167	0.191	0.231	0.191	0.167
33	7	0.136	0.139	0.144	0.152	0.167	0.191	0.231	0.191
33	8	0.134	0.136	0.139	0.144	0.152	0.167	0.191	0.231

sppow

Index	Row	Col1	Col2	Col3	Col4	Col5	Col6	Col7	Col8
33	1	0.220	0.179	0.145	0.118	0.096	0.078	0.064	0.052
33	2	0.179	0.220	0.179	0.145	0.118	0.096	0.078	0.064
33	3	0.145	0.179	0.220	0.179	0.145	0.118	0.096	0.078
33	4	0.118	0.145	0.179	0.220	0.179	0.145	0.118	0.096
33	5	0.096	0.118	0.145	0.179	0.220	0.179	0.145	0.118
33	6	0.078	0.096	0.118	0.145	0.179	0.220	0.179	0.145
33	7	0.064	0.078	0.096	0.118	0.145	0.179	0.220	0.179
33	8	0.052	0.064	0.078	0.096	0.118	0.145	0.179	0.220

cs

Index	Row	Col1	Col2	Col3	Col4	Col5	Col6	Col7	Col8
33	1	0.249	0.180	0.180	0.180	0.180	0.180	0.180	0.180
33	2	0.180	0.249	0.180	0.180	0.180	0.180	0.180	0.180
33	3	0.180	0.180	0.249	0.180	0.180	0.180	0.180	0.180
33	4	0.180	0.180	0.180	0.249	0.180	0.180	0.180	0.180
33	5	0.180	0.180	0.180	0.180	0.249	0.180	0.180	0.180
33	6	0.180	0.180	0.180	0.180	0.180	0.249	0.180	0.180
33	7	0.180	0.180	0.180	0.180	0.180	0.180	0.249	0.180
33	8	0.180	0.180	0.180	0.180	0.180	0.180	0.180	0.249

PH80

un

Index	Row	Col1	Col2	Col3	Col4	Col5	Col6	Col7	Col8
25	1	0.159	0.079	0.036	0.036	0.027	0.035	0.029	0.038
25	2	0.079	0.079	0.045	0.070	0.050	0.042	0.044	0.046
25	3	0.036	0.045	0.075	0.085	0.081	0.100	0.110	0.087
25	4	0.036	0.070	0.085	0.173	0.136	0.144	0.159	0.142
25	5	0.027	0.050	0.081	0.136	0.130	0.151	0.155	0.129
25	6	0.035	0.042	0.100	0.144	0.151	0.263	0.252	0.130
25	7	0.029	0.044	0.110	0.159	0.155	0.252	0.288	0.126
25	8	0.038	0.046	0.087	0.142	0.129	0.130	0.126	0.205

toep

Index	Row	Col1	Col2	Col3	Col4	Col5	Col6	Col7	Col8
25	1	0.169	0.124	0.099	0.089	0.072	0.053	0.041	0.030
25	2	0.124	0.169	0.124	0.099	0.089	0.072	0.053	0.041
25	3	0.099	0.124	0.169	0.124	0.099	0.089	0.072	0.053
25	4	0.089	0.099	0.124	0.169	0.124	0.099	0.089	0.072
25	5	0.072	0.089	0.099	0.124	0.169	0.124	0.099	0.089
25	6	0.053	0.072	0.089	0.099	0.124	0.169	0.124	0.099
25	7	0.041	0.053	0.072	0.089	0.099	0.124	0.169	0.124
25	8	0.030	0.041	0.053	0.072	0.089	0.099	0.124	0.169

RE

Index	Row	Col1	Col2	Col3	Col4	Col5	Col6	Col7	Col8
25	1	0.112	0.057	0.055	0.052	0.050	0.048	0.045	0.043
25	2	0.057	0.112	0.062	0.064	0.066	0.068	0.070	0.072
25	3	0.055	0.062	0.121	0.075	0.081	0.088	0.094	0.101
25	4	0.052	0.064	0.075	0.138	0.097	0.108	0.119	0.130
25	5	0.050	0.066	0.081	0.097	0.165	0.128	0.144	0.159
25	6	0.048	0.068	0.088	0.108	0.128	0.201	0.168	0.188
25	7	0.045	0.070	0.094	0.119	0.144	0.168	0.246	0.218
25	8	0.043	0.072	0.101	0.130	0.159	0.188	0.218	0.299

sppow+RE

Index	Row	Col1	Col2	Col3	Col4	Col5	Col6	Col7	Col8
25	1	0.170	0.126	0.097	0.078	0.066	0.058	0.052	0.049
25	2	0.126	0.170	0.126	0.097	0.078	0.066	0.058	0.052
25	3	0.097	0.126	0.170	0.126	0.097	0.078	0.066	0.058
25	4	0.078	0.097	0.126	0.170	0.126	0.097	0.078	0.066
25	5	0.066	0.078	0.097	0.126	0.170	0.126	0.097	0.078

25	6	0.058	0.066	0.078	0.097	0.126	0.170	0.126	0.097
25	7	0.052	0.058	0.066	0.078	0.097	0.126	0.170	0.126
25	8	0.049	0.052	0.058	0.066	0.078	0.097	0.126	0.170

sppow

Index	Row	Col1	Col2	Col3	Col4	Col5	Col6	Col7	Col8
25	1	0.173	0.129	0.096	0.072	0.053	0.040	0.030	0.022
25	2	0.129	0.173	0.129	0.096	0.072	0.053	0.040	0.030
25	3	0.096	0.129	0.173	0.129	0.096	0.072	0.053	0.040
25	4	0.072	0.096	0.129	0.173	0.129	0.096	0.072	0.053
25	5	0.053	0.072	0.096	0.129	0.173	0.129	0.096	0.072
25	6	0.040	0.053	0.072	0.096	0.129	0.173	0.129	0.096
25	7	0.030	0.040	0.053	0.072	0.096	0.129	0.173	0.129
25	8	0.022	0.030	0.040	0.053	0.072	0.096	0.129	0.173

cs

Index	Row	Col1	Col2	Col3	Col4	Col5	Col6	Col7	Col8
25	1	0.163	0.089	0.089	0.089	0.089	0.089	0.089	0.089
25	2	0.089	0.163	0.089	0.089	0.089	0.089	0.089	0.089
25	3	0.089	0.089	0.163	0.089	0.089	0.089	0.089	0.089
25	4	0.089	0.089	0.089	0.163	0.089	0.089	0.089	0.089
25	5	0.089	0.089	0.089	0.089	0.163	0.089	0.089	0.089
25	6	0.089	0.089	0.089	0.089	0.089	0.163	0.089	0.089
25	7	0.089	0.089	0.089	0.089	0.089	0.089	0.163	0.089
25	8	0.089	0.089	0.089	0.089	0.089	0.089	0.089	0.163

PH87

Table: un

Did not converge

toep

Index	Row	Col1	Col2	Col3	Col4	Col5	Col6	Col7	Col8
21	1	0.134	0.108	0.092	0.083	0.069	0.058	0.059	0.032
21	2	0.108	0.134	0.108	0.092	0.083	0.069	0.058	0.059
21	3	0.092	0.108	0.134	0.108	0.092	0.083	0.069	0.058
21	4	0.083	0.092	0.108	0.134	0.108	0.092	0.083	0.069
21	5	0.069	0.083	0.092	0.108	0.134	0.108	0.092	0.083
21	6	0.058	0.069	0.083	0.092	0.108	0.134	0.108	0.092
21	7	0.059	0.058	0.069	0.083	0.092	0.108	0.134	0.108
21	8	0.032	0.059	0.058	0.069	0.083	0.092	0.108	0.134

RE

Index	Row	Col1	Col2	Col3	Col4	Col5	Col6	Col7	Col8
21	1	0.113	0.080	0.077	0.074	0.072	0.069	0.066	0.063
21	2	0.080	0.111	0.082	0.083	0.084	0.085	0.086	0.087
21	3	0.077	0.082	0.117	0.091	0.096	0.101	0.106	0.110
21	4	0.074	0.083	0.091	0.130	0.108	0.117	0.126	0.134
21	5	0.072	0.084	0.096	0.108	0.151	0.133	0.145	0.158
21	6	0.069	0.085	0.101	0.117	0.133	0.180	0.165	0.181
21	7	0.066	0.086	0.106	0.126	0.145	0.165	0.215	0.205
21	8	0.063	0.087	0.110	0.134	0.158	0.181	0.205	0.259

sppow+RE

Index	Row	Col1	Col2	Col3	Col4	Col5	Col6	Col7	Col8
21	1	0.132	0.107	0.089	0.077	0.068	0.062	0.057	0.054
21	2	0.107	0.132	0.107	0.089	0.077	0.068	0.062	0.057
21	3	0.089	0.107	0.132	0.107	0.089	0.077	0.068	0.062
21	4	0.077	0.089	0.107	0.132	0.107	0.089	0.077	0.068
21	5	0.068	0.077	0.089	0.107	0.132	0.107	0.089	0.077
21	6	0.062	0.068	0.077	0.089	0.107	0.132	0.107	0.089
21	7	0.057	0.062	0.068	0.077	0.089	0.107	0.132	0.107
21	8	0.054	0.057	0.062	0.068	0.077	0.089	0.107	0.132

sppow

Index	Row	Col1	Col2	Col3	Col4	Col5	Col6	Col7	Col8
21	1	0.132	0.107	0.086	0.070	0.056	0.046	0.037	0.030
21	2	0.107	0.132	0.107	0.086	0.070	0.056	0.046	0.037
21	3	0.086	0.107	0.132	0.107	0.086	0.070	0.056	0.046

21	4	0.070	0.086	0.107	0.132	0.107	0.086	0.070	0.056
21	5	0.056	0.070	0.086	0.107	0.132	0.107	0.086	0.070
21	6	0.046	0.056	0.070	0.086	0.107	0.132	0.107	0.086
21	7	0.037	0.046	0.056	0.070	0.086	0.107	0.132	0.107
21	8	0.030	0.037	0.046	0.056	0.070	0.086	0.107	0.132

cs

Index	Row	Col1	Col2	Col3	Col4	Col5	Col6	Col7	Col8
21	1	0.130	0.084	0.084	0.084	0.084	0.084	0.084	0.084
21	2	0.084	0.130	0.084	0.084	0.084	0.084	0.084	0.084
21	3	0.084	0.084	0.130	0.084	0.084	0.084	0.084	0.084
21	4	0.084	0.084	0.084	0.130	0.084	0.084	0.084	0.084
21	5	0.084	0.084	0.084	0.084	0.130	0.084	0.084	0.084
21	6	0.084	0.084	0.084	0.084	0.084	0.130	0.084	0.084
21	7	0.084	0.084	0.084	0.084	0.084	0.084	0.130	0.084
21	8	0.084	0.084	0.084	0.084	0.084	0.084	0.084	0.130

PH95

un

Index	Row	Col1	Col2	Col3	Col4	Col5	Col6	Col7	Col8
22	1	0.075	0.085	0.077	0.065	0.055	0.079	0.061	0.110
22	2	0.085	0.153	0.140	0.098	0.093	0.156	0.123	0.173
22	3	0.077	0.140	0.158	0.094	0.098	0.165	0.125	0.175
22	4	0.065	0.098	0.094	0.094	0.084	0.124	0.106	0.164
22	5	0.055	0.093	0.098	0.084	0.106	0.142	0.100	0.140
22	6	0.079	0.156	0.165	0.124	0.142	0.254	0.175	0.257
22	7	0.061	0.123	0.125	0.106	0.100	0.175	0.154	0.206
22	8	0.110	0.173	0.175	0.164	0.140	0.257	0.206	0.363

toep

Index	Row	Col1	Col2	Col3	Col4	Col5	Col6	Col7	Col8
22	1	0.184	0.155	0.146	0.143	0.138	0.124	0.120	0.110
22	2	0.155	0.184	0.155	0.146	0.143	0.138	0.124	0.120
22	3	0.146	0.155	0.184	0.155	0.146	0.143	0.138	0.124
22	4	0.143	0.146	0.155	0.184	0.155	0.146	0.143	0.138
22	5	0.138	0.143	0.146	0.155	0.184	0.155	0.146	0.143
22	6	0.124	0.138	0.143	0.146	0.155	0.184	0.155	0.146
22	7	0.120	0.124	0.138	0.143	0.146	0.155	0.184	0.155
22	8	0.110	0.120	0.124	0.138	0.143	0.146	0.155	0.184

RE

Index	Row	Col1	Col2	Col3	Col4	Col5	Col6	Col7	Col8
22	1	0.100	0.074	0.079	0.085	0.090	0.095	0.100	0.106
22	2	0.074	0.113	0.089	0.097	0.105	0.112	0.120	0.127
22	3	0.079	0.089	0.130	0.109	0.119	0.129	0.139	0.149
22	4	0.085	0.097	0.109	0.152	0.134	0.146	0.159	0.171
22	5	0.090	0.105	0.119	0.134	0.179	0.163	0.178	0.193
22	6	0.095	0.112	0.129	0.146	0.163	0.211	0.197	0.214
22	7	0.100	0.120	0.139	0.159	0.178	0.197	0.248	0.236
22	8	0.106	0.127	0.149	0.171	0.193	0.214	0.236	0.289

sppow+RE

Index	Row	Col1	Col2	Col3	Col4	Col5	Col6	Col7	Col8
22	1	0.169	0.140	0.125	0.119	0.115	0.114	0.113	0.113
22	2	0.140	0.169	0.140	0.125	0.119	0.115	0.114	0.113
22	3	0.125	0.140	0.169	0.140	0.125	0.119	0.115	0.114
22	4	0.119	0.125	0.140	0.169	0.140	0.125	0.119	0.115
22	5	0.115	0.119	0.125	0.140	0.169	0.140	0.125	0.119

22	6	0.114	0.115	0.119	0.125	0.140	0.169	0.140	0.125
22	7	0.113	0.114	0.115	0.119	0.125	0.140	0.169	0.140
22	8	0.113	0.113	0.114	0.115	0.119	0.125	0.140	0.169

sppow

Index	Row	Col1	Col2	Col3	Col4	Col5	Col6	Col7	Col8
22	1	0.177	0.148	0.123	0.103	0.086	0.071	0.060	0.050
22	2	0.148	0.177	0.148	0.123	0.103	0.086	0.071	0.060
22	3	0.123	0.148	0.177	0.148	0.123	0.103	0.086	0.071
22	4	0.103	0.123	0.148	0.177	0.148	0.123	0.103	0.086
22	5	0.086	0.103	0.123	0.148	0.177	0.148	0.123	0.103
22	6	0.071	0.086	0.103	0.123	0.148	0.177	0.148	0.123
22	7	0.060	0.071	0.086	0.103	0.123	0.148	0.177	0.148
22	8	0.050	0.060	0.071	0.086	0.103	0.123	0.148	0.177

cs

Index	Row	Col1	Col2	Col3	Col4	Col5	Col6	Col7	Col8
22	1	0.165	0.123	0.123	0.123	0.123	0.123	0.123	0.123
22	2	0.123	0.165	0.123	0.123	0.123	0.123	0.123	0.123
22	3	0.123	0.123	0.165	0.123	0.123	0.123	0.123	0.123
22	4	0.123	0.123	0.123	0.165	0.123	0.123	0.123	0.123
22	5	0.123	0.123	0.123	0.123	0.165	0.123	0.123	0.123
22	6	0.123	0.123	0.123	0.123	0.123	0.165	0.123	0.123
22	7	0.123	0.123	0.123	0.123	0.123	0.123	0.165	0.123
22	8	0.123	0.123	0.123	0.123	0.123	0.123	0.123	0.165

PH39

un

Index	Row	Col1	Col2	Col3	Col4	Col5	Col6	Col7	Col8
19	1	0.133	0.091	0.108	0.160	0.178	0.079	0.069	0.053
19	2	0.091	0.099	0.129	0.141	0.155	0.092	0.063	0.048
19	3	0.108	0.129	0.314	0.332	0.198	0.158	0.063	0.014
19	4	0.160	0.141	0.332	0.487	0.269	0.207	0.103	-0.006
19	5	0.178	0.155	0.198	0.269	0.519	0.467	0.468	0.202
19	6	0.079	0.092	0.158	0.207	0.467	0.766	0.741	0.437
19	7	0.069	0.063	0.063	0.103	0.468	0.741	0.793	0.440
19	8	0.053	0.048	0.014	-0.006	0.202	0.437	0.440	0.427

toep

Index	Row	Col1	Col2	Col3	Col4	Col5	Col6	Col7	Col8
19	1	0.350	0.251	0.188	0.153	0.113	0.102	0.115	0.055
19	2	0.251	0.350	0.251	0.188	0.153	0.113	0.102	0.115
19	3	0.188	0.251	0.350	0.251	0.188	0.153	0.113	0.102
19	4	0.153	0.188	0.251	0.350	0.251	0.188	0.153	0.113
19	5	0.113	0.153	0.188	0.251	0.350	0.251	0.188	0.153
19	6	0.102	0.113	0.153	0.188	0.251	0.350	0.251	0.188
19	7	0.115	0.102	0.113	0.153	0.188	0.251	0.350	0.251
19	8	0.055	0.115	0.102	0.113	0.153	0.188	0.251	0.350

RE

Index	Row	Col1	Col2	Col3	Col4	Col5	Col6	Col7	Col8
19	1	0.264	0.133	0.131	0.129	0.127	0.125	0.123	0.122
19	2	0.133	0.267	0.143	0.148	0.153	0.158	0.163	0.168
19	3	0.131	0.143	0.284	0.166	0.178	0.190	0.202	0.214
19	4	0.129	0.148	0.166	0.315	0.204	0.222	0.241	0.260
19	5	0.127	0.153	0.178	0.204	0.359	0.255	0.280	0.306
19	6	0.125	0.158	0.190	0.222	0.255	0.417	0.320	0.352
19	7	0.123	0.163	0.202	0.241	0.280	0.320	0.488	0.398
19	8	0.122	0.168	0.214	0.260	0.306	0.352	0.398	0.574

sppow+RE

Index	Row	Col1	Col2	Col3	Col4	Col5	Col6	Col7	Col8
19	1	0.346	0.248	0.186	0.147	0.122	0.107	0.097	0.091
19	2	0.248	0.346	0.248	0.186	0.147	0.122	0.107	0.097
19	3	0.186	0.248	0.346	0.248	0.186	0.147	0.122	0.107
19	4	0.147	0.186	0.248	0.346	0.248	0.186	0.147	0.122
19	5	0.122	0.147	0.186	0.248	0.346	0.248	0.186	0.147

19	6	0.107	0.122	0.147	0.186	0.248	0.346	0.248	0.186
19	7	0.097	0.107	0.122	0.147	0.186	0.248	0.346	0.248
19	8	0.091	0.097	0.107	0.122	0.147	0.186	0.248	0.346

sppow

Index	Row	Col1	Col2	Col3	Col4	Col5	Col6	Col7	Col8
19	1	0.347	0.249	0.178	0.128	0.092	0.066	0.047	0.034
19	2	0.249	0.347	0.249	0.178	0.128	0.092	0.066	0.047
19	3	0.178	0.249	0.347	0.249	0.178	0.128	0.092	0.066
19	4	0.128	0.178	0.249	0.347	0.249	0.178	0.128	0.092
19	5	0.092	0.128	0.178	0.249	0.347	0.249	0.178	0.128
19	6	0.066	0.092	0.128	0.178	0.249	0.347	0.249	0.178
19	7	0.047	0.066	0.092	0.128	0.178	0.249	0.347	0.249
19	8	0.034	0.047	0.066	0.092	0.128	0.178	0.249	0.347

cs

Index	Row	Col1	Col2	Col3	Col4	Col5	Col6	Col7	Col8
19	1	0.354	0.191	0.191	0.191	0.191	0.191	0.191	0.191
19	2	0.191	0.354	0.191	0.191	0.191	0.191	0.191	0.191
19	3	0.191	0.191	0.354	0.191	0.191	0.191	0.191	0.191
19	4	0.191	0.191	0.191	0.354	0.191	0.191	0.191	0.191
19	5	0.191	0.191	0.191	0.191	0.354	0.191	0.191	0.191
19	6	0.191	0.191	0.191	0.191	0.191	0.354	0.191	0.191
19	7	0.191	0.191	0.191	0.191	0.191	0.191	0.354	0.191
19	8	0.191	0.191	0.191	0.191	0.191	0.191	0.191	0.354
